# Bioactive Fraction LCS2 from Litchi (*Litchi chinensis* *Sonn.*) Seed By-Product Attenuates LPS-Induced Acute Prostatic Inflammatory Injury via Modulating NF-κB and MAPK Signaling Pathways

**DOI:** 10.3390/foods15193524

**Published:** 2026-10-02

**Authors:** Yuping Li, Yingjie Huang, Qinqin Wang, Junmao Li, Jiaping Pan, Renyikun Yuan, Hongwei Gao, Lichun Zhao

**Affiliations:** 1 School of Pharmacy, Guizhou University of Traditional Chinese Medicine, Guiyang 550025, China; 2 School of Basic Medicine, Guizhou University of Traditional Chinese Medicine, Guiyang 550025, China; 3 Institute of Traditional Chinese and Zhuang-Yao Ethnic Medicine, Guangxi University of Chinese Medicine, Nanning 530200, China; 4 School of Pharmacy, Jiangxi University of Traditional Chinese Medicine, Nanchang 330004, China

**Keywords:** litchi seed by-product, polyphenol-rich fraction, by-product valorization, functional food ingredient, prostatic health, anti-inflammatory activity, NF-κB, MAPK

## Abstract

Litchi seed is a major by-product of the litchi processing industry, rich in polyphenols and flavonoids with documented anti-inflammatory properties. Prostatic inflammatory injury remains a prevalent male health concern, and dietary intervention with natural bioactive components represents a promising strategy for prostate health maintenance. This study investigated the protective efficacy of LCS2, a bioactive fraction isolated from litchi seed by-product, against lipopolysaccharide (LPS)-induced prostatic inflammatory injury in rats and explored its underlying mechanisms. LCS2 was obtained via extraction, purification, and activity screening, and its chemical profile was characterized by ultra-performance liquid chromatography coupled with quadrupole time-of-flight tandem mass spectrometry (UPLC-Q-TOF-MS/MS). Its anti-inflammatory activity and pathway regulatory effects were evaluated in LPS-stimulated RAW264.7 macrophages and further verified in a rat model of LPS-induced acute prostatic inflammation. A total of 26 compounds, mainly flavonoids, polyphenols, and tannins, were tentatively identified in LCS2. In vitro, LCS2 pretreatment with LCS2 at concentrations up to 40 μg/mL dose-dependently suppressed LPS-induced overproduction of nitric oxide (NO) and reactive oxygen species (ROS), reduced pro-inflammatory cytokine mRNA levels, and downregulated iNOS and COX-2 protein expression. In vivo, 7-day dietary pretreatment with LCS2 at doses up to 100 mg/kg ameliorated prostate histopathological injury, reduced peripheral inflammatory cell counts, and decreased inflammatory mediator levels in serum and prostate tissue by inhibiting NF-κB and MAPK pathway activation. These findings support the high-value valorization of litchi seed by-product and highlight the potential of LCS2 for further development as a bioactive component for prostate health applications.

## 1. Introduction

The litchi (*Litchi chinensis* Sonn., Sapindaceae) processing industry generates large quantities of by-products annually, including seeds, pericarps and leaves, most of which are directly discarded and cause substantial resource waste and environmental pressure. Accumulating studies have demonstrated that litchi seeds are rich in bioactive constituents including saponins, flavonoids (e.g., quercetin and rutin) [1], and polyphenols (e.g., procyanidin A1 and A2) [2], indicating their considerable potential for development as functional food ingredients. Prostatitis, a common inflammatory condition of the prostate gland, is a major concern in male urological health [3]. Dietary intervention with natural bioactive components has emerged as a safe and accessible strategy for the prevention and auxiliary improvement of prostatic inflammatory injury. Accumulating evidence indicates that inflammation-driven NF-κB and MAPK signaling pathways are critically involved in the pathogenesis of chronic pelvic pain and prostatitis [4,5,6,7]. NF-κB is a pivotal transcription factor that regulates a wide range of cellular inflammatory responses and drives the production of numerous inflammatory cytokines and mediators. Under physiological conditions, the p65 and p50 subunits of NF-κB are sequestered in the cytoplasm in the form of inactive NF-κB/IκB complexes [8,9]. JNK, p38 MAPK, and ERK1/2 are the core members of the MAPK family. Therefore, suppressing the activation of these key signaling pathways represents a promising strategy for screening dietary bioactive ingredients with prostate health benefits [10,11,12].

Previous studies have demonstrated that prostate tissue injury, characterized by prominent inflammatory cell infiltration, interstitial edema, and stromal fibrosis, constitutes the pathological basis of prostatitis [13]. Upon exposure to bacteria or other pro-inflammatory stimuli, resident inflammatory cells and target cells in the prostate become activated [14]. These cells act in concert to eliminate pathogens and infected cells by secreting chemokines and inflammatory mediators, including nitric oxide (NO), reactive oxygen species (ROS), cyclooxygenase-2 (COX-2), inducible nitric oxide synthase (iNOS), tumor necrosis factor-α (TNF-α), interleukin-1β (IL-1β), interleukin-6 (IL-6), and prostaglandin E2 (PGE2) [15,16]. The inflammatory mediators released by these cells induce oxidative stress in the prostate tissue microenvironment [17,18], resulting in characteristic tissue damage in prostatitis and further activating the downstream NF-κB and MAPK signaling pathways [19,20,21].

Lipopolysaccharide (LPS), a major component of the outer membrane of Gram-negative bacteria such as *Escherichia coli*, is a potent endotoxin and one of the most robust macrophage activators, which is widely utilized to establish cellular and animal inflammatory models [22,23]. Previous studies have shown that LPS stimulation can induce chronic non-bacterial prostatitis-like phenotypes in RWPE-1 cells [24], and intraprostatic or systemic injection of LPS in rodents can reliably establish prostatitis models [25,26]. LPS binds to pattern recognition receptors on the cell surface, triggers intracellular signaling cascades, and induces the activation of NF-κB and MAPK-mediated pathways. These pathways not only mediate homeostatic anti-inflammatory processes but also drive excessive inflammation, oxidative stress, and inflammatory cascade amplification. In response to inflammatory stimuli, activated transcription factors translocate into the nucleus, where they upregulate the transcription of pro-inflammatory genes such as COX-2 and iNOS [27,28].

Litchi, a plant belonging to the Sapindaceae family, has been cultivated in China for nearly 2000 years, dating back to the Han Dynasty, and has long been known as the “King of Fruits”. Currently, litchi is commercially cultivated in over 20 countries worldwide [29]. The litchi industry holds considerable economic importance globally, contributing substantially to agricultural output in producing regions [30]. Despite the economic value of the litchi industry, litchi seeds, leaves, and pericarps are generally discarded as agricultural by-products. Existing studies on litchi seed bioactivity have mostly focused on crude extracts or total flavonoid/polyphenol fractions, with reported health benefits including anti-inflammatory, antiviral, antioxidant, and anticancer effects [31,32,33]. However, few studies have systematically isolated and screened bioactive fractions from litchi seeds targeting prostatic inflammatory injury, and the specific active fraction responsible for prostate-protective effects and its underlying mechanism remain unclear. Our previous work showed that the *n*-butanol fraction of litchi seed extract was enriched in polyphenols and exhibited notable bioactivity [34]; after further gradient purification and anti-inflammatory activity screening, the 30% ethanol-eluted fraction (designated LCS2) showed the most potent anti-inflammatory activity among all obtained fractions. Accordingly, the present study systematically investigated the protective efficacy of LCS2 against LPS-induced prostatic inflammatory injury and explored its underlying molecular mechanisms, aiming to provide a scientific basis for the high-value utilization of litchi seed by-products and the development of natural dietary ingredients for prostate health.

## 2. Materials and Methods

### 2.1. Plant Material

Dried mature seeds of *Litchi chinensis* Sonn. (Sapindaceae), collected as a processing by-product from the litchi industry, were collected in Hepu County, Beihai City, Guangxi Zhuang Autonomous Region, China, in July 2023 and provided by Hepu Fruit Garden Food Co., Ltd. (Beihai, China). The botanical identity of the seeds was authenticated by Prof. Jingquan Yuan, a senior botanist at the Scientific Experimental Center of Guangxi University of Chinese Medicine, as the dried mature seeds of *Litchi chinensis* Sonn. (Sapindaceae). A voucher specimen (No. LCS-2023001) has been deposited in the herbarium of the Institute of Traditional Chinese and Zhuang-Yao Ethnic Medicine, Guangxi University of Chinese Medicine, and is stored at room temperature in a dry, dark environment for future reference. The collection of litchi seeds complied with local biodiversity regulations of Guangxi Zhuang Autonomous Region, China. No protected or endangered plant species were involved in this study.

### 2.2. Reagents and Antibodies

All chemical solvents and reagents were of analytical grade or high-performance liquid chromatography (HPLC) grade unless otherwise specified. Anhydrous ethanol, petroleum ether, ethyl acetate, and *n*-butanol were purchased from Chengdu Chron Chemicals Co., Ltd. (Chengdu, China); lipopolysaccharide (LPS, from *Escherichia coli* O111:B4, Cat. No. 4359) was obtained from Sigma-Aldrich (St. Louis, MO, USA); and dimethyl sulfoxide (DMSO, analytical grade, lot no. C15765609) was purchased from Shanghai Macklin Biochemical Technology Co., Ltd. (Shanghai, China). TRIzol reagent (Cat. No. 15596026) was purchased from Invitrogen (Carlsbad, CA, USA); the SweScript RT I First Strand cDNA Synthesis Kit (Cat. No. G3330) was obtained from Servicebio (Wuhan, China); RIPA protein extraction kit (Cat. No. P0013B) and BCA protein assay kit (Cat. No. P0010) were supplied by Beyotime Biotechnology (Shanghai, China); DCFH_2_-DA (Cat. No. D6883) and Hoechst 33342 (Cat. No. B2261) were purchased from Sigma-Aldrich; and citrate antigen retrieval solution (pH 6.0, Cat. No. G1202) and DAB chromogenic kit (Cat. No. G1211) were provided by Servicebio (Wuhan, China).

For primary antibodies, a panel of antibodies for Western blotting (WB), including COX-2 (Cat. No. 4842), inducible nitric oxide synthase (iNOS, Cat. No. 7835), p65 (Cat. No. 8242), phospho-NF-κB p65 (p-p65, Cat. No. 3033), IKKα (Cat. No. 2682), IKKβ (Cat. No. 8943T), phospho-IKKα/β (p-IKKα/β, Cat. No. 2697), IκBα (Cat. No. 4814), phospho-IκBα (p-IκBα, Cat. No. 2859), JNK (Cat. No. 9252T), phospho-JNK (p-JNK, Cat. No. 4668T), ERK (Cat. No. 4695T), phospho-ERK (p-ERK, Cat. No. 4370T), p38 mitogen-activated protein kinase (p38 MAPK, Cat. No. 9212), phospho-p38 MAPK (p-p38, Cat. No. 4511), and GAPDH (Cat. No. 2118), was purchased from Cell Signaling Technology (CST, Danvers, MA, USA); primary antibodies used for immunohistochemistry (IHC), including prostaglandin E2 (PGE2, Cat. No. GB111814), phospho-NF-κB p65 (p-p65, Cat. No. GB113882), tumor necrosis factor-α (TNF-α, Cat. No. GB11188), and interleukin-6 (IL-6, Cat. No. GB11117), were obtained from Wuhan Servicebio Technology Co., Ltd. (Servicebio, Wuhan, China); and the primary antibody for immunofluorescence (IF) staining, NF-κB p65 (Cat. No. 8242), was sourced from CST. For secondary antibodies, horseradish peroxidase (HRP)-linked anti-rabbit IgG (Cat. No. 7074, CST) was used for WB, HRP-conjugated goat anti-rabbit IgG (Cat. No. GB23303, Servicebio) was applied for IHC, and Alexa Fluor 488-conjugated goat anti-rabbit IgG (Cat. No. 4412, CST) was used for IF detection.

In addition, all primer pairs for quantitative real-time polymerase chain reaction (RT-qPCR), including TNF-α (forward: 5′-CTGAACTTCGGGGTGATCGG-3′, reverse: 5′-GGCTTGTCACTCGAATTTTGAGA-3′, Cat. No. 6387), IL-6 (forward: 5′-CTGCAAGAGACTTCCATCCAG-3′, reverse: 5′-AGTGGTATAGACAGGTCTGTTGG-3′, Cat. No. 6954), IL-1β (forward: 5′-GAAATGCCACCTTTTGACAGTG-3′, reverse: 5′-TGGATGCTCTCATCAGGACAG-3′, Cat. No. 4936), and GAPDH (forward: 5′-CCTCGTCCCGTAGACAAAATG-3′, reverse: 5′-TCTCCACTTTGCCACTGCAA-3′, Cat. No. 6886), were synthesized by Servicebio (Wuhan, China).

### 2.3. Preparation and Fractionation of Litchi Seed Extract

Briefly, 15 kg of dried litchi seeds were weighed, crushed into coarse powder, and extracted via reflux extraction with 8 volumes (*v*/*w*) of 70% (*v*/*v*) ethanol three times, each lasting 1.5 h. The filtrates were combined and concentrated under reduced pressure to remove ethanol until no alcoholic odor was detectable, yielding the crude litchi seed extract [35]. Water was added to the extract to improve its solubility, and the aqueous suspension was sequentially extracted with equal volumes of petroleum ether, ethyl acetate, and *n*-butanol three times each. The organic solvents were recovered to obtain the petroleum ether fraction (77 g), ethyl acetate fraction (244 g), *n*-butanol fraction (326 g), and water fraction.

Previous phytochemical investigations have revealed that the primary bioactive constituents of litchi seed include flavonoids, polyphenols, and tannins, among others [33]. Our previous studies demonstrated that the *n*-butanol fraction could efficiently enrich these key bioactive components, and its anti-prostate cancer bioactivity was markedly superior to that of the ethyl acetate, petroleum ether, and aqueous fractions [34]. Accordingly, the *n*-butanol fraction was selected for further separation and purification via gradient elution using D101 macroporous adsorption resin in the present study.

Briefly, 200 g of the *n*-butanol fraction was dissolved in a minimal volume of purified water and loaded onto a D101 macroporous resin column (Φ 5.0 cm × 60 cm, bed volume ~1200 mL, Guangxi Nanning Chenze Experimental Technology Co., Ltd. (Nanning, China) [36]. Sequential gradient elution was performed with purified water and 30%, 50%, 70%, and 95% (*v*/*v*) ethanol solutions. Each gradient was eluted with 3 bed volumes of solvent at a flow rate of 2 bed volumes (BV)/h. The eluates of each gradient were collected and concentrated under reduced pressure, followed by lyophilization to obtain five dried fractions, designated LCS1, LCS2, LCS3, LCS4, and LCS5. The recovered dry mass of the LCS2 fraction (30% ethanol eluate) was 42.6 g, corresponding to a yield of 21.3% (*w*/*w*) relative to the loaded *n*-butanol fraction. With respect to the initial 15 kg of dried litchi seed raw material, the overall yield of LCS2 was approximately 0.46% (*w*/*w*).

LCS1, eluted with purified water, was discarded as it mainly contained highly polar impurities such as inorganic salts, small-molecule sugars, and amino acids, which were considered to lack anti-inflammatory activity and would interfere with subsequent experiments. LCS5, eluted with 95% ethanol, was discarded as it mainly contained strongly hydrophobic impurities such as lipids, waxes, and residual macromolecular substances with low bioactivity, which were deemed unrelated to anti-inflammatory effects.

Preliminary anti-inflammatory activity screening was conducted on LCS2 (30% ethanol eluate), LCS3 (50% ethanol eluate), and LCS4 (70% ethanol eluate). The screening results indicated that LCS2 exhibited the most potent anti-inflammatory activity among the three fractions; thus, LCS2 was selected for subsequent in vitro and in vivo experiments.

### 2.4. Cell Culture

RAW264.7 murine macrophage cells were obtained from the Cell Bank of the Chinese Academy of Sciences (Shanghai, China) and cultured in Dulbecco’s Modified Eagle Medium (DMEM) supplemented with 1% penicillin–streptomycin solution and 10% fetal bovine serum (FBS). Cells were maintained in a humidified incubator at 37 °C with 5% CO_2_. RAW264.7 murine macrophages are a classic cellular model widely used for preliminary screening of the general anti-inflammatory activity of natural products and for exploring universal inflammatory signaling pathways [37]. Since macrophages serve as key effector cells in the initiation and amplification of inflammatory responses, including prostatic inflammation [38], we utilized this cell line to preliminarily evaluate the anti-inflammatory potential and possible pathway regulation of LCS2. It should be noted that the in vitro macrophage data only reflect general anti-inflammatory activity and cannot fully represent cell-type-specific responses in prostate tissue; the in vivo anti-prostatitis effect and associated mechanisms were further verified in the rat model.

### 2.5. Cell Viability Assay

Cell viability was assessed using the 3-(4,5-dimethylthiazol-2-yl)-2,5-diphenyltetrazolium bromide (MTT) assay. RAW264.7 cells were seeded into 96-well plates at a density of 3 × 10^4^ cells per well and incubated overnight. After overnight incubation, cells were treated with LCS2 (10, 20, 40 μg/mL), LCS3 (2, 4, 8 μg/mL), or LCS4 (0.2, 0.4, 0.8 μg/mL) for 24 h. Subsequently, 10 μL of MTT solution (5 mg/mL) was added to each well, and the plates were incubated at 37 °C for an additional 3 h.

After incubation, the culture medium was aspirated, and 100 μL of DMSO was added to each well to dissolve the formazan crystals, followed by incubation at room temperature for 15 min with gentle shaking. The absorbance was measured at a wavelength of 570 nm using a microplate reader (BioTek, Winooski, VT, USA). Cell viability was calculated using the following formula: Cell viability (%) = (assay value − blank value)/mean value of the control group × 100%.

### 2.6. Griess Assay

Cells were seeded in 12-well plates at a density of 2 × 10^5^ cells per well and incubated overnight. Subsequently, cells were pretreated with LCS2 (10, 20, 40 μg/mL), LCS3 (2, 4, 8 μg/mL), or LCS4 (0.2, 0.4, 0.8 μg/mL) for 2 h, followed by co-incubation with LPS (1 μg/mL) for 18 h [39]. Cell culture supernatants were collected, and nitrite accumulation, an indicator of NO production, was measured using a Griess reagent kit (Cat. No. G9440, Solarbio Science & Technology Co., Ltd., Beijing, China) according to the manufacturer’s instructions.

### 2.7. Compound Identification of LCS2

An appropriate amount of LCS2 powder was accurately weighed, dissolved in methanol to a suitable concentration, and filtered through a 0.22 μm microporous membrane. The chemical constituents in the filtrate were then analyzed using ultra-performance liquid chromatography coupled with quadrupole time-of-flight tandem mass spectrometry (UPLC-Q-TOF-MS/MS) [31,34,40]. Analyses were performed on an Agilent 1290 UPLC system coupled with an Agilent 6545 Q-TOF mass spectrometer (Agilent Technologies, Santa Clara, CA, USA).

Chromatographic conditions: Chromatographic separation was performed on a Welch C18 column (100 mm × 2.1 mm, 1.7 μm) with a flow rate of 0.3 mL/min, column temperature of 45 °C, and injection volume of 2 μL. The mobile phase consisted of 0.1% formic acid aqueous solution (phase A) and acetonitrile (phase B). The gradient elution program was set as follows: 0–0.1 min, 15% B; 0.1–12 min, 15–30% B; 12–20 min, 30–55% B; 20–24 min, 55–95% B; 24–27 min, 95% B; 27–27.1 min, 95–10% B; 27.1–30 min, 10% B (re-equilibration).

Mass spectrometry conditions: Mass spectrometric detection was performed in negative electrospray ionization (ESI^−^) mode, with a mass scan range of *m*/*z* 50–1250. The key MS parameters were set as follows: spray voltage, −4500 V; nebulizer gas temperature, 350 °C; drying gas flow, 10 L/min at 35 psi; nebulizer gas and auxiliary gas pressure, 55 psi; Fragmentor, −100 V. Data were acquired using TOF-MS full scan combined with information-dependent acquisition (IDA) triggered MS/MS scan (TOF-MS-IDA-MS/MS) mode. The IDA settings included multiple mass defect filter (MMDF), multiple neutral loss filter (MNLF), and dynamic background subtraction (DBS) functions.

Data processing was performed using Agilent MassHunter Qualitative Analysis software (v10.0). Compounds were tentatively identified by comparing their accurate mass, MS/MS fragmentation patterns, and retention times with the METLIN database, HMDB database, and the published literature. The maximum allowable mass error was set at ±10 ppm. This threshold was determined based on the analytical performance of the Q-TOF system under the current chromatographic and MS conditions, the complexity of the polyphenol- and tannin-rich plant matrix, and the widely accepted 5–10 ppm range for tentative phytochemical identification in natural product studies without authentic reference standards [41]. The full UPLC-Q-TOF-MS/MS spectra together with fragment assignment data of LCS2 are provided as Supplementary Materials. Although a 5 ppm error is ideal for high-resolution Q-TOF instruments, a 10 ppm window was applied to reduce false-negative identification of low-abundance minor components [42,43]. All compound assignments were further validated by matching characteristic MS/MS fragmentation patterns against the published peer-reviewed literature, which ensures the reliability of identification despite the slightly wider mass tolerance.

### 2.8. Flow Cytometry

Cells were seeded in 12-well plates and incubated overnight. Then, cells were pretreated with LCS2 (40 μg/mL) for 2 h, followed by co-incubation with LPS (1 μg/mL) for 8 h to induce ROS production. 2′,7′-Dichlorodihydrofluorescein diacetate (DCFH_2_-DA, 10 μM) was added to the culture medium, and cells were incubated for 30 min in the dark. After incubation, the supernatant was discarded, and cells were washed twice with pre-cooled phosphate-buffered saline (PBS). Then, 100 μL of trypsin was added to each well for 1 min of digestion, which was terminated by the addition of PBS containing 10% FBS. Cell suspensions were collected into centrifuge tubes, with all operations protected from light. The intracellular ROS level was determined by measuring the fluorescence intensity via flow cytometry.

### 2.9. Immunofluorescence Assay

Cells were seeded in laser confocal culture dishes and incubated overnight. After 2 h of pretreatment with LCS2 (40 μg/mL), cells were co-incubated with LPS (1 μg/mL) for 8 h to induce NF-κB p65 nuclear translocation. Cells were washed twice with PBS and fixed with 4% paraformaldehyde (PFA) at room temperature for 30 min. After fixation, cells were permeabilized with 0.5% Triton X-100 for 15 min and blocked with 5% bovine serum albumin (BSA) for 30 min at room temperature, followed by incubation with anti-NF-κB p65 primary antibody (1:400) at 4 °C overnight. The next day, cells were incubated with Alexa Fluor 488-conjugated goat anti-rabbit IgG secondary antibody (1:500) for 2 h at room temperature in the dark. Nuclei were counterstained with Hoechst 33342, and immunofluorescence images were captured using a laser confocal scanning microscope.

### 2.10. RNA Extraction and Reverse Transcription-Quantitative Polymerase Chain Reaction (RT-qPCR)

RAW264.7 cells were seeded in 6-well plates and cultured for 24 h. Subsequently, cells were pretreated with LCS2 (40 μg/mL) for 2 h, followed by stimulation with LPS (1 μg/mL) for 8 h. Total cellular RNA was extracted using TRIzol reagent according to the manufacturer’s protocol. The extracted total RNA was then reverse-transcribed into complementary DNA (cDNA) using the SweScript RT I First Strand cDNA Synthesis Kit.

Finally, the mRNA expression levels of TNF-α, IL-1β, and IL-6 were quantified via SYBR Green-based real-time quantitative polymerase chain reaction (RT-qPCR). RT-qPCR was performed using UltraSYBR^®^ Mixture (Beijing ComWin Biotech Co., Ltd., Beijing, China) on a CFX96 Real-Time PCR Detection System (Bio-Rad Laboratories, Inc.) following the manufacturer’s instructions. The amplification program was set as follows: initial denaturation at 95 °C for 10 min; followed by 40 cycles of denaturation at 95 °C for 15 s and annealing/extension at 60 °C for 1 min.

After the amplification reaction, the mean threshold cycle (Ct) value was calculated for each sample. GAPDH was used as the endogenous reference gene for normalization, and the relative expression levels of target genes were calculated using the 2^−ΔΔC^^q^ method [44]. All experiments were performed in three independent replicates, with each sample tested in triplicate.

### 2.11. Western Blotting

For Western blotting analysis of inflammatory signaling pathways in RAW264.7 cells, cells were seeded in 6-well plates at a density of 4 × 10^5^ cells per well and cultured overnight. After 2 h of pretreatment with LCS2 (10, 20, 40 μg/mL), cells were stimulated with LPS (1 μg/mL) for 8 h to induce the phosphorylation of IKKα/β, IκBα, p65, JNK, ERK, and p38, as well as the protein expression of COX-2 and iNOS. After treatment, cells were washed twice with pre-chilled PBS and lysed on ice with RIPA lysis buffer (Beyotime Biotechnology, Shanghai, China) supplemented with 1% protease and phosphatase inhibitor cocktails. Cell lysates were scraped, collected into 1.5 mL microcentrifuge tubes, and centrifuged at 15,000× *g* for 25 min at 4 °C. The supernatants were transferred to fresh tubes and stored at −80 °C until further use.

Prostate tissues were collected from rats 8 h after LPS challenge. Approximately 100 mg of prostate tissue was homogenized in 1 mL of ice-cold RIPA lysis buffer (Beyotime Biotechnology, Shanghai, China) supplemented with 1% protease and phosphatase inhibitor cocktails using a tissue homogenizer. The homogenates were centrifuged at 15,000× *g* for 25 min at 4 °C, and supernatants were transferred to fresh tubes and stored at −80 °C until further use. For Western blot analysis of prostate tissue, three rats were randomly selected from each group (*n* = 3 per group) as independent biological replicates. Each lane on the SDS-PAGE gel represented an individual tissue sample from a single rat; no pooled prostate tissue samples were used for any of the Western blot assays.

Protein concentrations of cell and tissue lysates were determined using a bicinchoninic acid (BCA) protein assay kit (Beyotime Biotechnology, Shanghai, China) according to the manufacturer’s instructions. For electrophoretic separation, equal amounts of protein (40 μg per lane) were loaded onto 8–12% SDS-polyacrylamide gels, with the gel concentration selected according to the molecular weight of the target proteins. After electrophoretic separation, proteins were electrotransferred onto polyvinylidene fluoride (PVDF) membranes (Merck Millipore, Burlington, MA, USA), which were pre-activated with 100% methanol for 1 min and equilibrated in transfer buffer prior to use. Electrotransfer was performed at a constant voltage of 100 V for 1.5 h in an ice bath to ensure low-temperature conditions.

Subsequently, the membranes were blocked with 5% non-fat milk in Tris-buffered saline with Tween 20 (TBST) at room temperature for 1–2 h. After washing three times with TBST, membranes were incubated with primary antibodies (diluted 1:1000 in TBST) at 4 °C overnight. The following primary antibodies were used: COX-2 (Cat. No. 4842), iNOS (Cat. No. 7835), p65 (Cat. No. 8242), phospho-NF-κB p65 (p-p65, Cat. No. 3033), IKKα (Cat. No. 2682), IKKβ (Cat. No. 8943T), phospho-IKKα/β (p-IKKα/β, Cat. No. 2697), IκBα (Cat. No. 4814), phospho-IκBα (p-IκBα, Cat. No. 2859), JNK (Cat. No. 9252T), phospho-JNK (p-JNK, Cat. No. 4668T), ERK (Cat. No. 4695T), phospho-ERK (p-ERK, Cat. No. 4370T), p38 MAPK (Cat. No. 9212), phospho-p38 MAPK (p-p38, Cat. No. 4511), and GAPDH (Cat. No. 2118), all from Cell Signaling Technology (Danvers, MA, USA). Membranes were then washed five times with TBST (10 min each) and incubated with horseradish peroxidase (HRP)-conjugated goat anti-rabbit IgG (H + L) secondary antibody (diluted 1:5000 in TBST) at room temperature for 2 h.

Protein bands were detected using an enhanced chemiluminescence (ECL) substrate kit (Biogot Technology Co., Ltd., Nanjing, China) and visualized using a ChemiDoc XRS+ imaging system (Bio-Rad Laboratories, Inc., Hercules, CA, USA). Band optical density was quantified using ImageJ software (version 1.49, National Institutes of Health, Bethesda, MD, USA). For non-phosphorylated target proteins, relative expression levels were normalized to GAPDH, which served as the internal loading control. For all phosphorylated proteins, relative phosphorylation levels were calculated by normalizing the band intensity of the phosphorylated form to that of the corresponding total protein, eliminating the influence of sample loading variation and total protein abundance on phosphorylation quantification.

### 2.12. Enzyme-Linked Immunosorbent Assay

Blood samples were allowed to clot at room temperature for 30 min and then centrifuged at 3000× *g* for 15 min at 4 °C to separate serum. Approximately 100 mg of prostate tissue was homogenized in 9 volumes (*w*/*v*) of ice-cold PBS supplemented with 1% protease inhibitor cocktail using a tissue homogenizer. The homogenates were centrifuged at 12,000× *g* for 20 min at 4 °C, and the supernatants were collected for subsequent detection.

The concentrations of IL-6 (Cat. No. F3066-A), TNF-α (Cat. No. F3056-A), PGE2 (Cat. No. F2944-A), and PSA (Cat. No. F3343-A) in serum and prostate tissue supernatants were determined using commercial enzyme-linked immunosorbent assay (ELISA) kits (Fankewei, Shanghai, China) following the manufacturer’s protocols.

### 2.13. Animal Experiment

All animal experimental procedures were reviewed and approved by the Institutional Animal Welfare and Ethics Committee of Guangxi University of Chinese Medicine (Approval No. DW20230830-147). Forty-two 8-week-old male Sprague-Dawley (SD) rats weighing 180–220 g were purchased from Vital River Laboratory Animal Technology Co., Ltd. (Foshan, China), with a quality certificate No. SCXK (Guangdong) 2022-0063. All animal experiments were conducted in strict accordance with the National Institutes of Health Guide for the Care and Use of Laboratory Animals and reported following the ARRIVE guidelines. All efforts were made to minimize animal suffering and reduce the number of animals used.

All animals were housed under specific pathogen-free (SPF) conditions at a temperature of 25 ± 1 °C and humidity of 50 ± 5%, with a 12 h light/dark cycle and free access to food and water. Previous studies have confirmed that intraprostatic injection of LPS can reliably induce prostatitis in rats [25,26]. We performed pre-experimental optimization based on modeling dosage and inflammatory biomarker levels, and determined that the optimal LPS dosage for modeling was 100 μg/kg and the optimal sample collection time was 8 h post-injection. Formal experiments were then conducted accordingly. Notably, the intraprostatic LPS injection model established in this study primarily mimics acute inflammatory injury of the prostate. The group size of seven rats per group was selected based on two combined considerations: the commonly adopted sample sizes (6–8 rats per group) in peer-reviewed studies using the same rodent model of LPS-induced acute prostatic inflammation [45] and the 3R principle (Replacement, Reduction, Refinement) for ethical animal experimentation. This sample size was deemed sufficient to detect biologically and statistically meaningful differences between treatment groups while minimizing the number of animals used.

### 2.14. Animal Grouping, Modeling and Gavage Administration

After a 7-day acclimatization period, rats were randomly divided into six groups (*n* = 7 per group): a control group, an LPS model group (100 μg/kg), three LCS2 dietary intervention groups (25, 50, and 100 mg/kg), and a tamsulosin hydrochloride (TH) reference control group (0.023 mg/kg) [46,47].

Prior to the formal experiment, a preliminary 4-week study was performed to support dose selection for the main experiment. A total of 12 healthy 8-week-old male SD rats were randomly assigned to two groups (*n* = 6 per group): a normal saline control group and an LCS2 treatment group (100 mg/kg body weight, administered by gavage once daily for 4 weeks). Serum liver function parameters, including alanine aminotransferase (ALT) and aspartate aminotransferase (AST), and circulating pro-inflammatory cytokines (TNF-α, IL-6) were measured at the end of the 4-week administration. No significant differences in these parameters were observed between the two groups. These preliminary data were used solely to justify the selection of 100 mg/kg as the highest dose in the main study, and do not constitute a fully characterized systematic safety assessment. Based on this safety threshold, we set 25, 50, and 100 mg/kg as the gradient doses for the subsequent in vivo experiment to explore the dose–effect relationship of LCS2 against LPS-induced acute prostatic inflammatory injury.

Tamsulosin hydrochloride (TH) was included as a reference control to calibrate the magnitude of inflammatory improvement and to compare the mechanism differences between clinical pharmaceutical intervention and dietary bioactive ingredient intervention. The rationale for selecting TH as the reference control in this study is that, as an α_1_-adrenergic receptor antagonist, it has been clinically proven to alleviate chronic prostatitis-related symptoms such as pain and dysuria, although its primary mechanism is not direct anti-inflammatory action. Previous studies have demonstrated that α-receptor blockers (including TH) can reduce prostatitis symptom scores, as evidenced by decreased total scores on the National Institutes of Health Chronic Prostatitis Symptom Index (NIH-CPSI) [48].

Rats in the dietary pretreatment groups received daily intragastric administration of LCS2 (dissolved in normal saline) or TH, while rats in the control group and LPS group received an equal volume of normal saline via gavage for 7 consecutive days under the same conditions.

On day 7, 1 h after the last administration, rats in the LPS, LCS2, and TH groups received bilateral intraprostatic injection of LPS (100 μL total; 100 μg/kg), with 50 μL injected into each of the dorsal and ventral prostate lobes. Rats in the control group received an equal volume of vehicle (100 μL of normal saline) via the same injection procedure.

A standard intraprostatic injection procedure was followed: the needle tip was inserted 2–3 mm beneath the prostatic capsule and held in place for approximately 20 s to prevent leakage of the injected solution. All intraprostatic injections were performed under deep anesthesia induced by 3% isoflurane inhalation, and rats were placed on a temperature-controlled heating pad throughout the laparotomy procedure. After injection, the abdominal muscles and skin were sutured layer by layer. Postoperative analgesia was provided via a single subcutaneous injection of buprenorphine (0.1 mg/kg).

Throughout the experiment, all rats had free access to food and water. Eight hours after LPS induction, rats were anesthetized with isoflurane, and blood samples were collected for routine blood tests and inflammatory factor detection. After euthanasia, prostate tissues were harvested for subsequent experimental analyses.

### 2.15. Hematoxylin and Eosin Staining

Portions of prostate tissue were fixed in 4% paraformaldehyde solution and processed for hematoxylin and eosin (H&E) staining to observe histopathological changes. H&E staining was performed on paraffin-embedded prostate sections to assess the severity of inflammatory injury.

The severity of inflammation was evaluated using a 0–4 inflammatory scoring system as previously described [19]: score 0, normal prostate tissue with no inflammatory infiltration; score 1, mild inflammation with focal perivascular monocyte aggregation; score 2, mild perivascular monocyte infiltration; score 3, marked perivascular monocyte infiltration with hemorrhage and increased inflammatory cell infiltration in the stroma; score 4, severe perivascular monocyte infiltration with hemorrhage and extensive infiltration of monocytes and macrophages in the interstitial matrix [49].

According to this scoring system, multiple random sections from each prostate sample were scored in a double-blind manner. The final inflammatory score for each sample was calculated as the total score divided by the number of sections evaluated.

### 2.16. Immunohistochemistry Staining

Immunohistochemistry (IHC) staining was performed for PGE2 (1:500), p-p65 (1:200), TNF-α (1:300), and IL-6 (1:500). Briefly, paraffin-embedded prostate sections were deparaffinized, rehydrated, and subjected to antigen retrieval by heating sections in 0.01 M citrate buffer (pH 6.0) at 95 °C for 20 min. Endogenous peroxidase activity was blocked with 3% hydrogen peroxide for 15 min at room temperature, and non-specific binding was blocked with 5% BSA. Sections were then incubated with primary antibodies overnight at 4 °C, followed by incubation with HRP-conjugated secondary antibody for 2 h at 25 °C.

Immunoreactive signals were visualized with 3,3′-diaminobenzidine (DAB) substrate for 3–5 min under microscopic control, and the reaction was terminated with distilled water. Nuclei were counterstained with hematoxylin. Sections were then examined and photographed under a light microscope. IHC staining was semi-quantified using ImageJ software (version 1.49). For each prostate sample, five non-overlapping representative fields of view (200× magnification) were randomly selected.

The Color Deconvolution plugin was used to separate DAB (brown) and hematoxylin (blue) signals. A consistent color threshold was applied across all images to define the positive staining area of the target protein. The integrated optical density (IntDen) of the DAB signal within the positive area was measured. The relative protein expression level was expressed as mean optical density (MOD), calculated as IntDen/total positive area. For each individual rat, the MOD values from the five non-overlapping fields were first averaged to generate one single biological value per animal, and these individual animal-based values were subjected to group-level statistical analysis. Individual microscopic fields were not treated as independent biological replicates. All procedures of field selection and image quantification were performed by two independent investigators blinded to the treatment group assignment to minimize observational bias.

### 2.17. Statistical Analysis

Data analysis was performed using GraphPad Prism 8.0.2 software (GraphPad Software, Inc., La Jolla, CA, USA). All data are presented as mean ± standard deviation (mean ± SD). For all in vitro cell culture experiments (cell viability assay, Griess assay, flow cytometry, RT-qPCR, and cellular Western blotting), *n* = 3 represents three independent biological replicate experiments conducted on separate days, with each experiment performed with technical triplicates. For in vivo experiments including histopathological scoring, ELISA, and immunohistochemistry (IHC), *n* = 7 represents seven individual rats per group (independent in vivo biological replicates). For Western blotting of prostate tissue samples, *n* = 3 represents three individual rats randomly selected from each group of seven animals; each lane on the gel corresponds to one independent tissue sample from a single rat, and no pooled tissue samples were used. Normality of data distribution was assessed using the Shapiro–Wilk test, and homogeneity of variances was tested using Levene’s test. If homogeneity of variance was not met, Welch’s ANOVA followed by Dunnett’s T3 post hoc test was applied. If variances were homogeneous, differences among multiple groups were evaluated using one-way ANOVA followed by Tukey’s post hoc test for pairwise comparisons. A two-tailed *p*-value < 0.05 was considered statistically significant.

## 3. Results

### 3.1. Screening of Safe Concentrations and Anti-Inflammatory Activity of Different Litchi Seed Fractions in RAW264.7 Macrophages

**Figure 1:**
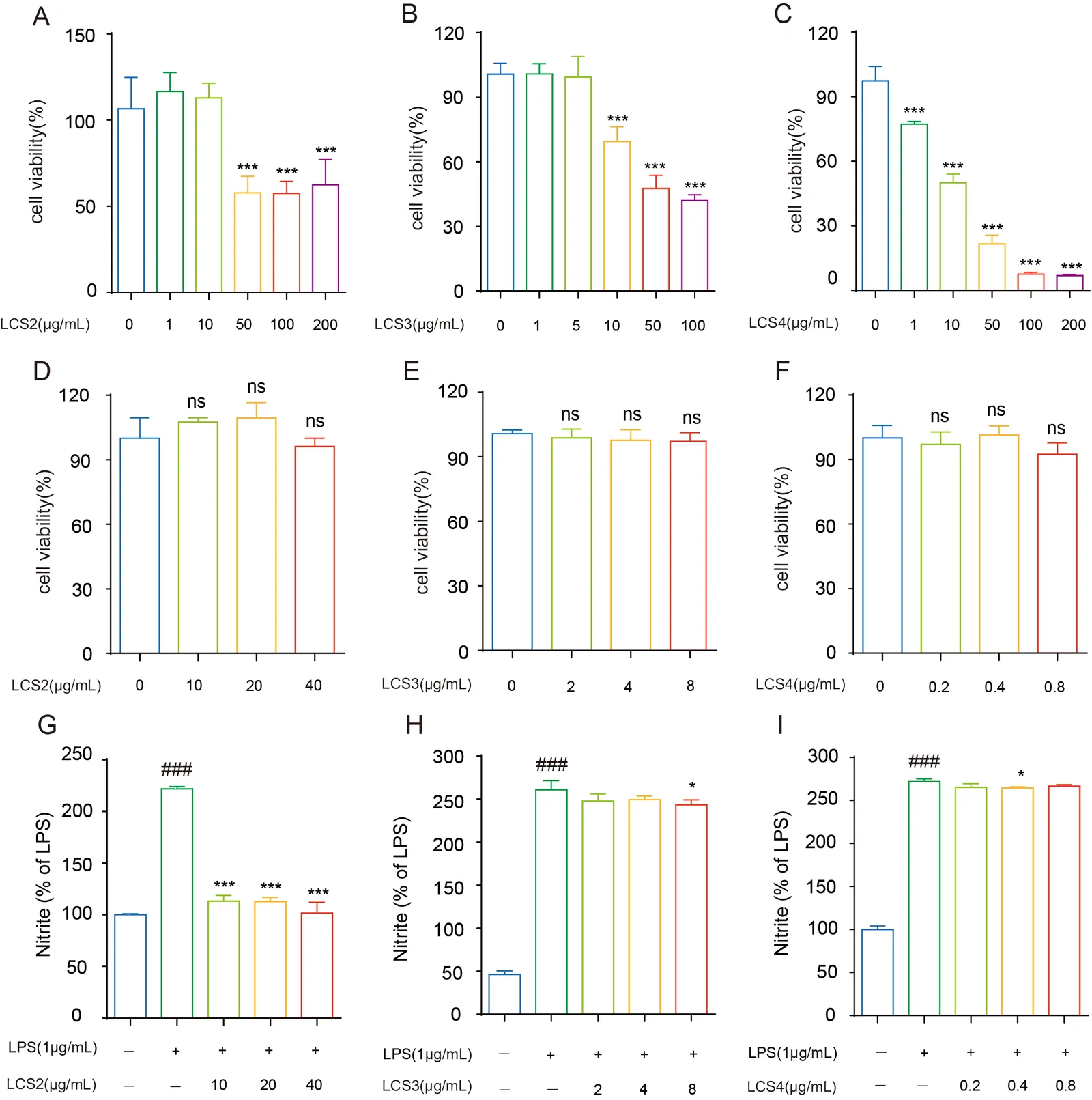
Screening of anti-inflammatory active fractions from litchi seed extract and determination of non-cytotoxic concentrations in RAW264.7 macrophages. (**A**–**C**) Cytotoxicity of graded concentrations of LCS2, LCS3, and LCS4 on RAW264.7 cells was evaluated by MTT assay to define the non-toxic dose ranges. (**D**–**F**) Verification of cell viability at the selected safe concentrations of LCS2, LCS3, and LCS4 by MTT assay. (**G**–**I**) Effects of LCS2, LCS3, and LCS4 on LPS-induced NO production were measured by Griess assay. All data were expressed as mean ± SD (*n* = 3 independent cell culture experiments). ns represents not significant. ^###^ *p* < 0.001 vs. control group; * *p* < 0.05, *** *p* < 0.001 vs. LPS group.

The cytotoxic effects of LCS2, LCS3, and LCS4 on RAW264.7 cells were evaluated via MTT assay, with a broad dose range initially screened to determine the non-cytotoxic dose ranges of the three fractions. The results are shown in Figure 1A–C. After 24 h of treatment, LCS2 at concentrations ≥ 50 μg/mL, LCS3 at ≥ 10 μg/mL, and LCS4 at ≥ 1 μg/mL significantly reduced RAW264.7 cell viability compared with the control group. In contrast, treatment with LCS2 (<50 μg/mL), LCS3 (<10 μg/mL), or LCS4 (<1 μg/mL) for 24 h did not significantly reduce cell viability, indicating no obvious cytotoxicity within these concentration ranges.

Accordingly, LCS2 (10, 20, 40 μg/mL), LCS3 (2, 4, 8 μg/mL), and LCS4 (0.2, 0.4, 0.8 μg/mL) were selected as safe concentrations for subsequent experiments. No significant difference in cell viability was observed between these dietary intervention groups and the control group (Figure 1D–F).

Subsequently, the anti-inflammatory activities of LCS2, LCS3, and LCS4 were evaluated via Griess assay. RAW264.7 cells were pretreated with the above-screened safe concentrations of the three fractions for 2 h, followed by LPS stimulation for 18 h to induce inflammatory responses. As shown in Figure 1G–I, LCS2 (10, 20, 40 μg/mL) dose-dependently reduced nitrite accumulation in the cell supernatant, indicating significant anti-inflammatory activity. In contrast, LCS3 (2, 4, 8 μg/mL) and LCS4 (0.2, 0.4, 0.8 μg/mL) did not significantly reduce nitrite levels under their respective tested conditions. Notably, the three fractions were evaluated at disparate dose ranges determined by their individual cytotoxicity thresholds. This result only demonstrates that LCS2 exerts more favorable anti-inflammatory activity within its non-cytotoxic concentration window and cannot be equated to a direct potency comparison at equivalent concentrations.

### 3.2. Compounds Tentatively Identified in LCS2

**Figure 2:**
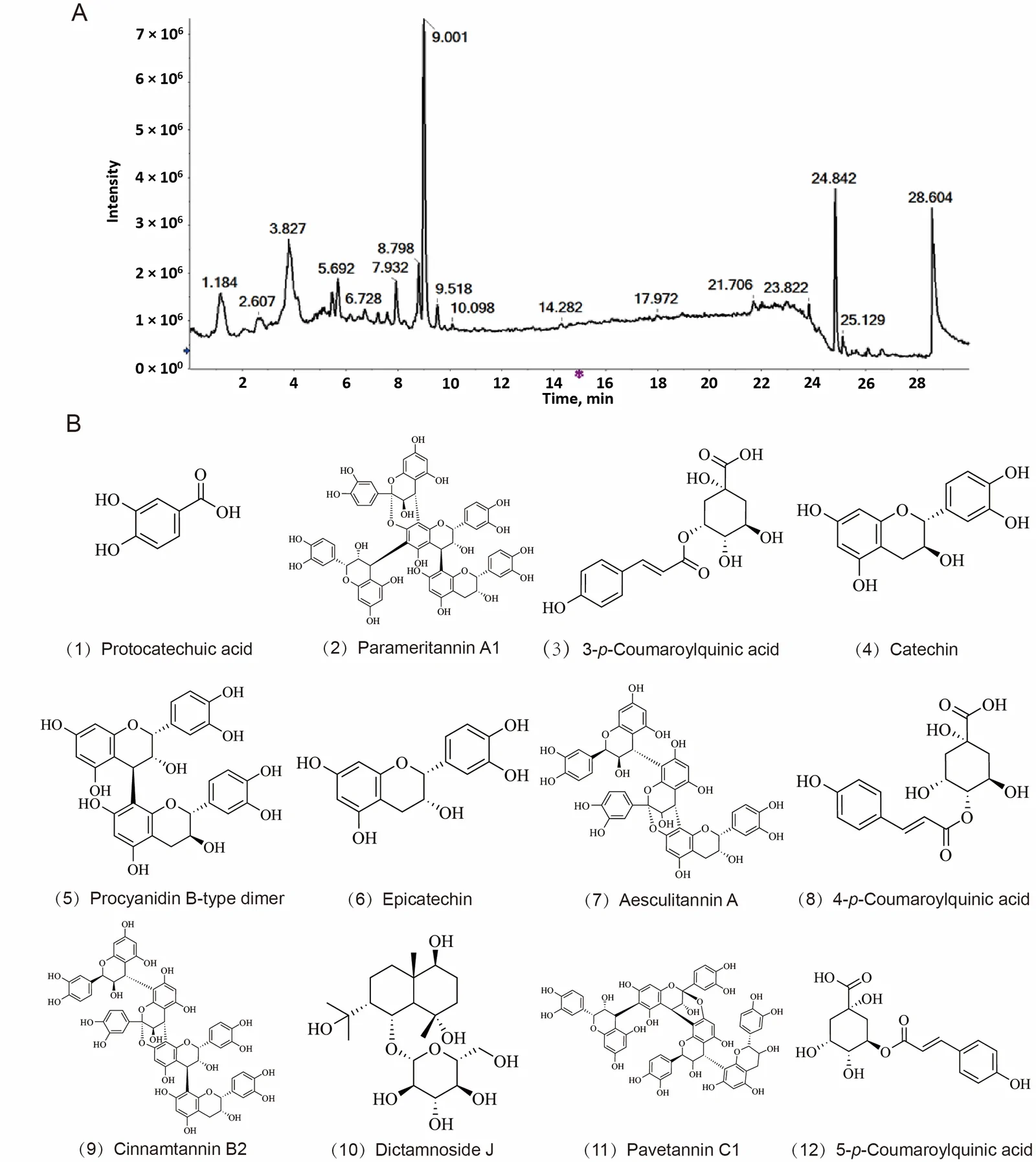

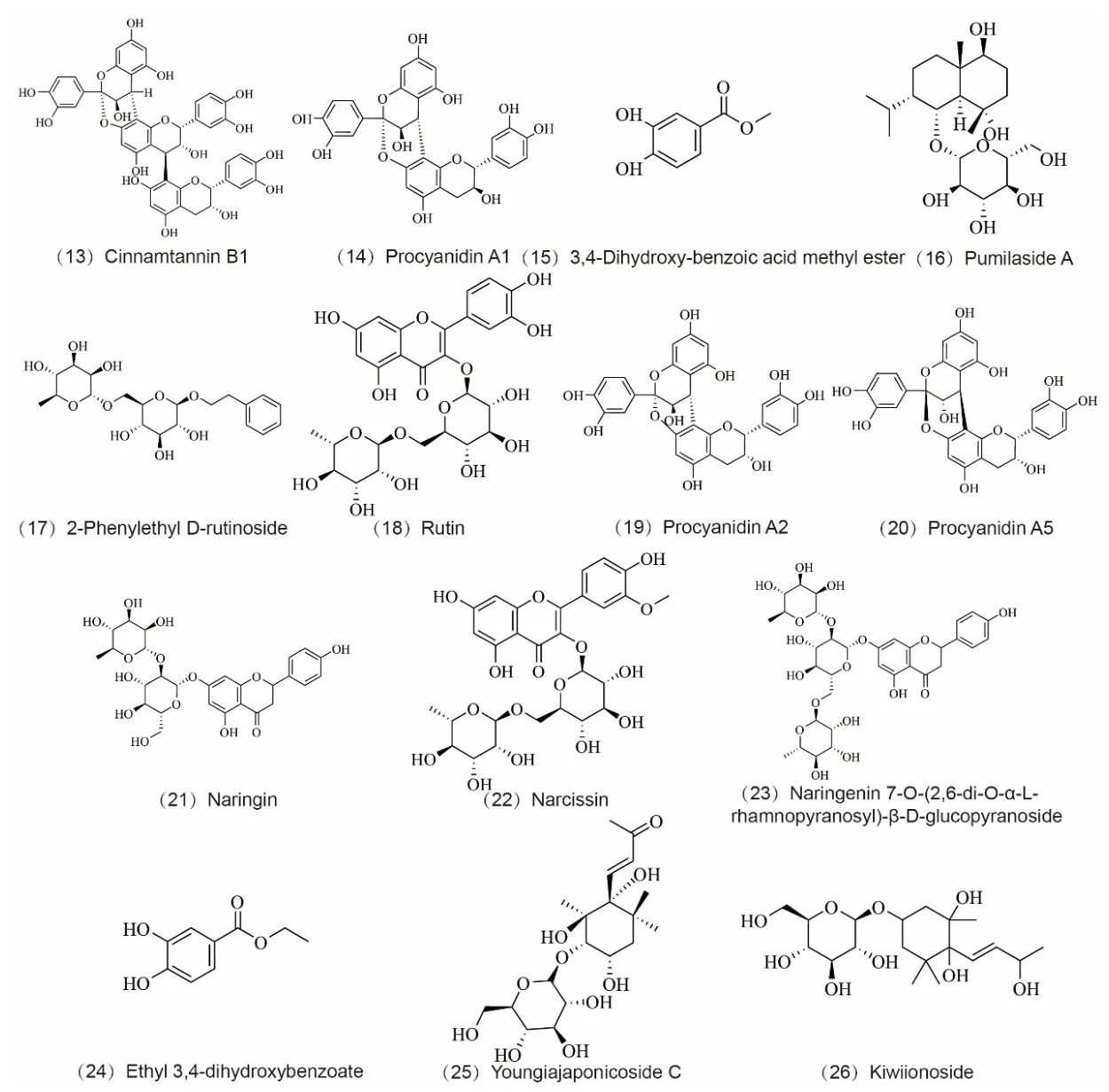
Chemical profiling of the active fraction LCS2 by UPLC-Q-TOF-MS/MS. (**A**) Total ion chromatogram of LCS2 acquired in negative ion mode. (**B**) Representative chemical structures of compounds identified in LCS2.

UPLC-Q-TOF-MS/MS was employed to analyze the chemical composition of LCS2, and the total ion chromatogram (TIC) of LCS2 acquired in negative ion mode is shown in Figure 2A. The relative molecular masses of components with high mass spectral response were identified via data processing, and their structures were deduced by comparing them with natural product databases and analyzing secondary fragment ion patterns.

A total of 26 compounds were tentatively identified in LCS2, including phenolic acids, flavonoids, procyanidins, and tannins, with representative chemical structures shown in Figure 2B. The identification of all compounds was based on accurate mass measurement (mass error < 10 ppm) and MS/MS fragmentation pattern matching with the METLIN database, HMDB database and the published phytochemical literature; no authentic reference standards were used for definitive structural confirmation. The confidence level of each identification is categorized into three grades (High, Medium, Low) and detailed in Table 1. Notably, for procyanidin B-type dimer (peak 5), only the molecular formula and basic skeleton could be confirmed based on accurate mass and characteristic MS/MS fragmentation ions (e.g., *m*/*z* 451, 425, 289). Due to the limited ability of Q-TOF-MS/MS to distinguish interflavan linkage positions and stereochemical configurations, it is not possible to definitively assign this compound to a specific procyanidin B isomer (e.g., B1, B2, B3, B4). Similarly, for oligomeric tannins such as cinnamtannins and parameritannin, the current identification is based on matching of molecular weight and reported fragmentation patterns in the published literature, and absolute stereochemical configuration cannot be fully determined solely by mass spectrometry data. The stereochemical configurations depicted in Figure 2B are drawn based on consensus structures reported in the published literature. Definitive stereochemical assignment for all compounds requires further verification with authentic reference standards. Among the 26 tentatively identified compounds, protocatechuic acid [50,51,52], epicatechin [53], procyanidins [54], and rutin [55,56,57] have been widely reported to exert anti-inflammatory effects by inhibiting NF-κB and MAPK pathways.

### 3.3. LCS2 Pretreatment Inhibits LPS-Induced Production of Inflammatory Mediators and Cytokines in RAW264.7 Cells

We first assessed the cytotoxicity of LCS2 by treating RAW264.7 cells with LCS2 (10, 20, and 40 μg/mL) for 24 h and performing MTT assays. The results confirmed that LCS2 exerted no cytotoxic effects at concentrations below 40 μg/mL (Figure 1D). Therefore, non-cytotoxic concentrations (≤40 μg/mL) were selected for subsequent in vitro anti-inflammatory experiments.

**Figure 3:**
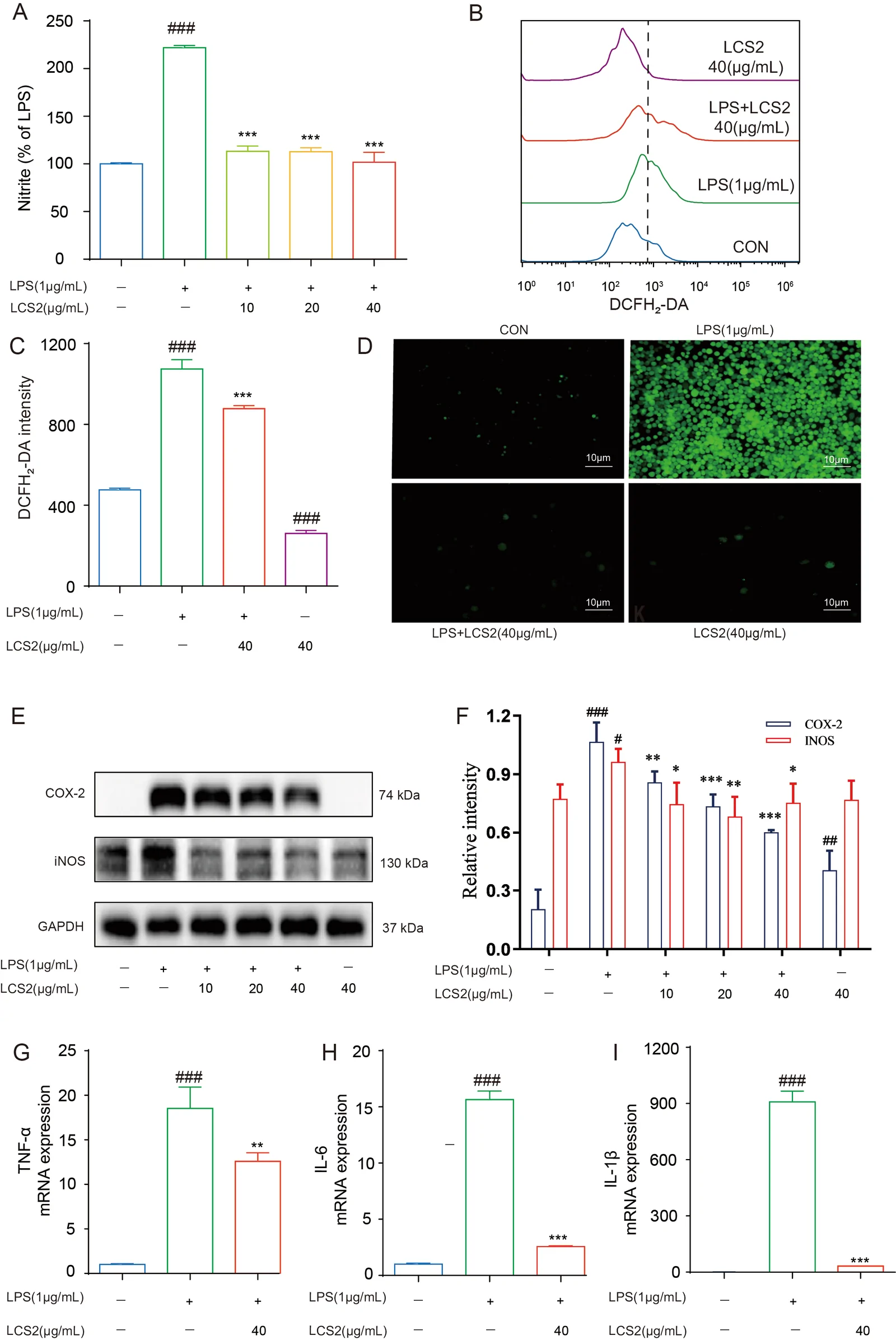
Anti-inflammatory effects of LCS2 on LPS-stimulated RAW264.7 macrophages. (**A**) Effect of LCS2 pretreatment on NO production was detected by Griess assay. (**B**,**C**) Intracellular ROS levels in RAW264.7 cells were measured by flow cytometry. (**D**) Representative fluorescence images of ROS production (×100). (**E**,**F**) Protein expression of COX-2 and iNOS was detected by Western blot analysis. Relative total protein levels were normalized to GAPDH. (**G**–**I**) mRNA expression of TNF-α, IL-6, and IL-1β was detected by RT-qPCR. All data are expressed as mean ± SD (*n* = 3 independent cell culture experiments). ^#^ *p* < 0.05, ^##^ *p* < 0.01, ^###^ *p* < 0.001 vs. control group; * *p* < 0.05, ** *p* < 0.01, *** *p* < 0.001 vs. LPS group.

After 2 h of LCS2 pretreatment, cells were stimulated with LPS (1 μg/mL) for 18 h, and nitric oxide (NO) production was determined by Griess assay. For reactive oxygen species (ROS) measurement, cells were pretreated with LCS2 for 2 h followed by 8 h of LPS stimulation, and then analyzed via flow cytometry. The results showed that LPS stimulation markedly elevated NO production in RAW264.7 cells, whereas LCS2 pretreatment effectively reversed this elevation in a dose-dependent manner (Figure 3A).

Flow cytometry results demonstrated that LPS stimulation markedly elevated intracellular ROS levels, whereas LCS2 pretreatment significantly attenuated LPS-induced ROS overproduction. An LCS2-alone group without LPS stimulation was included to evaluate the effect of LCS2 on basal cellular redox status and to exclude potential pro-oxidative toxicity under physiological resting conditions. Notably, LCS2 treatment alone did not elevate basal ROS levels; instead, it moderately but significantly decreased basal ROS content compared with the control group, indicating that LCS2 has no pro-oxidative effect and possesses intrinsic antioxidant activity under resting conditions (Figure 3B,C). Fluorescence microscopy observation after 8 h of LPS stimulation showed that ROS fluorescence intensity was markedly enhanced in the LPS group, while it was significantly reduced in the LCS2 pretreatment group. The LCS2-alone group exhibited faint ROS fluorescence comparable to or even weaker than the control group, which was consistent with the flow cytometry results (Figure 3D). These results confirmed the successful establishment of the in vitro inflammatory cell model and demonstrated the anti-inflammatory and antioxidant properties of LCS2.

Cyclooxygenase (COX), which consists of the isoenzymes COX-1 and COX-2, plays essential roles in various pathophysiological processes, including inflammation and tumorigenesis [58,59]. Specifically, COX-2 is an inducible isoenzyme that is predominantly expressed in inflamed tissues and is closely associated with the progression of various inflammatory diseases. In the present study, we found that LPS stimulation induced the overexpression of COX-2 and iNOS proteins, which was significantly reversed by LCS2 pretreatment in a dose-dependent manner.

Consistent with the ROS assay results, LCS2 treatment alone did not upregulate the protein expression of COX-2 or iNOS compared with the control group (Figure 3E,F), verifying that LCS2 does not trigger inflammatory activation in resting cells. After 8 h of LPS stimulation, we evaluated the mRNA expression levels of these inflammatory cytokines via RT-qPCR. The results showed that LPS stimulation significantly upregulated the mRNA expression of TNF-α, IL-6, and IL-1β in cells, while LCS2 pretreatment significantly inhibited these upregulations (Figure 3G–I).

These findings suggest that the overexpression of iNOS, COX-2, TNF-α, IL-6, and IL-1β contributes to the inflammatory response in macrophages, and that LCS2 pretreatment effectively alleviates the severity of LPS-induced cellular inflammation, providing in vitro evidence for its anti-inflammatory activity.

### 3.4. LCS2 Pretreatment Attenuated LPS-Induced Cellular Inflammation in Association with Suppression of MAPK and NF-κB Signaling Pathways

**Figure 4:**
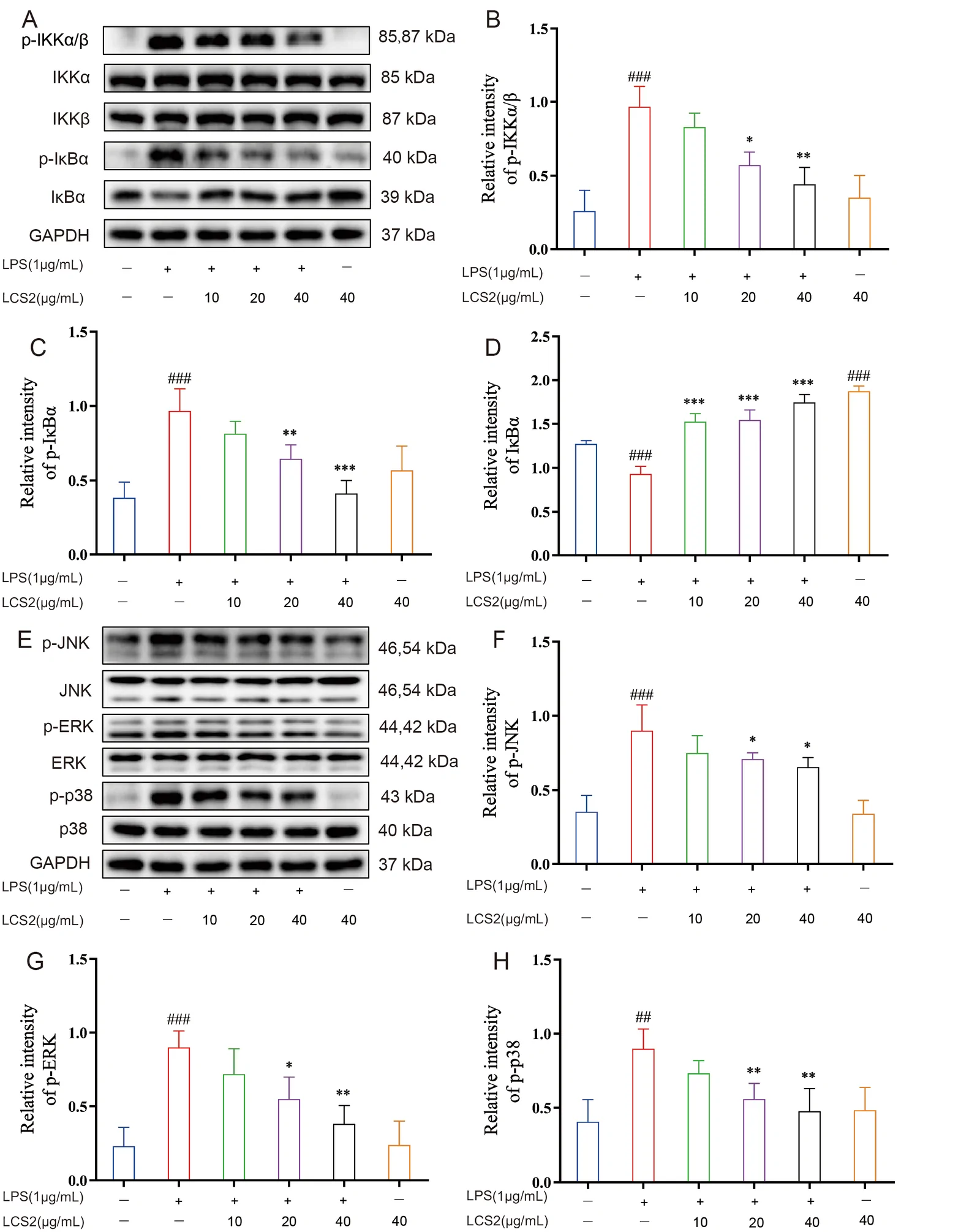

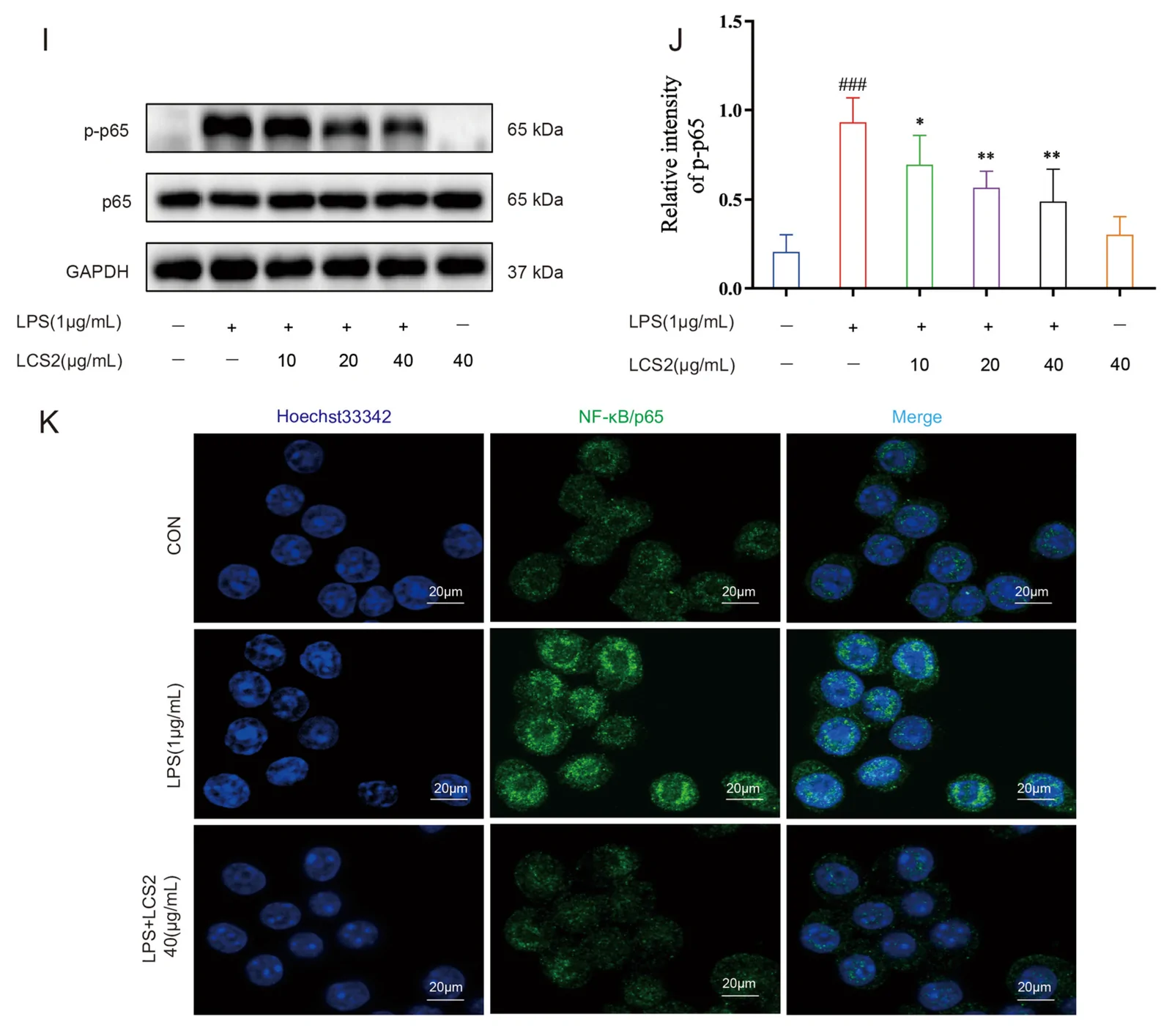
LCS2 pretreatment ameliorates LPS-induced inflammation in RAW264.7 macrophages via MAPK and NF-κB signaling pathways. (**A**–**D**) Protein levels of p-IKKα/β and p-IκBα were assessed by Western blotting. (**E**–**H**) Phosphorylation levels of JNK, ERK, and p38 were analyzed by Western blotting. (**I**,**J**) Protein levels of p-p65 and total p65 were detected by Western blotting. Relative phosphorylation levels were normalized to their corresponding total proteins; relative levels of total proteins were normalized to GAPDH. (**K**) Immunofluorescence assay was performed to observe the nuclear translocation of NF-κB p65 in RAW264.7 cells (×200). All data are expressed as mean ± SD (*n* = 3 independent cell culture experiments). ^##^ *p* < 0.01, ^###^ *p* < 0.001 vs. control group; * *p* < 0.05, ** *p* < 0.01, *** *p* < 0.001 vs. LPS group.

Western blot analysis showed that after 8 h of LPS stimulation in RAW264.7 cells, the phosphorylation levels of IKKα/β and IκBα were significantly increased, while the protein level of IκBα was markedly decreased. In contrast, LCS2 pretreatment significantly reduced the phosphorylation levels of IKKα/β and IκBα, and markedly reversed the LPS-induced degradation of IκBα (Figure 4A–D).

In addition, LCS2 significantly inhibited the phosphorylation of p65 protein without affecting the total p65 protein expression (Figure 4I,J). To further exclude the potential effect of LCS2 on basal pathway activity under resting conditions, an LCS2-alone group without LPS stimulation was included in the experiment. LCS2 treatment alone did not alter the basal phosphorylation levels of IKKα/β, IκBα, and p65, nor did it affect the total protein expression of these molecules compared with the control group (Figure 4A–D,I,J), indicating that LCS2 exerts no regulatory effect on the NF-κB pathway in unstimulated resting macrophages.

This study also investigated the effect of LCS2 on the LPS-induced activation of the MAPK signaling pathway. After 8 h of LPS stimulation, Western blot results showed that the phosphorylation levels of JNK, p38, and ERK were significantly increased. Notably, LCS2 pretreatment dose-dependently reduced the phosphorylation levels of p38, ERK, and JNK (Figure 4E–H). Consistently, no significant difference in the basal phosphorylation of JNK, ERK, and p38 was observed between the LCS2-alone group and the control group (Figure 4E–H), confirming that LCS2 does not interfere with the basal activity of the MAPK signaling pathway under physiological conditions.

Furthermore, the effect of LCS2 on the nuclear translocation of p65 was evaluated via immunofluorescence assay. The results showed that after 8 h of LPS stimulation, the green fluorescence intensity of p65 in the nucleus was significantly increased, indicating that p65 translocated from the cytoplasm into the nucleus. LCS2 pretreatment significantly reduced the nuclear fluorescence intensity of p65, with p65 predominantly distributed in the cytoplasmic region. These observations indicate that although LPS induces the nuclear translocation of p65, LCS2 effectively inhibits this process in macrophages (Figure 4K).

These in vitro data confirm that LCS2 exerts broad anti-inflammatory effects by modulating the NF-κB and MAPK signaling pathways, providing preliminary mechanistic insights for its in vivo prophylactic effect against acute prostatic inflammatory injury.

### 3.5. Prophylactic Administration of LCS2 Attenuates LPS-Induced Pathological Alterations in Rat Prostate Tissue

**Figure 5:**
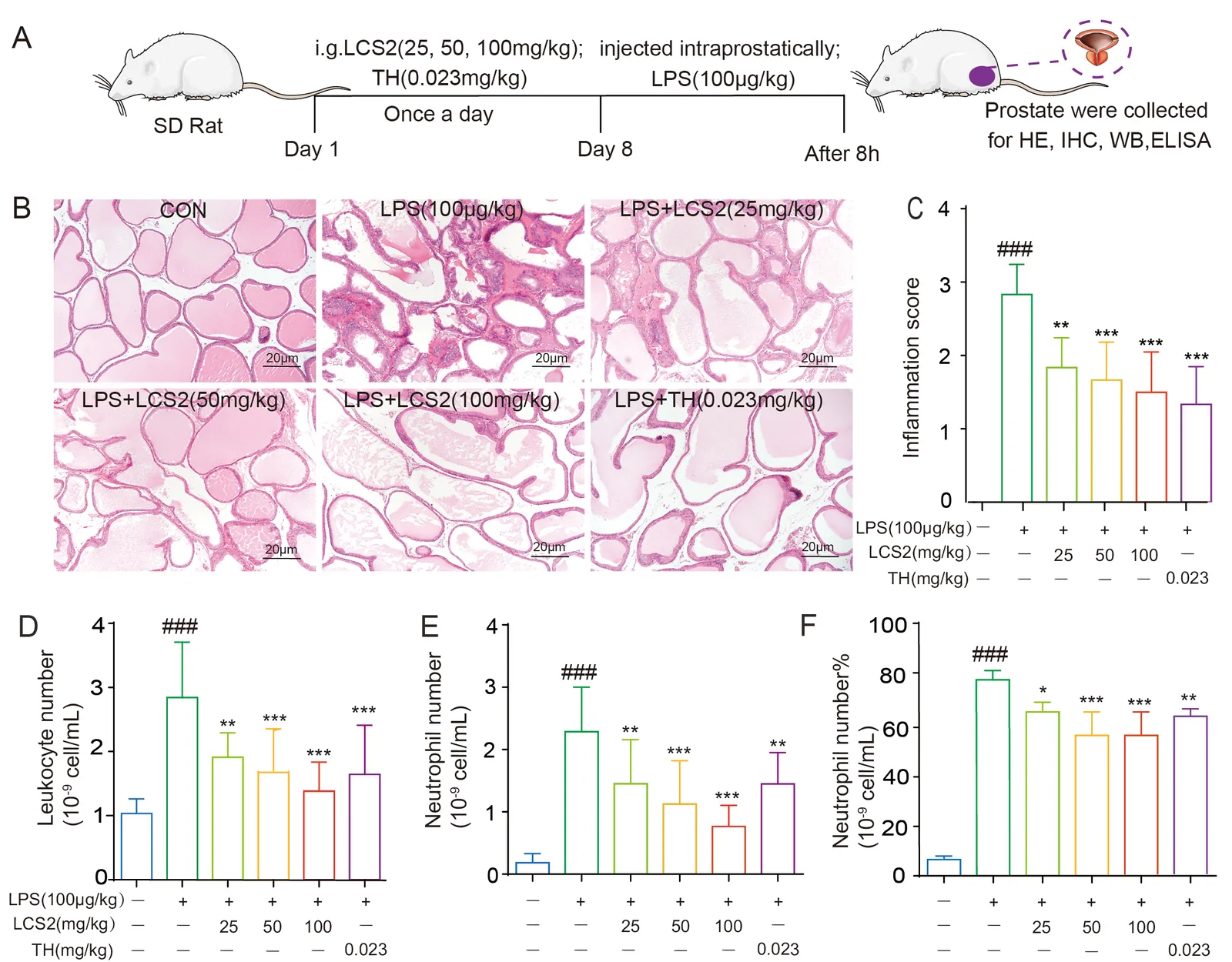
Prophylactic effect of LCS2 on LPS-induced pathological changes in rat prostate tissue. (**A**) Experimental schedule of LCS2 prophylactic administration. (**B**) Representative H&E staining images of prostate tissues (×100). (**C**) Inflammatory scores of rat prostatitis. (**D**–**F**) Changes in white blood cell count, neutrophil count, and neutrophil percentage in rat whole blood were detected by automated hematology analyzer. All data are expressed as mean ± SD (*n* = 7 individual rats per group). ^###^ *p* < 0.001 vs. control group; * *p* < 0.05, ** *p* < 0.01, *** *p* < 0.001 vs. LPS group.

In the present study, the prophylactic potential of LCS2 against LPS-induced acute prostatic inflammatory injury was investigated in a rat model (Figure 5A). In the LPS model group, H&E staining showed disappearance of eosinophilic secretions in the prostatic acinar lumen, disrupted acinar structure, and marked inflammatory cell infiltration in the stroma, confirming successful establishment of the rat acute prostatic inflammatory injury model. In contrast, prophylactic administration of LCS2 or TH significantly reduced the number of neutrophils and other inflammatory cells in the prostatic stroma. Notably, eosinophilic secretions reappeared in the acinar lumen, and the normal morphological structure of prostate tissue was largely restored (Figure 5B,C).

Histopathological analysis further demonstrated that LCS2 pretreatment effectively prevented prostate tissue injury in rats with LPS-induced prostatitis. Routine blood tests showed that white blood cell (WBC) and neutrophil (Neu) counts were significantly increased in the model group, while these increases were significantly suppressed by LCS2 and TH pretreatment (Figure 5D–F). These results indicate that both LCS2 and TH exert significant protective effects against LPS-induced acute prostatic inflammatory injury, which may be attributed to their ability to inhibit pro-inflammatory cytokine release and alleviate inflammatory tissue injury.

### 3.6. Effects of LCS2 Pretreatment on Inflammatory Biomarkers in LPS-Induced Rat Acute Prostatic Inflammatory Injury

**Figure 6:**
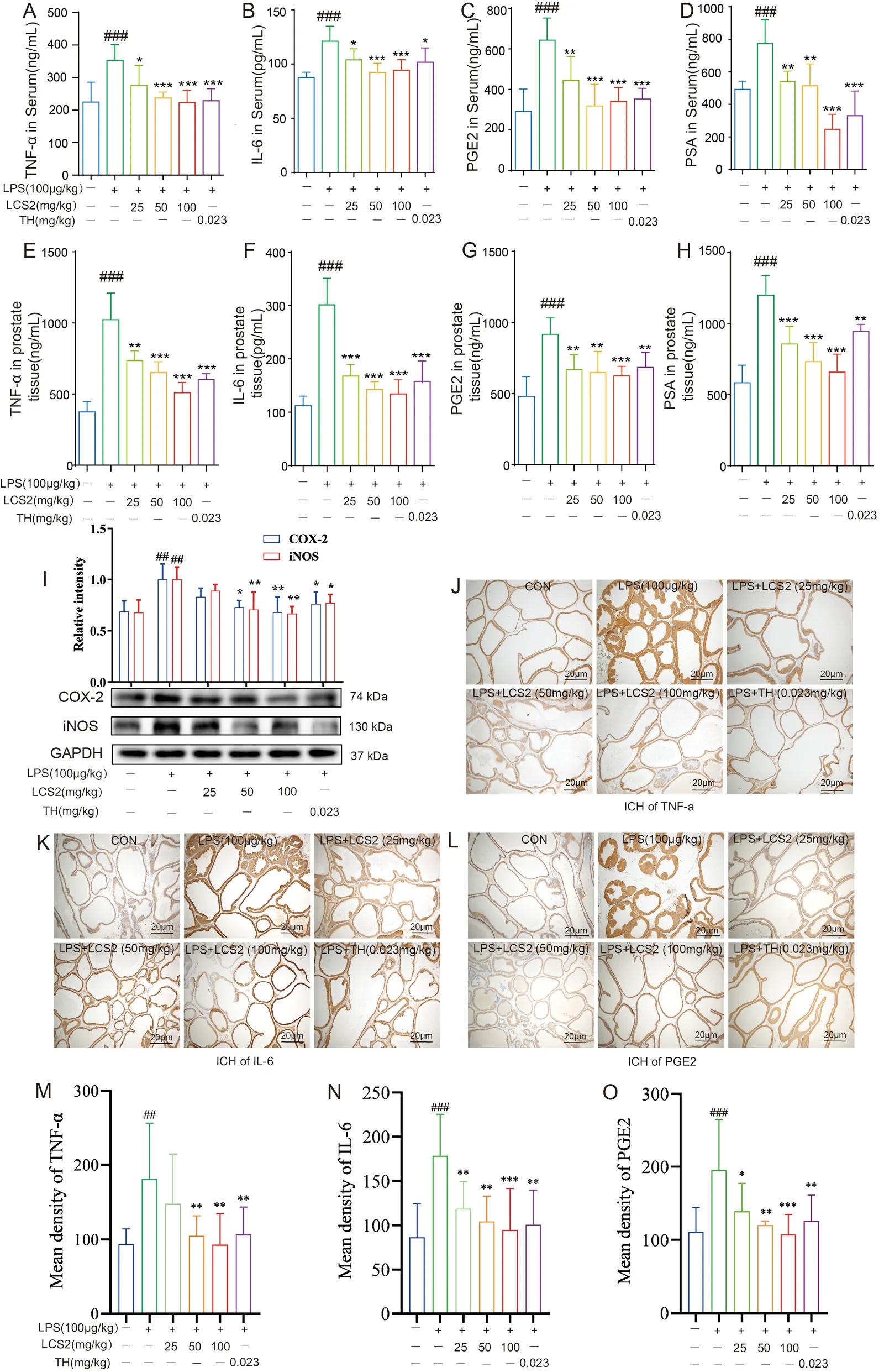
Effects of LCS2 pretreatment on inflammatory biomarkers in LPS-induced rat acute prostatic inflammatory injury. (**A**–**D**) Serum concentrations of TNF-α, IL-6, PGE2, and PSA were determined by ELISA. (**E**–**H**) Concentrations of TNF-α, IL-6, PGE2, and PSA in prostate tissue homogenates were detected by ELISA. (**I**) Protein expression of COX-2 and iNOS in prostate tissue was detected by Western blotting, with GAPDH as the internal reference. (**J**–**O**) Representative immunohistochemical staining images and semi-quantitative analysis of TNF-α (**J**,**M**), IL-6 (**K**,**N**), and PGE2 (**L**,**O**) in prostate sections. Mean optical density (MOD) was calculated to quantify protein expression. Scale bar = 20 μm. All data are expressed as mean ± SD (*n* = 7 individual rats per group for ELISA and IHC; *n* = 3 individual rats per group for WB, randomly selected from each group of 7 animals). ^##^ *p* < 0.01, ^###^ *p* < 0.001 vs. control group, * *p* < 0.05, ** *p* < 0.01; *** *p* < 0.001 vs. LPS group.

ELISA results showed that serum levels of inflammatory cytokines were significantly elevated in LPS-treated rats (Figure 6A–D), indicating successful establishment of the inflammatory model. These elevations were effectively prevented by LCS2 and TH pretreatment. Consistently, LPS induction also significantly increased the levels of TNF-α, IL-6, PGE2, and PSA in prostate tissue homogenates, and these increases were significantly attenuated by LCS2 and TH pretreatment, suggesting that LCS2 inhibits the release of inflammatory mediators in rats with LPS-induced acute prostatic inflammatory injury (Figure 6E–H).

Western blot results showed that LPS upregulated the protein expression of iNOS and COX-2 in prostate tissue, which was significantly reversed by LCS2 and TH pretreatment (Figure 6I). This observation was further supported by IHC results, which showed that LPS induction caused overexpression of TNF-α, IL-6, and PGE2 proteins in prostate tissue, and this overexpression was significantly alleviated by LCS2 and TH pretreatment (Figure 6J–O).

Collectively, these results demonstrate that LCS2 exerts potent protective effects by inhibiting the expression and release of key inflammatory mediators in the serum and prostate tissue of rats with prostatitis. Notably, LCS2 at 100 mg/kg exhibited comparable anti-inflammatory efficacy to the reference control TH.

### 3.7. LCS2 Pretreatment Ameliorated LPS-Induced Acute Prostatic Inflammatory Injury in Rats in Association with Suppression of MAPK and NF-κB Signaling Pathways

Accumulating studies have identified a critical link between NF-κB and MAPK signaling pathways and the pathogenesis of prostatitis. Studies have demonstrated that inflammatory mediators such as TNF-α and ROS induce prostate inflammatory diseases by activating p38 MAPK, ERK, and JNK kinases, which in turn trigger NF-κB phosphorylation [60].

**Figure 7:**
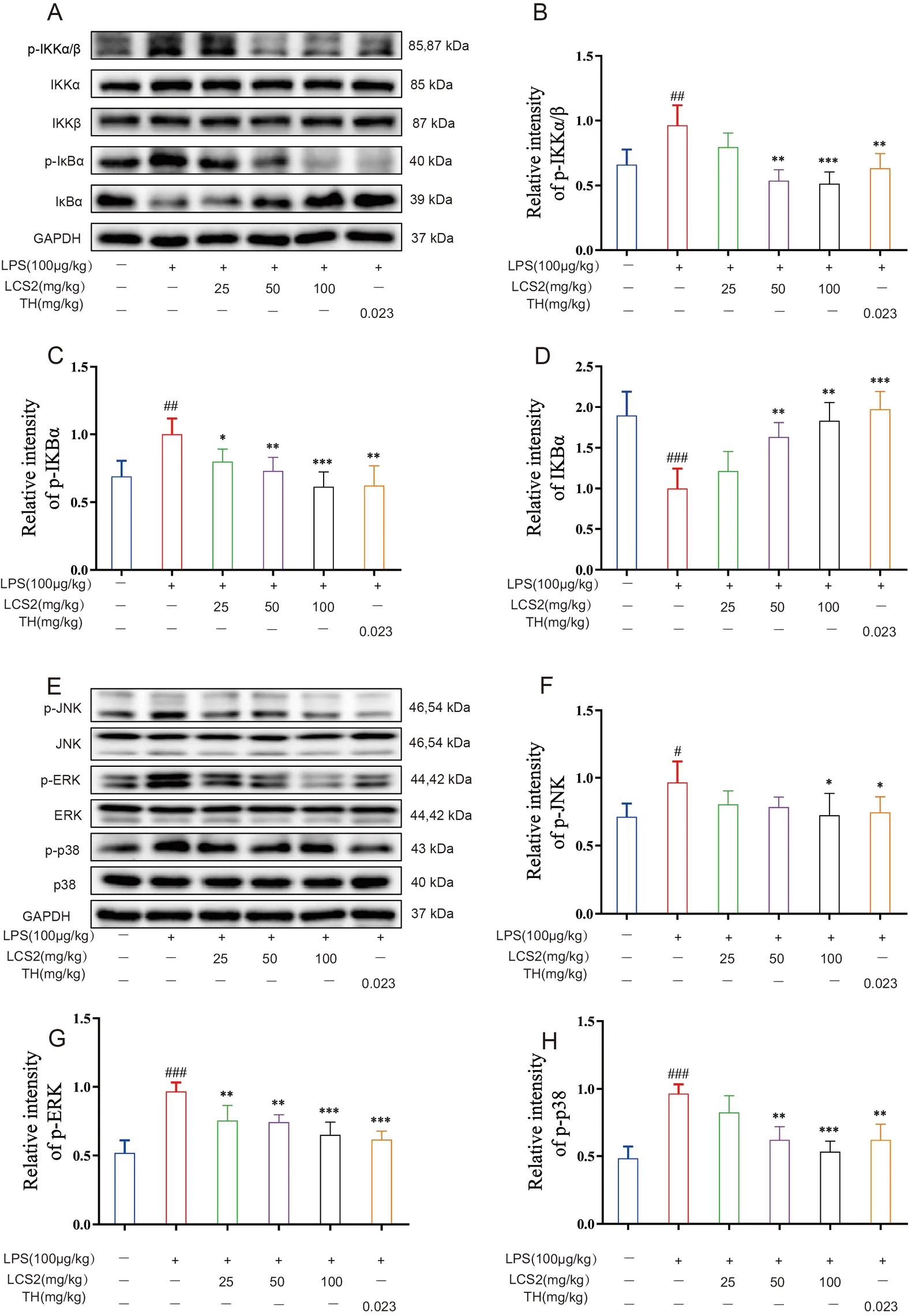

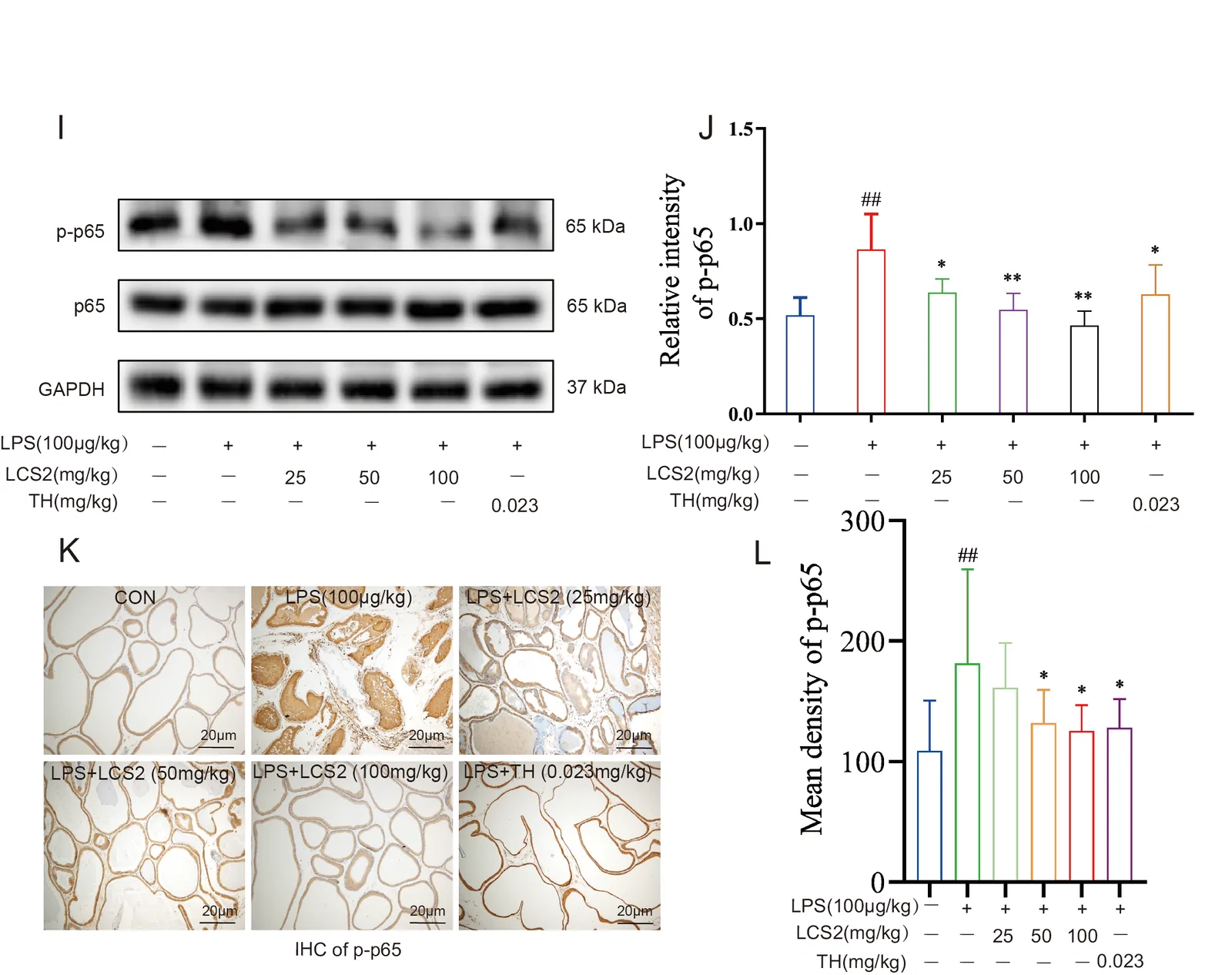
LCS2 pretreatment ameliorates LPS-induced acute prostatic inflammatory injury in rats via MAPK and NF-κB signaling pathways. (**A**–**D**) Protein levels of p-IKKα/β and p-IκBα were measured by Western blotting. (**E**–**H**) Phosphorylation levels of JNK, ERK, and p38 were analyzed by Western blotting. (**I**,**J**) Effect of LCS2 pretreatment on p-p65 and total p65 protein expression was detected by Western blotting. Relative phosphorylation levels were normalized to their corresponding total proteins; relative levels of total proteins were normalized to GAPDH. (**K**,**L**) p-p65 protein level was measured by IHC (×200). All data are expressed as mean ± SD (*n* = 7 individual rats per group for IHC; *n* = 3 individual rats per group for WB, randomly selected from each group of 7 animals). ^#^ *p* < 0.05, ^##^ *p* < 0.01, ^###^ *p* < 0.001 vs. control group; * *p* < 0.05, ** *p* < 0.01, *** *p* < 0.001 vs. LPS group.

Our data showed that in the prostate tissues of LPS-induced rats, the phosphorylation levels of IKKα/β, IκBα, ERK, p38, and JNK were significantly increased, while the protein level of IκBα was significantly decreased. In contrast, pretreatment with LCS2 or TH effectively suppressed the phosphorylation of these proteins and significantly upregulated IκBα protein expression. Notably, neither LCS2 nor TH affected the total protein expression of IKKα, IKKβ, JNK, ERK, and p38 (Figure 7A–H).

Furthermore, LCS2 dose-dependently inhibited the phosphorylation of p65 protein without affecting total p65 protein levels (Figure 7I,J). IHC analysis further confirmed that LPS significantly increased p-p65 expression in prostate tissue, which was significantly attenuated by LCS2 and TH pretreatment (Figure 7K,L).

These results suggest that LPS stimulates the production of inflammatory mediators, which in turn activates the NF-κB and MAPK signaling pathways, thereby exacerbating prostatic inflammation. This inflammatory cascade is effectively inhibited by LCS2 pretreatment. Representative original uncropped Western blot images from both in vitro and in vivo experiments are available in the Supplementary Materials.

## 4. Discussion

### 4.1. Context and Research Gap

The litchi processing industry generates massive amounts of seed by-products annually, while consumer demand for natural dietary ingredients targeting prostate health continues to grow. Against this backdrop, exploring bioactive constituents from litchi seeds carries dual value for agricultural resource valorization and public health promotion. Traditionally, litchi seeds have been used in dietary therapy to manage genitourinary discomfort in China [61], but modern scientific evidence supporting this application, particularly for prostatic inflammatory injury, remains scarce and fragmented.

Over the past decade, research on litchi seed bioactivity has expanded, but two critical gaps remain in the field of prostate health. First, most studies have focused on crude extracts or total flavonoid/polyphenol fractions [31,33,62], with no systematic screening of defined bioactive fractions specifically for prostatic protection. Second, existing anti-inflammatory studies of litchi seed components are largely limited to in vitro cellular models [31,62], and the in vivo efficacy and underlying mechanisms against prostatic inflammatory injury remain largely uncharacterized. To address these gaps, the present study investigated the protective effects of LCS2, a 30% ethanol-eluted polyphenol-rich fraction from litchi seeds, against LPS-induced acute prostatic inflammatory injury, and deciphered its underlying mechanisms.

### 4.2. Novelty of the Present Study Relative to Previous Research

Compared with published studies on litchi seed polyphenols, the present study advances the field in three key aspects.

First, it moves beyond crude extracts to a well-defined bioactive fraction with enriched active constituents. Previous studies mostly used unfractionated crude extracts or total polyphenol/flavonoid mixtures [31,62,63], which vary widely in composition across preparations and hinder reproducibility and quality control. In this study, we obtained LCS2 through gradient elution purification and confirmed it was enriched in flavonoids, procyanidins and tannins via UPLC-Q-TOF-MS/MS. This fractionated approach provides a more chemically defined material basis for subsequent bioactivity research and future functional food development. Notably, among the 26 tentatively identified compounds, protocatechuic acid, epicatechin, procyanidins and rutin have all been individually reported to suppress NF-κB and MAPK signaling [50,51,52,53,54,55,56,57], suggesting that the bioactivity of LCS2 likely arises from the synergistic action of multiple polyphenolic components rather than a single active compound. This is consistent with the consensus that natural product fractions often exert superior effects through multi-component synergism [64].

Second, this study provides the first in vivo evidence that a litchi seed-derived polyphenol fraction protects against acute prostatic inflammatory injury. Prior anti-inflammatory investigations of litchi seed extracts were mostly conducted in RAW264.7 macrophages or other general cellular models, and only described broad inhibitory effects on inflammatory cytokines [31,62]. In contrast, we verified the protective efficacy of LCS2 in a rat model of LPS-induced acute prostatitis, with improvements in both histopathological injury and systemic inflammatory biomarkers. Critically, these in vivo findings demonstrate that bioactive components in LCS2 can reach the prostate tissue and exert local anti-inflammatory effects after oral administration—a conclusion that cannot be inferred from in vitro data alone. This finding fills an important gap between cellular-level activity screening and in vivo prostatic protection.

Third, this study provides a more systematic mechanistic understanding by demonstrating dual inhibition of NF-κB and MAPK signaling pathways at multiple regulatory levels. Previous mechanistic studies of litchi seed extracts were either descriptive of downstream cytokine changes or only examined a single signaling node [31,62]. For example, prior research only showed that total flavonoids from litchi seed inhibit prostate cancer via the NF-κB/AKT/mTOR pathway [63], but did not dissect the multi-level regulation of inflammatory signaling cascades. Here, we systematically verified that LCS2 modulates key nodes at both upstream and downstream levels of both pathways: in the NF-κB cascade, it suppresses IKKα/β phosphorylation, inhibits IκBα degradation, and reduces p65 nuclear translocation; in the MAPK cascade, it dose-dependently decreases phosphorylation of JNK, ERK and p38. The consistent observation of these regulatory effects in both in vitro macrophages and in vivo prostate tissue provides robust mechanistic evidence.

### 4.3. Mechanistic Implications and Comparative Value

From a mechanistic perspective, the dual inhibition of NF-κB and MAPK pathways is particularly meaningful for prostatic inflammation. Accumulating evidence indicates that these two pathways do not function independently; instead, they form a positive feedback loop in the pathogenesis of prostatitis: inflammatory mediators activated by NF-κB can trigger MAPK phosphorylation, and activated MAPK in turn amplifies NF-κB transcriptional activity [65,66]. Therefore, simultaneously targeting both pathways may achieve more comprehensive anti-inflammatory effects than inhibiting a single cascade. This may explain the robust protective effects observed in this study.

It is also noteworthy to compare the mechanism of LCS2 with the reference drug tamsulosin hydrochloride (TH). As an α_1_-adrenergic receptor antagonist, TH alleviates prostatitis symptoms primarily by improving urinary flow and reducing tissue edema, rather than exerting direct anti-inflammatory effects [48]. In this study, LCS2 at 100 mg/kg exhibited comparable improvements in histopathological scores and inflammatory biomarker levels to TH, but acted through a direct anti-inflammatory mechanism targeting inflammatory signaling pathways. This distinction highlights the unique value of LCS2 as a dietary bioactive ingredient: it may serve as an early-stage intervention approach targeting the inflammatory root cause, complementing symptomatic pharmacological treatment in clinical management.

### 4.4. Limitations and Critical Considerations

Despite the promising findings, several limitations must be acknowledged when interpreting the results to ensure a balanced and critical evaluation.

First, regarding experimental model validity, the LPS-induced acute prostatitis model only reflects acute inflammatory injury [67], and the 8-h observation window only captures the early phase of the inflammatory response. The pathogenesis of clinical chronic prostatitis/chronic pelvic pain syndrome (CP/CPPS) is far more complex, involving persistent inflammation, immune dysregulation, stromal remodeling and neuropathic pain. Therefore, the current findings cannot be directly extrapolated to chronic prostatitis conditions, and the long-term efficacy of LCS2 in chronic disease contexts remains to be verified. This limitation restricts the translational relevance of the current findings to acute inflammatory scenarios only.

Second, regarding mechanistic depth, this study only demonstrates an association between LCS2 treatment and inhibition of NF-κB/MAPK pathways, rather than establishing a causal relationship. Pathway-specific inhibition or gene knockdown studies are needed to confirm that these pathways are functionally required for the protective effects of LCS2. In addition, in vitro experiments were performed only in RAW264.7 macrophages, which can only confirm broad-spectrum anti-inflammatory activity but cannot fully recapitulate cell-type-specific responses in prostate tissue. The effects of LCS2 on prostate epithelial cells, stromal cells and other key cell types involved in prostatitis remain unclear, and the crosstalk between NF-κB/MAPK pathways and other inflammatory signaling cascades has not been fully explored.

Third, regarding phytochemical and fraction screening methodology, several caveats exist. Only LCS2 was subjected to detailed chemical profiling, and no parallel comparative analysis of LCS3 and LCS4 at equivalent concentrations was performed. Therefore, the current screening results only support the superior activity of LCS2 within its respective non-cytotoxic dose range, and the relative intrinsic anti-inflammatory potency of the three fractions at equal concentrations remains to be further verified. In addition, LCS1 and LCS5 were excluded solely based on their presumed chemical properties, without experimental verification of their potential bioactivity. Moreover, as LCS2 is a complex multi-component fraction, it remains difficult to attribute the observed effects to specific constituents or to clarify whether the activity arises from individual active compounds or synergistic interactions among multiple components.

Fourth, regarding safety assessment, only a preliminary short-term safety evaluation was conducted, with a lack of systematic long-term toxicity data. This restricts the application potential of LCS2 as a functional food ingredient, as comprehensive safety profiling is a prerequisite for food application [68].

### 4.5. Future Research Directions

Corresponding to the aforementioned limitations, future investigations can be expanded in four directions.

First, expand experimental models and clarify time–effect relationships. Multiple observation time points (e.g., 24 h, 48 h, 72 h, and 7 days) should be included to delineate the time course of the anti-inflammatory effect of LCS2 and verify its potential disease-modifying activity. Clinically relevant chronic prostatitis models should also be established to evaluate the long-term preventive and protective effects of LCS2, so as to improve the translational value of the findings [69].

Second, deepen mechanistic exploration to identify specific targets and cell-type-specific effects. Pathway-specific inhibitors, gene knockdown/overexpression strategies, and nuclear/cytoplasmic fractionation assays should be employed to confirm the causal role of NF-κB and MAPK pathway suppression and to identify the direct molecular targets of LCS2. In addition, the protective effects of LCS2 should be verified in prostate-relevant cell types, including prostate epithelial cells, stromal cells, and CD4^+^ T lymphocytes, to fully elucidate cell-type-specific mechanisms. Multi-omics approaches can also be applied to decipher the complex signaling regulatory network of LCS2.

Third, refine the material basis and elucidate active constituents and synergistic mechanisms. Quantitative analysis of the tentatively identified compounds in LCS2 should be performed, and quality control markers should be established to ensure batch-to-batch consistency. The core active monomeric compounds in LCS2 should be isolated and identified, and their individual activities as well as potential synergistic interactions should be investigated. Furthermore, comparative phytochemical and bioactivity studies of all fractions should be conducted to provide more robust evidence for the selection of LCS2.

Fourth, conduct systematic evaluation of dietary applicability to promote its application in functional foods. Long-term toxicity assessments should be performed to support the development and application of LCS2 as a functional food ingredient for prostate health [68].

## 5. Conclusions

**Figure 8:**
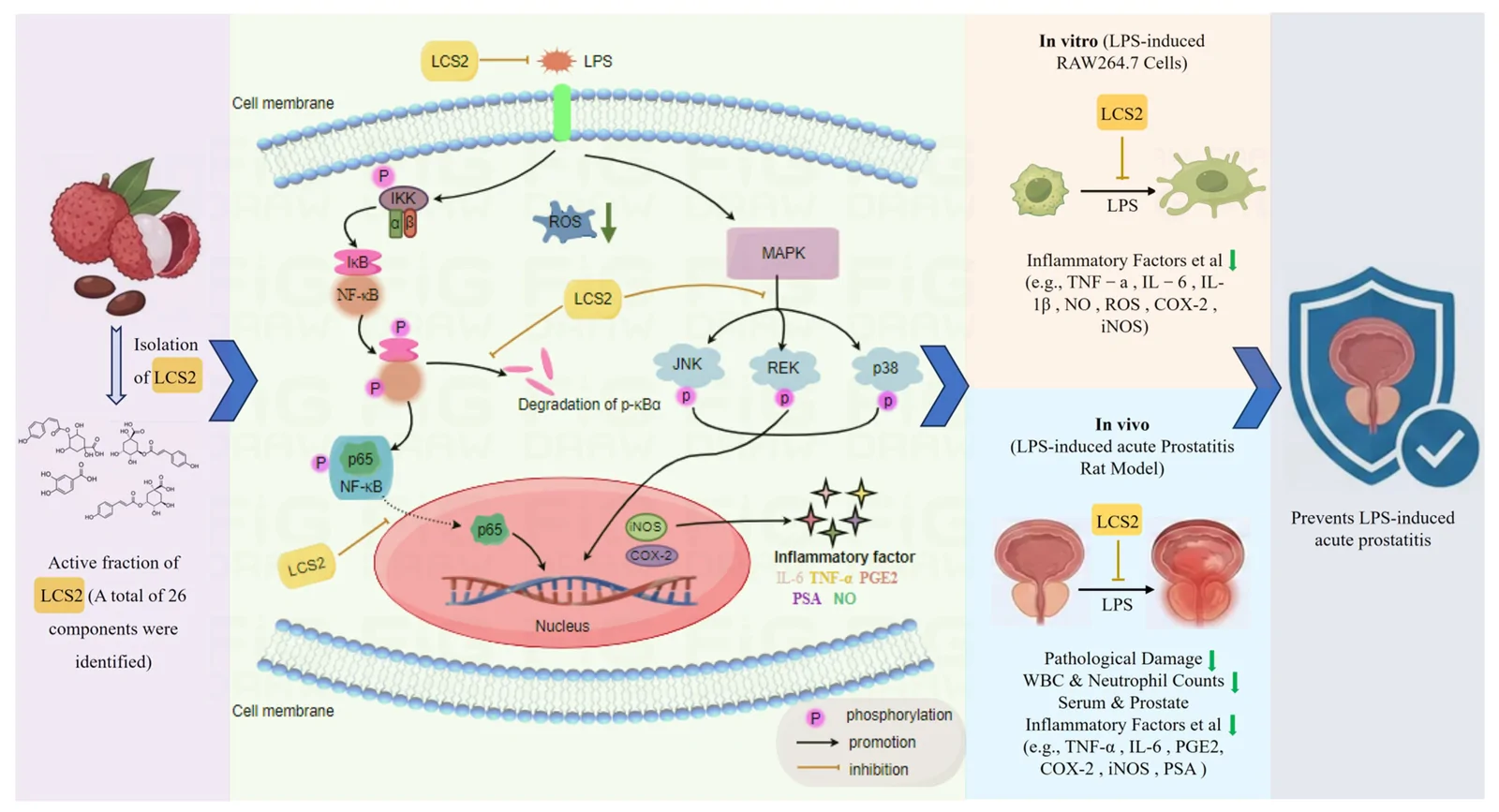
Schematic diagram illustrating the prophylactic mechanism of LCS2 against LPS-induced acute prostatic inflammatory injury, with supporting in vitro validation in LPS-stimulated RAW264.7 macrophages. LCS2 intervention suppresses the activation of both NF-κB and MAPK signaling pathways: in the NF-κB pathway, it reduces the phosphorylation of IKKα/β, IκBα, and p65, and inhibits the nuclear translocation of NF-κB p65; in the MAPK pathway, it decreases the phosphorylation of p38, ERK, and JNK. These regulatory effects consequently reduce the production of pro-inflammatory cytokines (e.g., TNF-α, IL-6, IL-1β), inflammatory mediators (NO, iNOS, COX-2), and reactive oxygen species (ROS), thus exerting a prophylactic effect against LPS-induced acute prostatic inflammatory injury.

This study systematically demonstrates that LCS2, the 30% ethanol-eluted active fraction of litchi seed extract, exerts robust anti-inflammatory activity in LPS-stimulated RAW264.7 macrophages and significant protective effects against LPS-induced acute prostatic inflammatory injury in rats. Chemical characterization via UPLC-Q-TOF-MS/MS tentatively identified 26 compounds in LCS2, predominantly flavonoids, polyphenols, and tannins, which constitute the phytochemical basis of its biological activity. Figure 8 provides a schematic overview of the core protective mechanism of LCS2 against LPS-induced acute prostatic inflammatory injury, as validated by both the in vitro cellular and in vivo animal experiments in this study.

In vitro, LCS2 pretreatment effectively inhibits LPS-induced production of NO, ROS, and pro-inflammatory cytokines, and downregulates the protein expression of iNOS and COX-2. In vivo, 7-day prophylactic administration of LCS2 markedly attenuates acute prostatic pathological injury in the LPS-induced rat prostatitis model, reduces peripheral white blood cell and neutrophil counts, and decreases pro-inflammatory mediator levels in both serum and prostate tissue. Mechanistically, the protective effects of LCS2 are associated with the suppression of NF-κB and MAPK signaling pathway activation, as evidenced by reduced phosphorylation of core signaling molecules and inhibited nuclear translocation of NF-κB p65.

These findings provide scientific evidence for the prostate-protective effect of the LCS2 fraction from litchi seed by-product and highlight the high-value utilization potential of litchi seed as an underutilized agricultural by-product. LCS2 shows preliminary bioactive potential for prostate health-related applications. However, several critical parameters required for functional food development remain unassessed in the current study, including gastrointestinal digestibility, intestinal bioaccessibility, stability during food processing and storage, long-term dietary safety, recommended dietary intake dose, and systemic oral bioavailability [70]. Therefore, its suitability for development as a functional food ingredient requires further systematic validation. Specifically, follow-up studies should include food matrix compatibility evaluation, long-term dietary efficacy and safety assessments, and pharmacokinetic investigations to validate its applicability in real food systems and support its future translational development [71].

## Figures and Tables

**Table 1:** Compounds tentatively identified in LCS2 by UPLC-Q-TOF-MS/MS in negative ion mode. All identifications were based on accurate mass measurement (mass error < 10 ppm) and MS/MS fragmentation patterns compared with published literature and databases. No authentic reference standards were used for definitive structural confirmation. Confidence levels are defined as follows: High: Structure unambiguously assigned; characteristic MS/MS fragments match well with databases and the literature, with no isomeric ambiguity. Medium: Basic skeleton and compound class confirmed; positional or stereoisomers cannot be fully distinguished by MS alone, and the assignment refers to reported retention order and fragmentation patterns in the literature. Low: Only compound class and general skeleton confirmed; specific isomeric configuration cannot be determined by Q-TOF-MS/MS alone, and the structure is tentatively proposed according to the literature.

Peak	tR /min	Formula	[M-H]- (*m*/*z*)	Theoretical Value (*m*/*z*)	Error (ppm)	MS/MS Fragment Ions	Identity	Confidence Level
1	2.14	C_7_H_6_O_4_	153.0193	153.0188	3.3	109.0302, 108.0219, 91.0209	Protocatechuic acid	High
2	2.15	C_60_H_48_O_24_	1151.2525	1151.2463	5.4	999.2039, 573.1041, 287.0575, 125.0244	Parameritannin A1	Low
3	2.66	C_16_H_18_O_8_	337.0931	337.0929	0.6	191.0565, 173.0456, 163.0402	3-*p*-Coumaroylquinic acid	Medium
4	2.75	C_15_H_14_O_6_	289.0720	289.0718	0.7	245.0822, 179.0362, 151.0391, 137.0230, 109.0287	Catechin	High
5	3.11	C_30_H_26_O_12_	577.1369	577.1352	3.0	451.1056, 425.0890, 407.0801, 289.0726	Procyanidin B-type dimer	Medium
6	3.65	C_15_H_14_O_6_	289.0720	289.0718	0.7	245.0822, 179.0362, 151.0391, 137.0230, 109.0287	Epicatechin	High
7	3.80	C_45_H_36_O_18_	863.1865	863.1829	4.2	573.1047, 451.1063, 411.0740, 289.0700	Aesculitannin A	Low
8	3.85	C_16_H_18_O_8_	337.0931	337.0929	0.6	191.0565, 173.0456, 163.0402	4-*p*-Coumaroylquinic acid	Medium
9	4.09	C_60_H_48_O_24_	1151.2525	1151.2463	5.4	999.2039, 573.1041, 287.0575, 125.0244	Cinnamtannin B2	Low
10	4.16	C_21_H_38_O_9_	433.2462	433.2443	4.4	271.1910, 253.1797, 179.0558, 161.0447	Dictamnoside J	Medium
11	4.75	C_60_H_48_O_24_	1151.2525	1151.2463	5.4	999.2039, 573.1056, 287.0575, 125.0258	Pavetannin C1	Low
12	4.88	C_16_H_18_O_8_	337.0931	337.0929	0.6	191.0565, 173.0456, 163.0402	5-*p*-Coumaroylquinic acid	Medium
13	4.99	C_45_H_36_O_18_	863.1865	863.1829	4.2	573.1047, 451.1063, 411.0740, 289.0710	Cinnamtannin B1	Low
14	5.13	C_30_H_24_O_12_	575.1215	575.1195	3.5	449.0891, 423.0745, 289.0695, 125.0229	Procyanidin A1	Medium
15	5.26	C_8_H_8_O_4_	167.0351	167.0350	0.6	152.0102, 109.0254, 108.0215	3,4-Dihydroxy-benzoic acid methyl ester	High
16	5.47	C_21_H_38_O_8_	417.2489	417.2494	−1.2	255.1964, 237.1824, 179.0568, 161.0454	Pumilaside A	Medium
17	5.66	C_20_H_30_O_10_	429.1767	429.1766	0.2	325.1152, 265.0941, 205.0724, 163.0611	2-Phenylethyl D-rutinoside	Medium
18	5.71	C_27_H_30_O_16_	609.1487	609.1461	4.3	301.0353, 300.0284, 271.0247, 255.0297, 178.9970, 151.0015	Rutin	High
19	6.17	C_30_H_24_O_12_	575.1215	575.1195	3.5	449.0891, 423.0745, 289.0695, 125.0229	Procyanidin A2	Medium
20	6.70	C_30_H_24_O_12_	575.1215	575.1195	3.5	449.0891, 423.0745, 289.0695, 125.0229	Procyanidin A5	Medium
21	7.24	C_27_H_32_O_14_	579.1752	579.1719	5.7	271.0605, 151.0022, 119.0521	Naringin	High
22	7.25	C_28_H_32_O_16_	623.1657	623.1618	6.3	315.0506, 300.0287	Narcissin	Medium
23	7.60	C_33_H_42_O_18_	725.2339	725.2298	5.7	271.0621, 151.0033	Naringenin 7-O-(2,6-di-O-*α*-L-rhamnopyranosyl)-*β*-D-glucopyranoside	Medium
24	8.27	C_9_H_10_O_4_	181.0507	181.0506	0.5	153.0187, 152.0091, 109.0296, 108.0222	Ethyl 3,4-dihydroxybenzoate	High
25	8.70	C_19_H_32_O_10_	419.1930	419.1923	1.7	239.1294, 195.1386, 161.0433	Youngiajaponicoside C	Medium
26	9.00	C_19_H_34_O_9_	405.2129	405.2130	−0.2	225.1493, 179.0563, 161.0458	Kiwiionoside	Medium

## Data Availability

The datasets generated and analyzed during the current study are available from the corresponding author upon reasonable request.

## Supplementary Materials

The following supporting information can be downloaded at https://www.mdpi.com/article/10.3390/foods15193524/s1. The original uncropped Western blot images for representative manuscript figures and the full UPLC-Q-TOF-MS/MS spectra together with fragment assignment data of LCS2 are publicly available at Zenodo: https://zenodo.org/records/22142962, accessed on 28 August 2026.

## Abbreviations

The following abbreviations are used in this manuscript:

BCA	Bicinchoninic Acid
COX-2	Cyclooxygenase-2
DCFH_2_-DA	2′,7′-Dichlorodihydrofluorescein Diacetate
DMEM	Dulbecco’s Modified Eagle Medium
DMSO	Dimethyl Sulfoxide
ECL	Enhanced Chemiluminescence
ELISA	Enzyme-Linked Immunosorbent Assay
FBS	Fetal Bovine Serum
GAPDH	Glyceraldehyde-3-Phosphate Dehydrogenase
H&E	Hematoxylin and Eosin
IHC	Immunohistochemistry
IKKα/β	Inhibitor of Nuclear Factor κB Kinase α/β
IκBα	Inhibitor of Nuclear Factor κB α
IL-1β	Interleukin-1β
IL-6	Interleukin-6
iNOS	Inducible Nitric Oxide Synthase
LCS2	Litchi Seed Bioactive Fraction 2
LPS	Lipopolysaccharide
MAPK	Mitogen-Activated Protein Kinase
MTT	3-(4,5-Dimethylthiazol-2-yl)-2,5-diphenyltetrazolium Bromide
NF-κB	Nuclear Factor κ-Light-Chain-Enhancer of Activated B Cells
NO	Nitric Oxide
PBS	Phosphate-Buffered Saline
PGE_2_	Prostaglandin E_2_
PVDF	Polyvinylidene Fluoride
ROS	Reactive Oxygen Species
RT-qPCR	Reverse Transcription-Quantitative Polymerase Chain Reaction
SDS-PAGE	Sodium Dodecyl Sulfate-Polyacrylamide Gel Electrophoresis
TH	Tamsulosin Hydrochloride
TNF-α	Tumor Necrosis Factor-α
UPLC-Q-TOF-MS/MS	Ultra-Performance Liquid Chromatography Coupled with Quadrupole Time-of-Flight Tandem Mass Spectrometry
