# Supplementary material for: Bioactive Fraction LCS2 from Litchi (Litchi chinensis Sonn.) Seed By-Product Attenuates LPS-Induced Acute Prostatic Inflammatory Injury via Modulating NF-κB and MAPK Signaling Pathways
Source: Foods. 2026 Oct 2;15(19):3524. doi: 10.3390/foods15193524 (PMC13665308; doi:10.3390/foods15193524)
Supplement: Supplementary file 1 [file foods-15-03524-s001.zip › foods-4561395-supplementary.pdf]

UPLC-Q-TOF-MS/MS Spectra and Fragment Assignment of LCS2

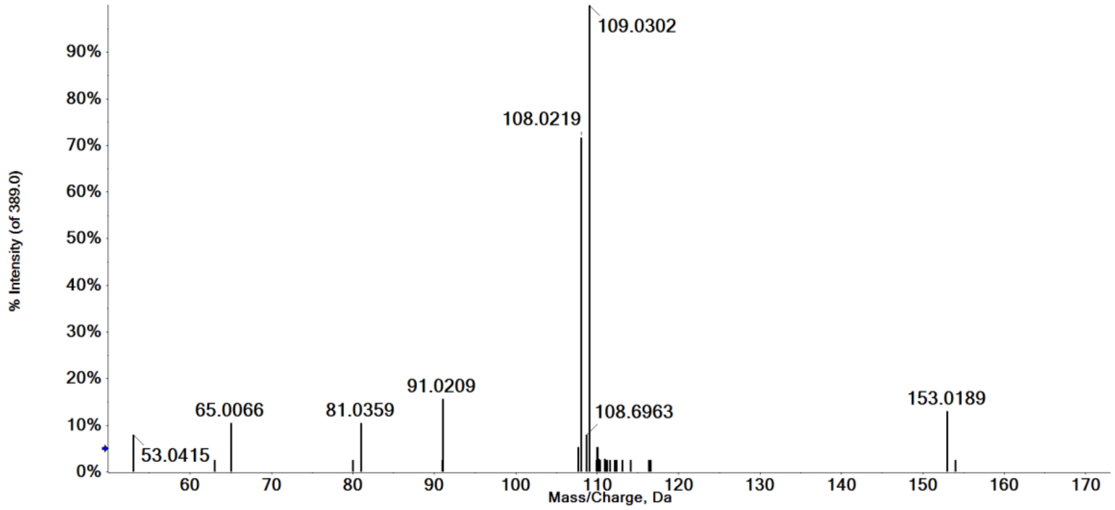

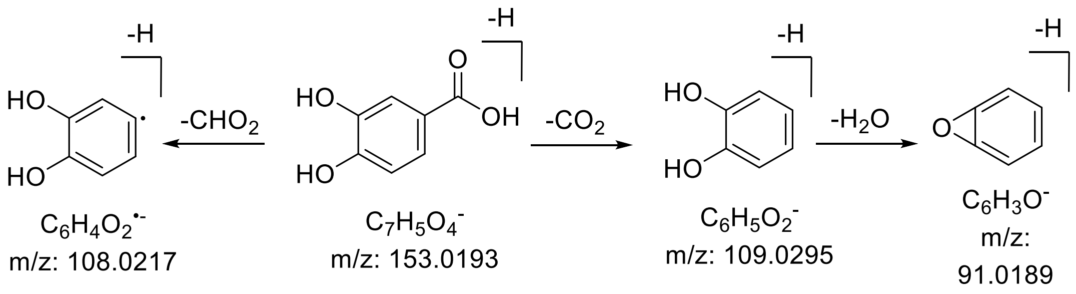

Figure S1. MS/MS spectrum of Protocatechuic acid (peak 1, tR = 2.14 min) in ESI negative ion mode.

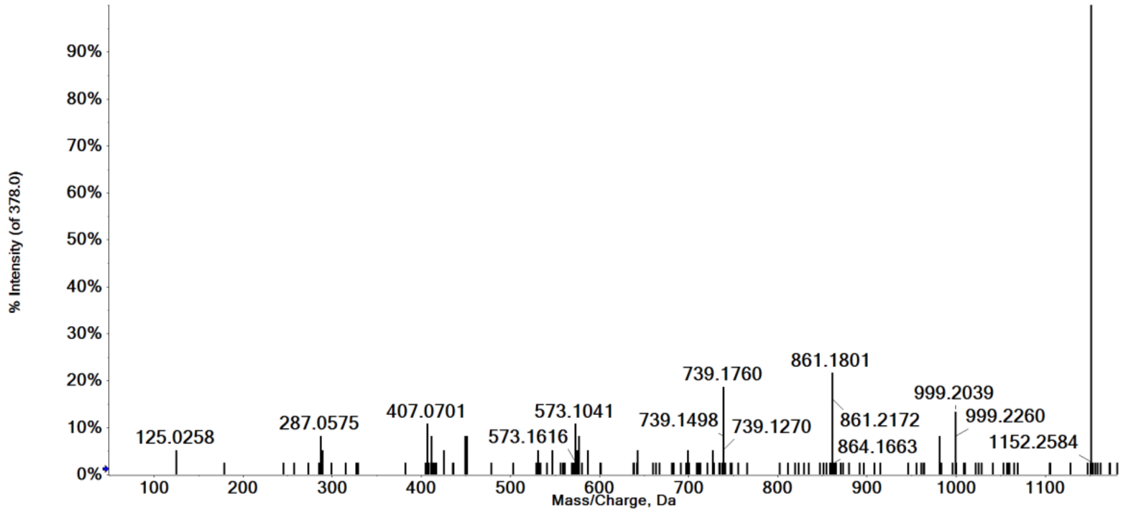

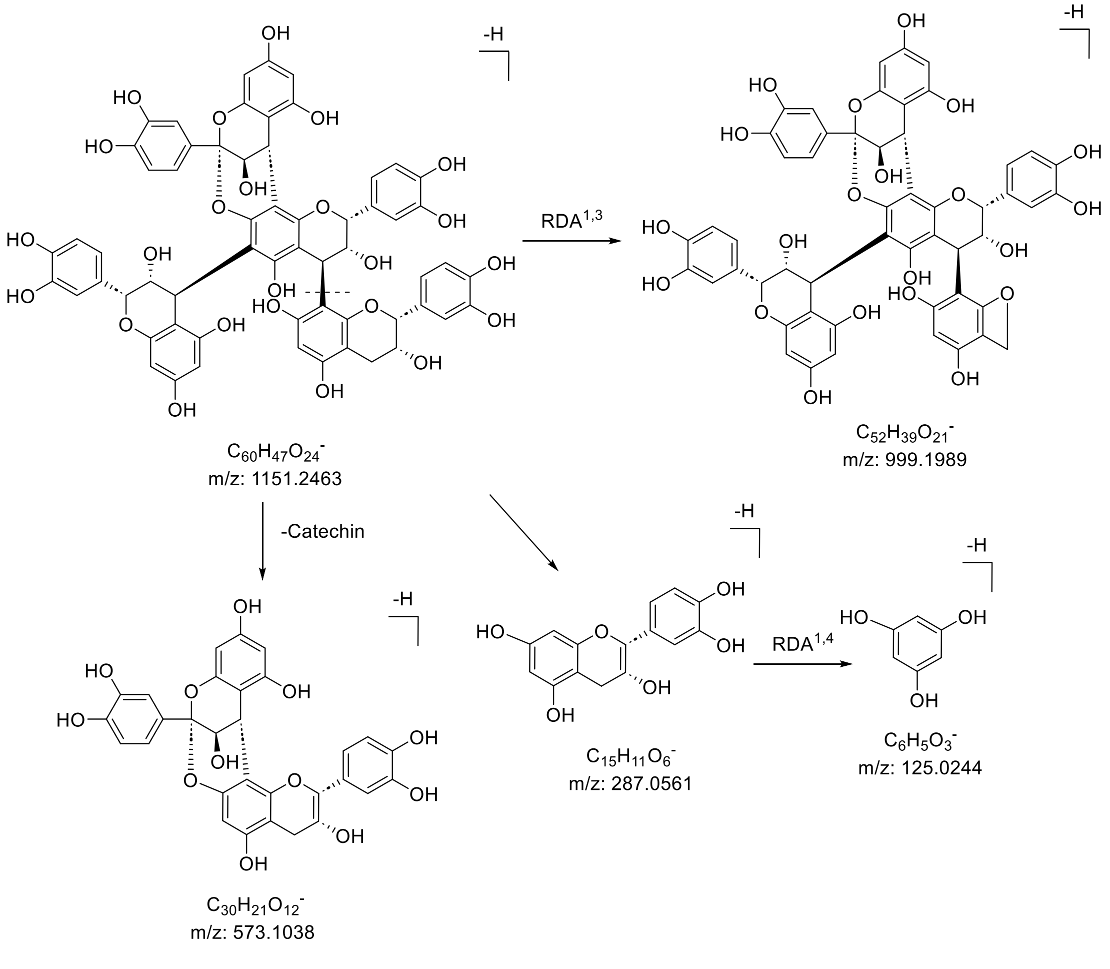

Figure S2. MS/MS spectrum of Parameritannin A1 (peak 2, tR = 2.15 min) in ESI negative ion mode.

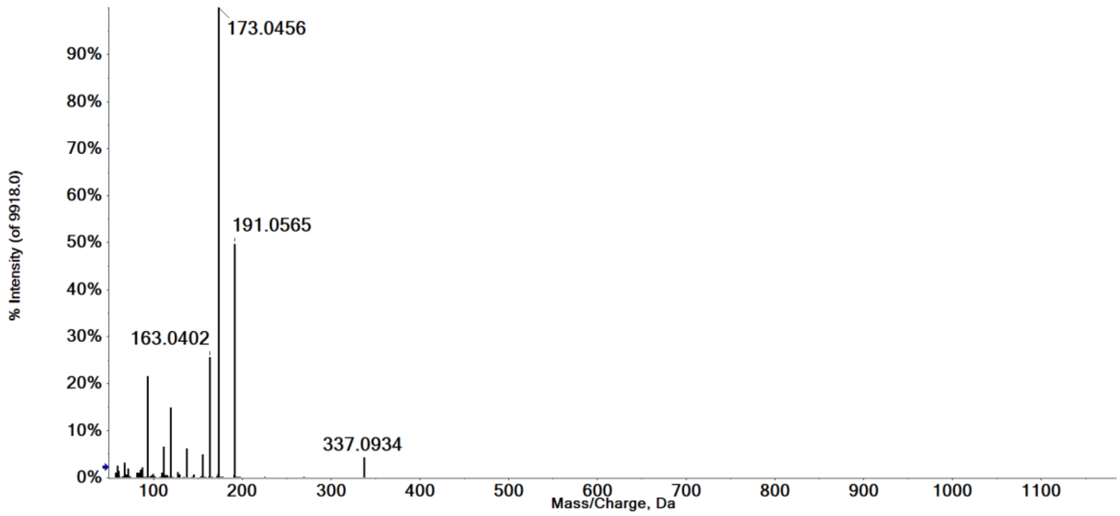

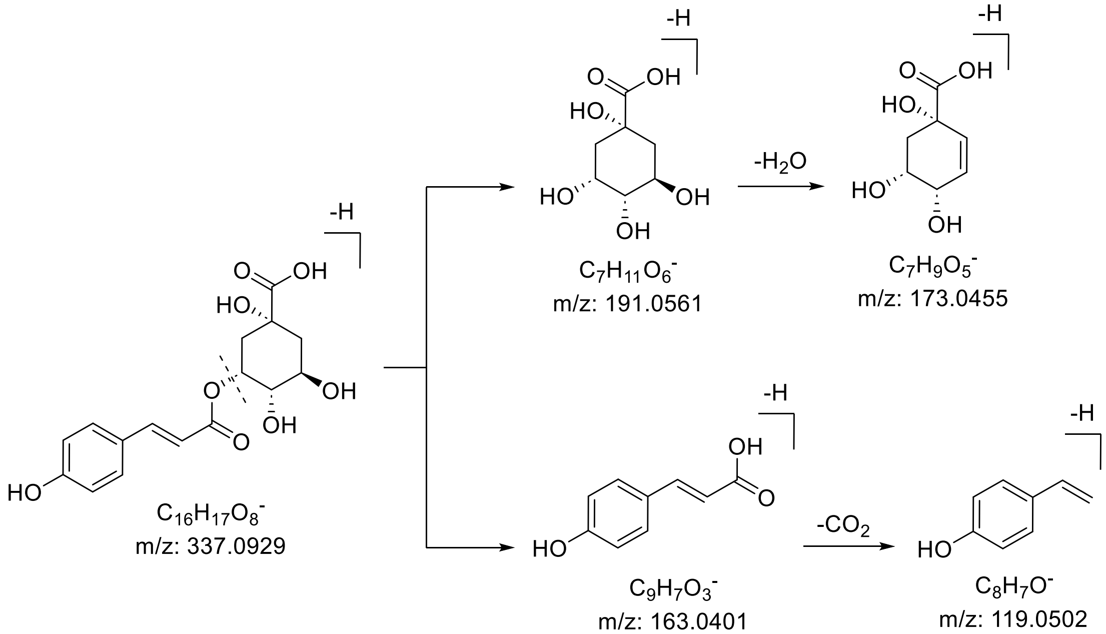

Figure S3. MS/MS spectrum of 3-*p*-Coumaroylquinic acid (peak 3, tR = 2.66 min) in ESI negative ion mode.

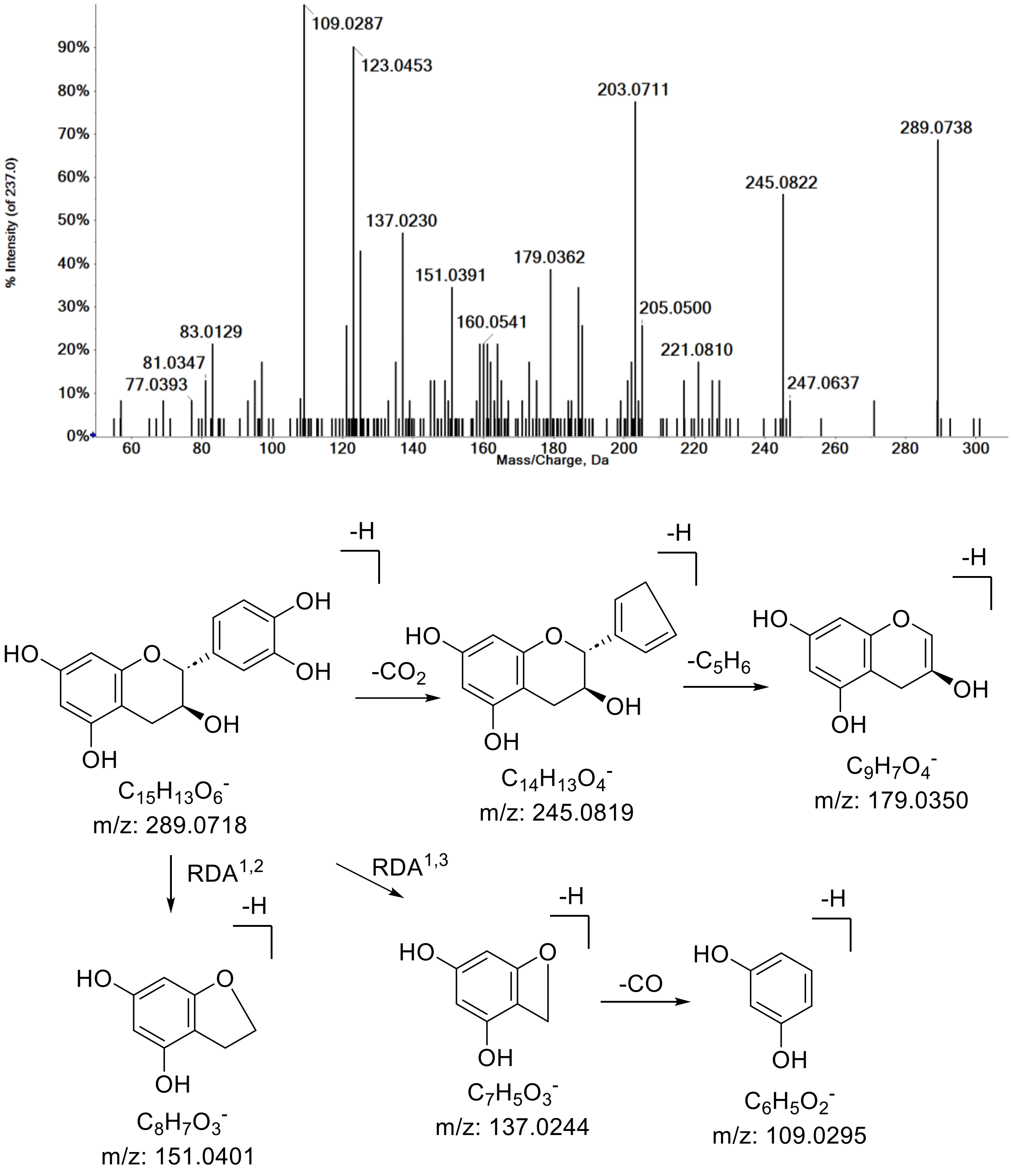

Figure S4. MS/MS spectrum of Catechin (peak 4, tR = 2.75 min) in ESI negative ion mode.

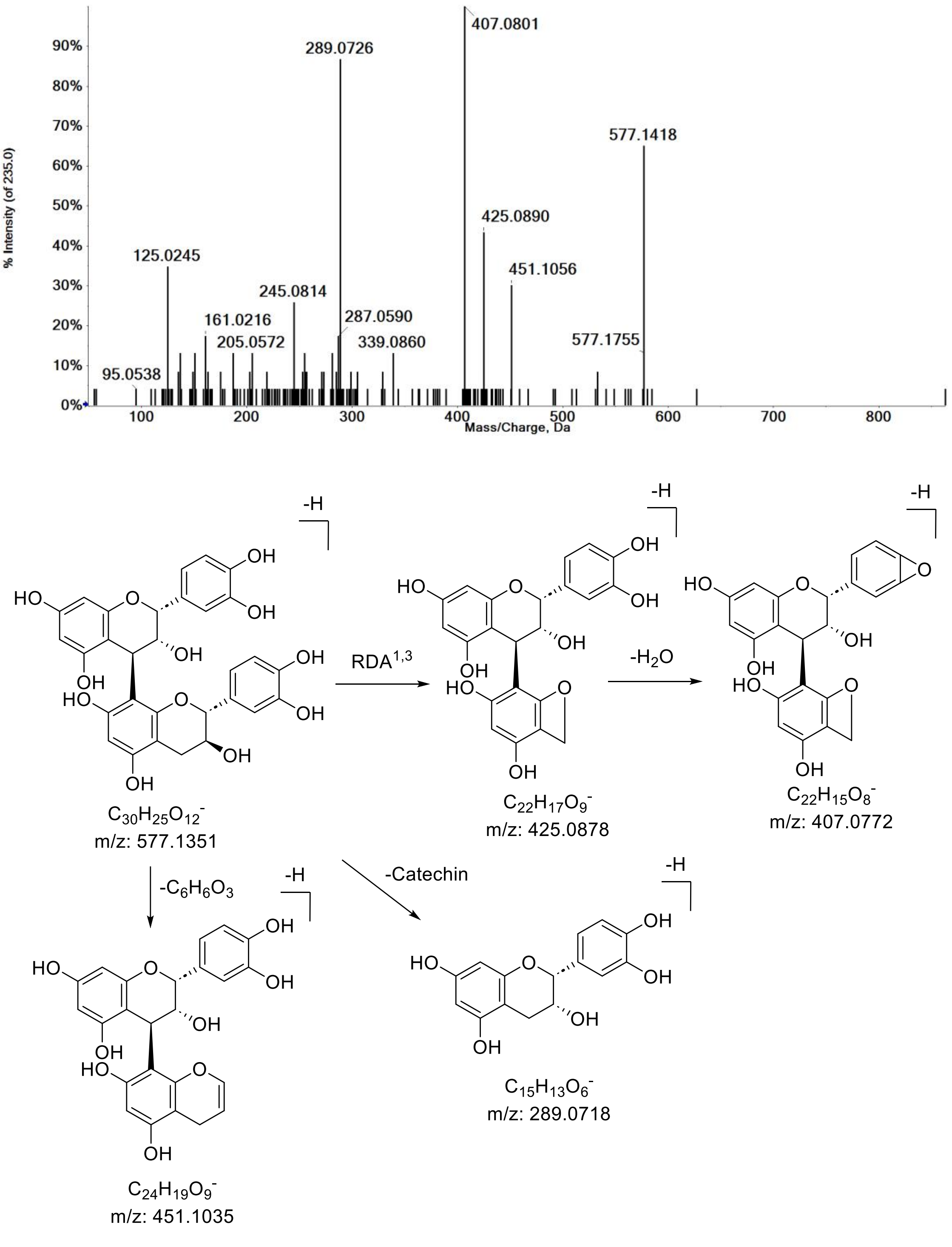

Figure S5. MS/MS spectrum of Procyanidin B (peak 5, tR = 3.11 min) in ESI negative ion mode.

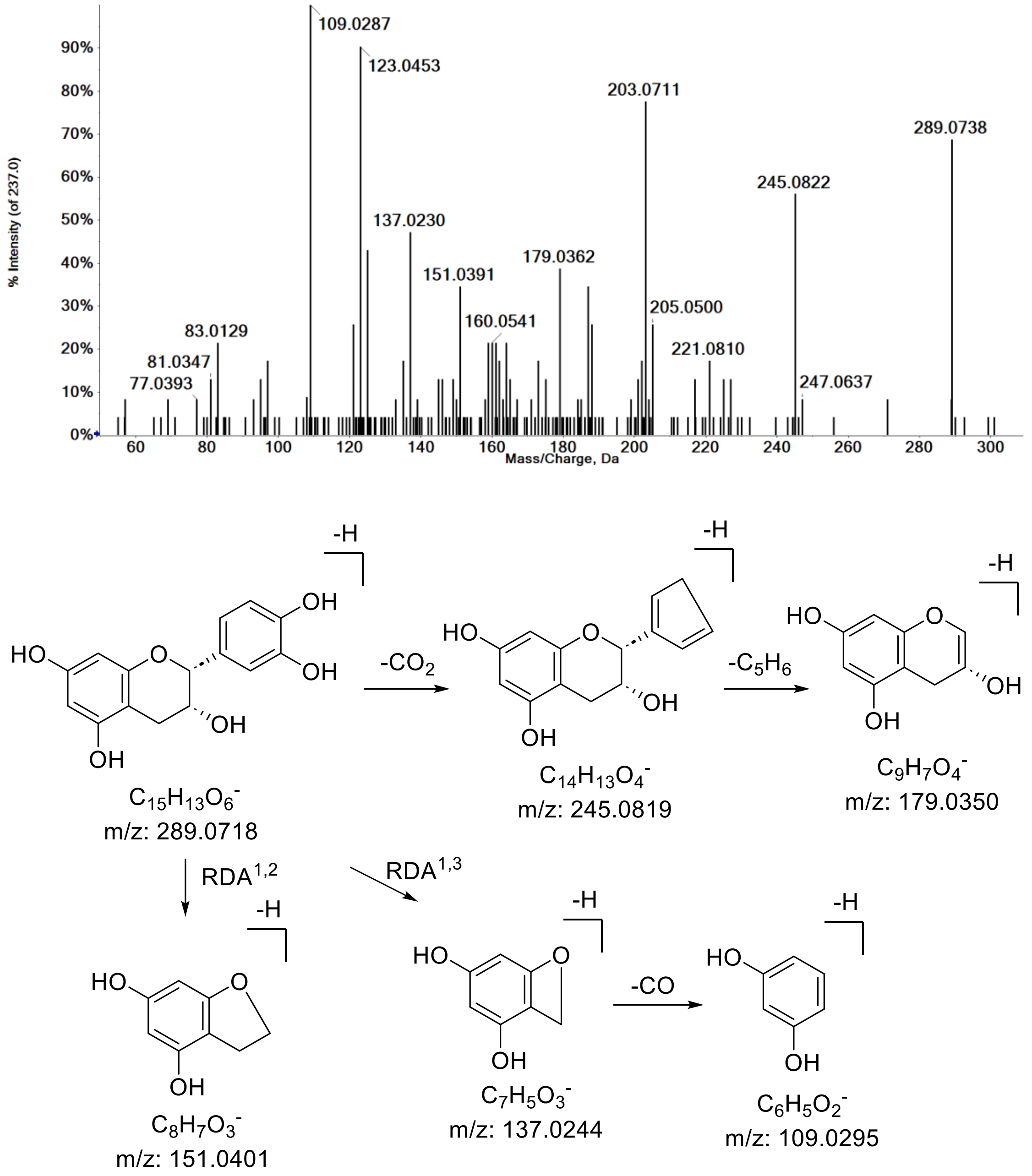

Figure S6. MS/MS spectrum of Epicatechin (peak 6, tR = 3.65 min) in ESI negative ion mode.

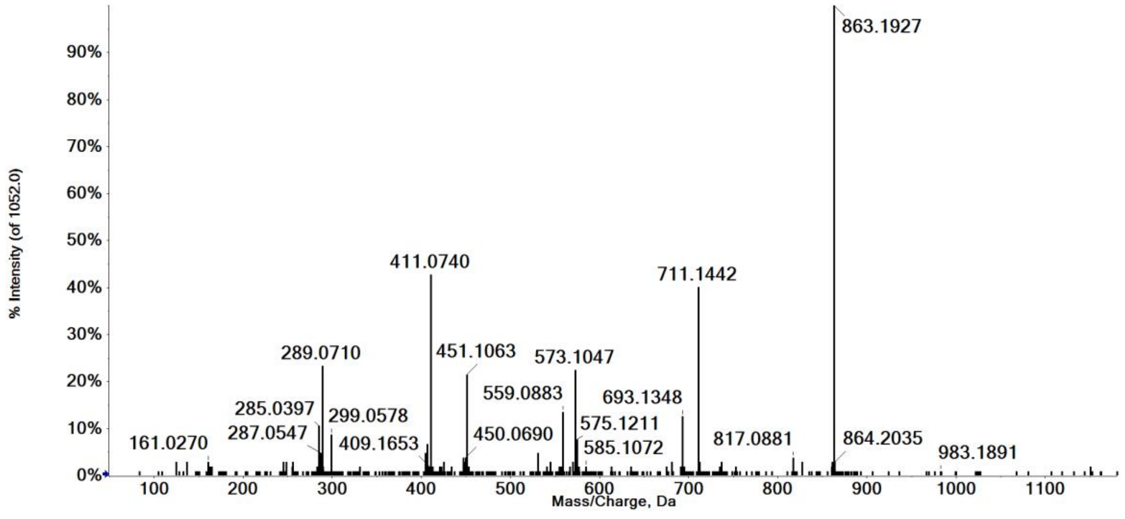

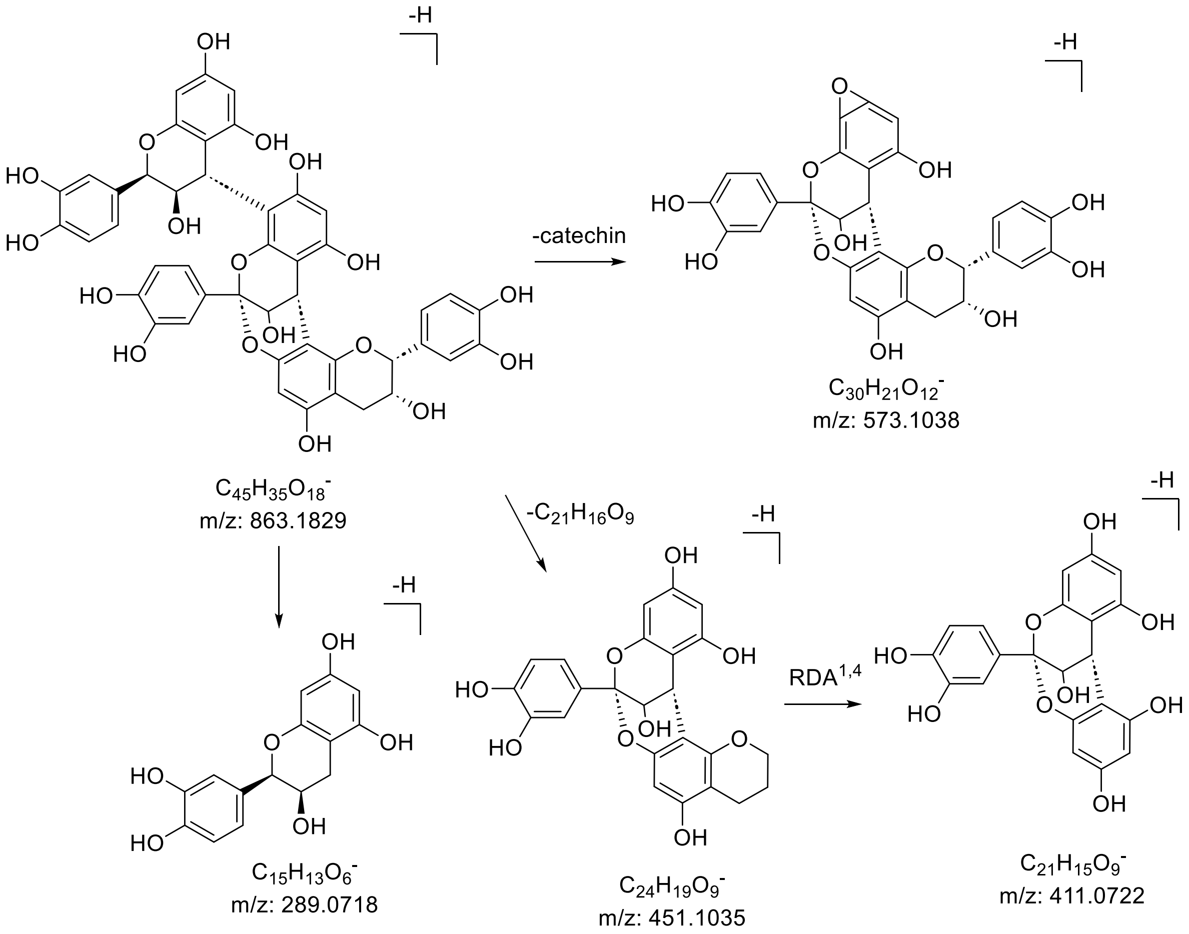

Figure S7. MS/MS spectrum of Aesculitannin A (peak 7, tR = 3.80 min) in ESI negative ion mode.

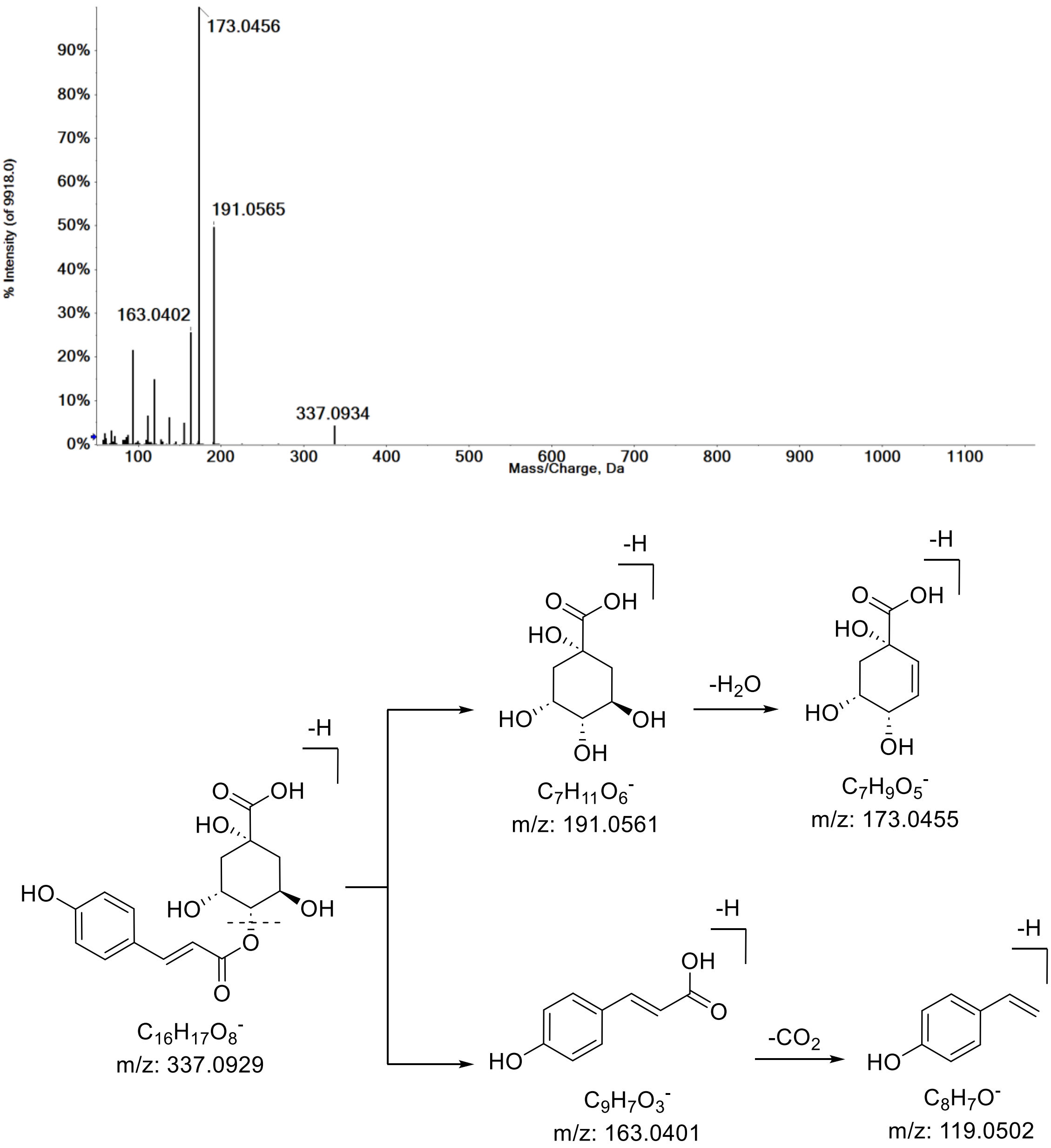

Figure S8. MS/MS spectrum of 4-*p*-Coumaroylquinic acid (peak 8, tR = 3.85 min) in ESI negative ion mode.

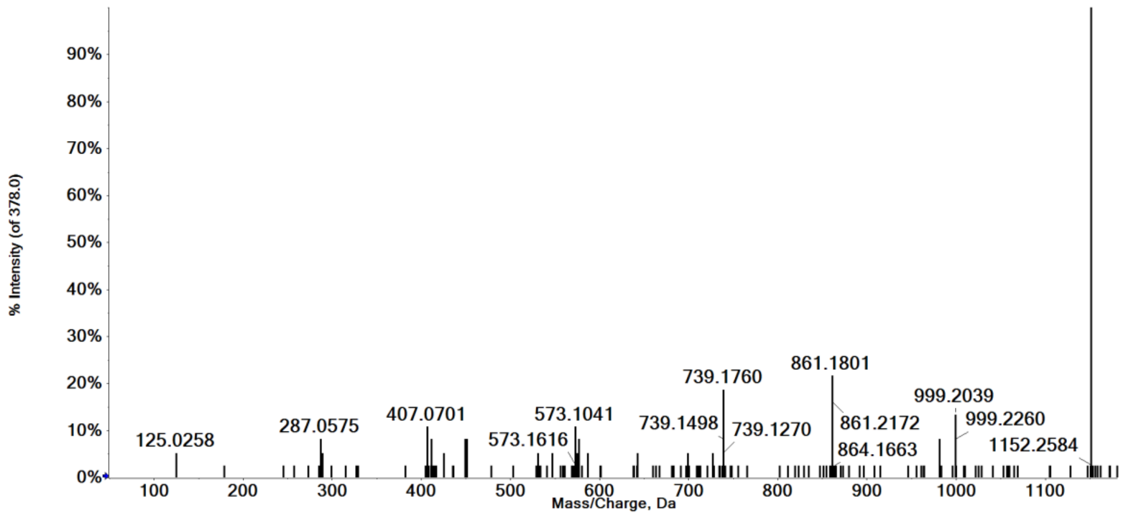

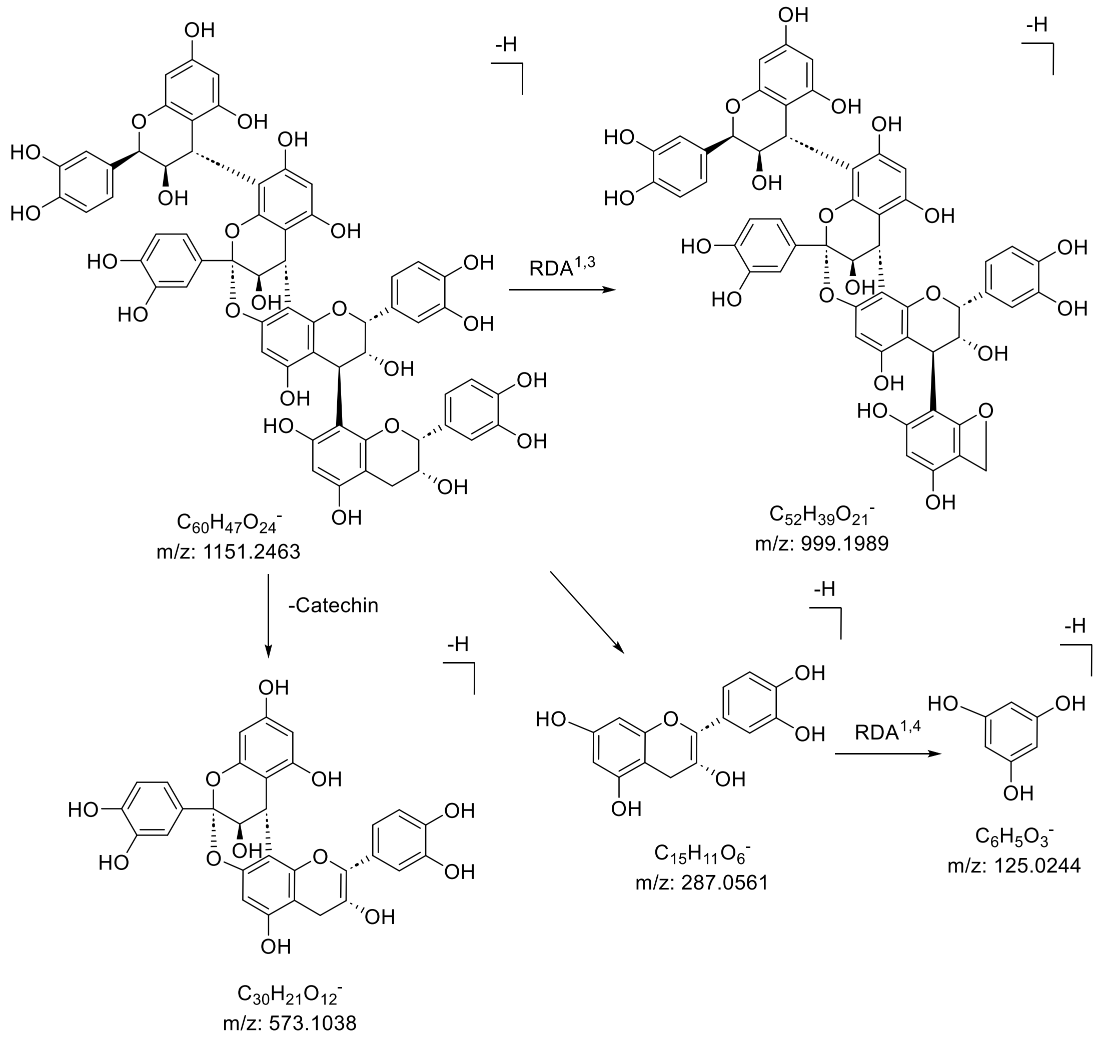

Figure S9. MS/MS spectrum of Cinnamtannin B2 (peak 9, tR = 4.09 min) in ESI negative ion mode.

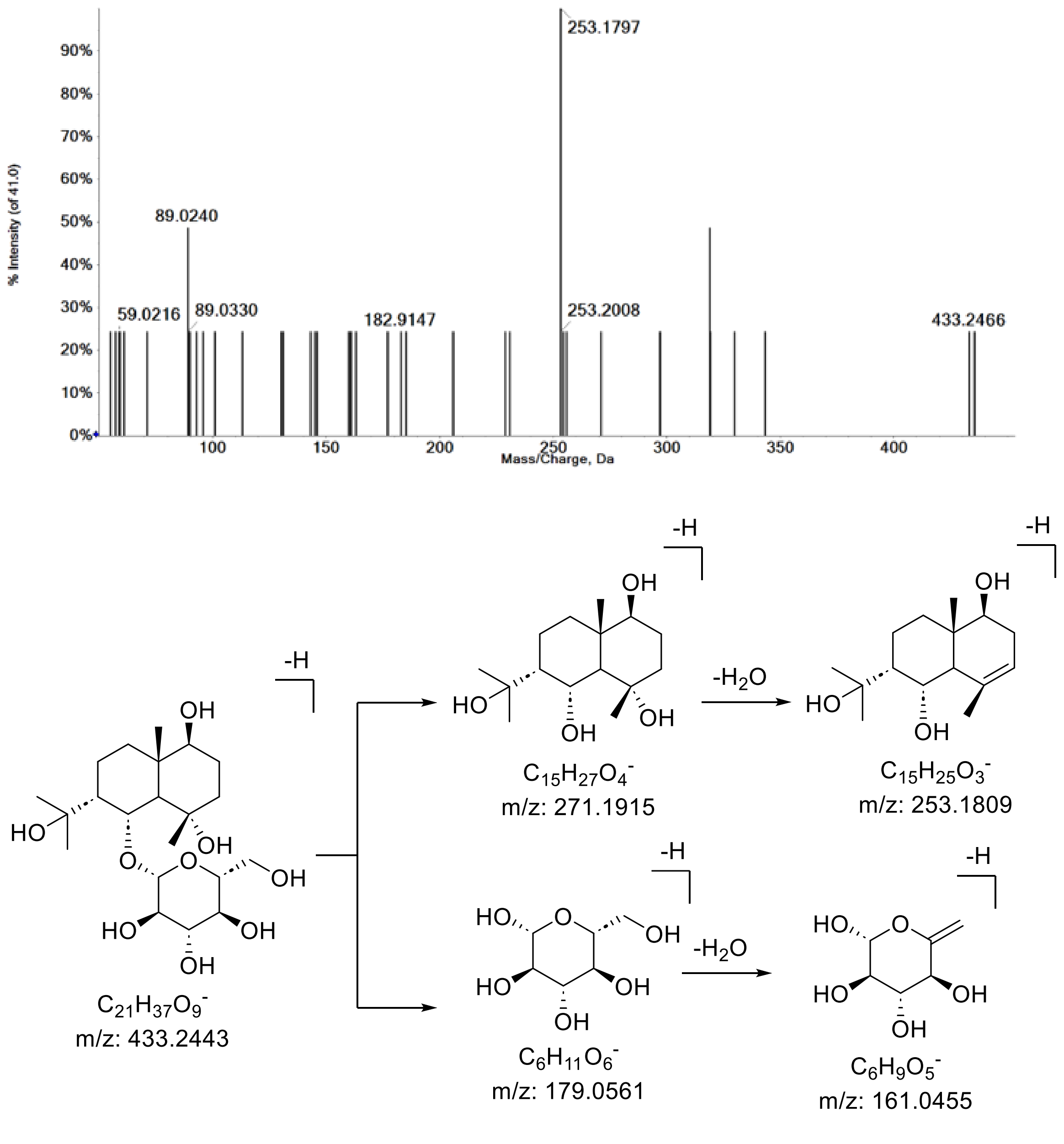

Figure S10. MS/MS spectrum of Dictamnoside J (peak 10, tR = 4.16 min) in ESI negative ion mode.

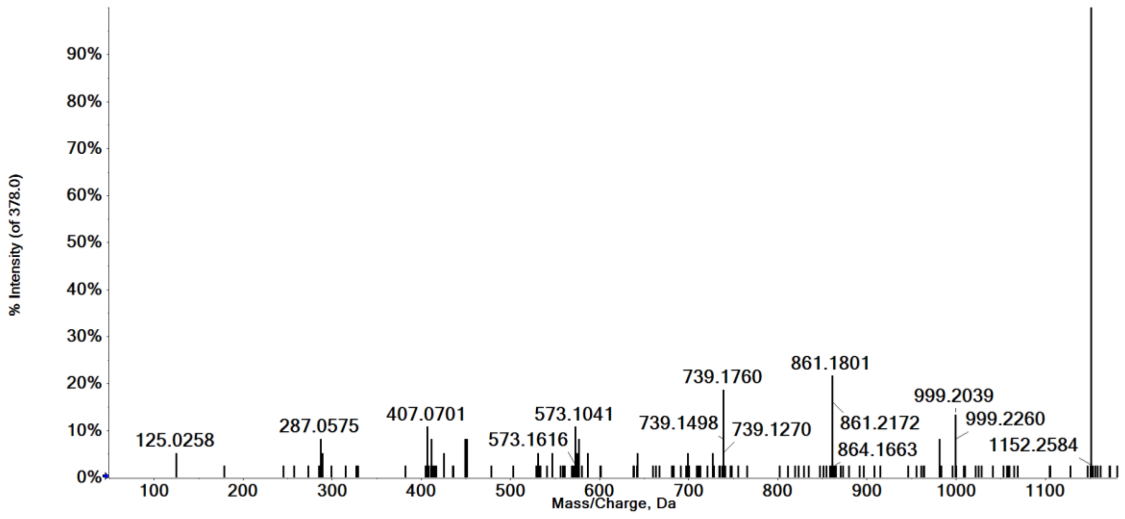

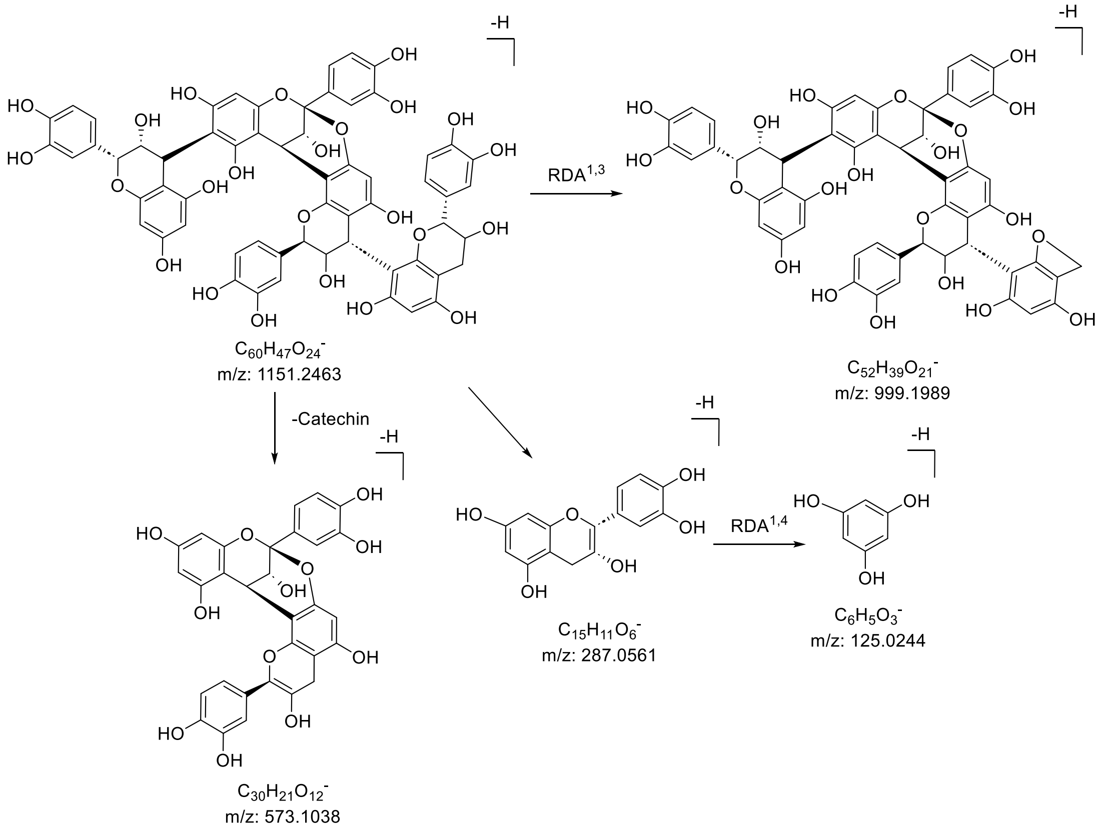

Figure S11. MS/MS spectrum of Pavetannin C1 (peak 11, tR = 4.75 min) in ESI negative ion mode.

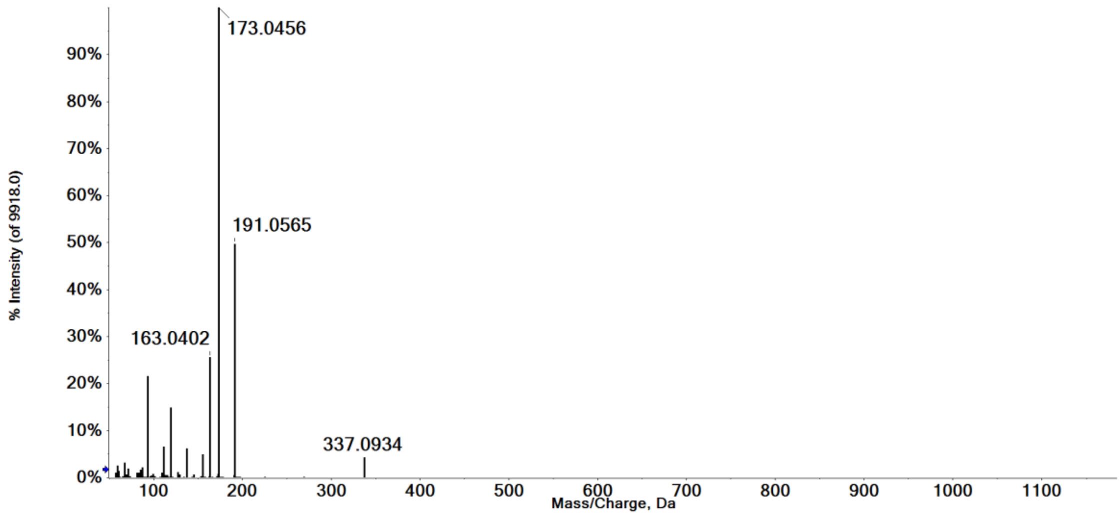

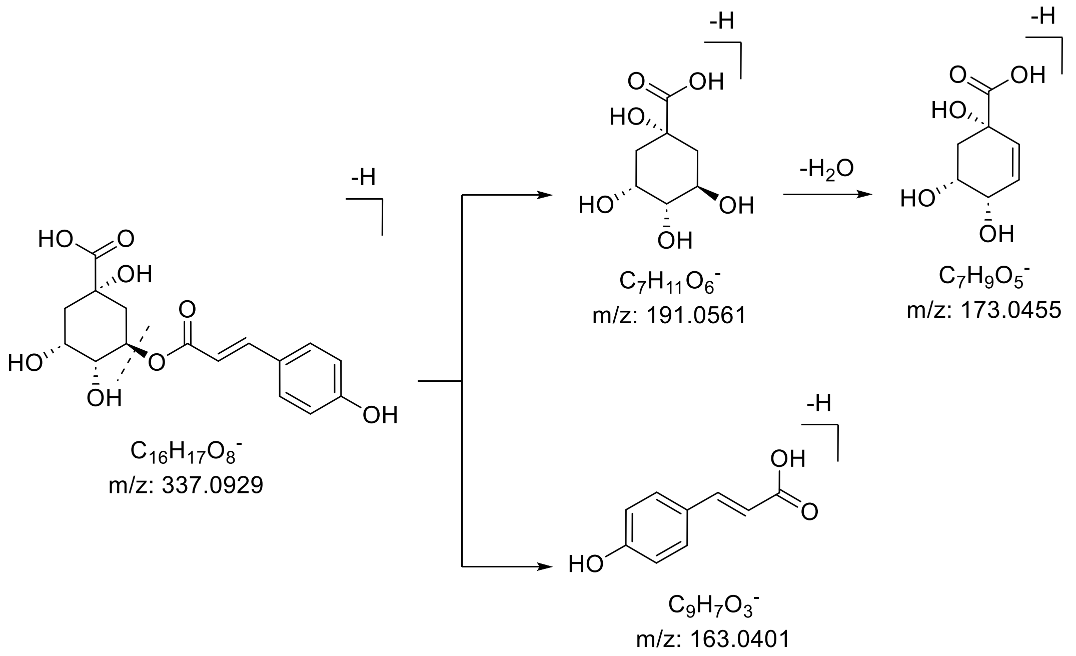

Figure S12. MS/MS spectrum of 5-*p*-Coumaroylquinic acid (peak 12, tR = 4.88 min) in ESI negative ion mode.

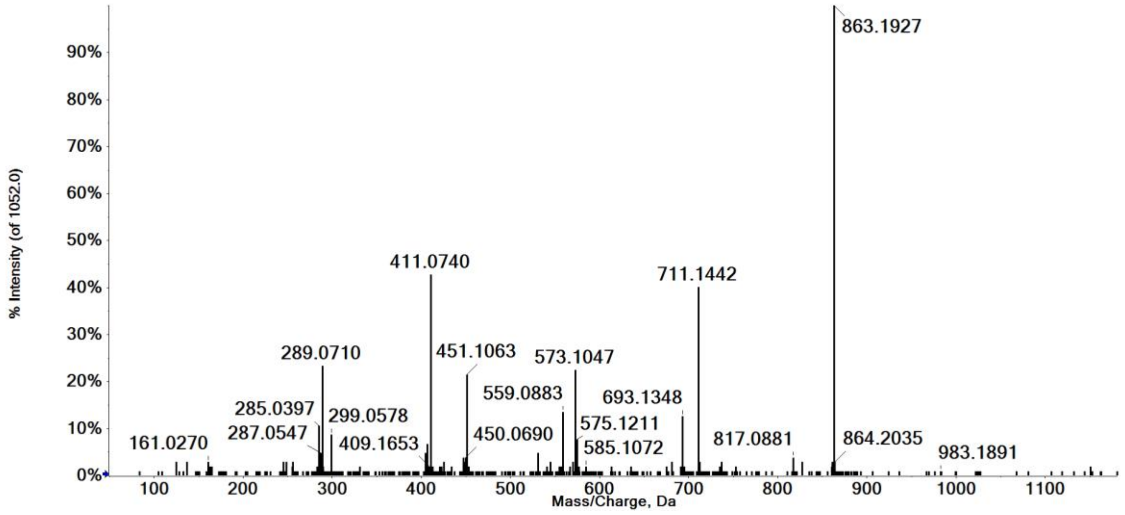

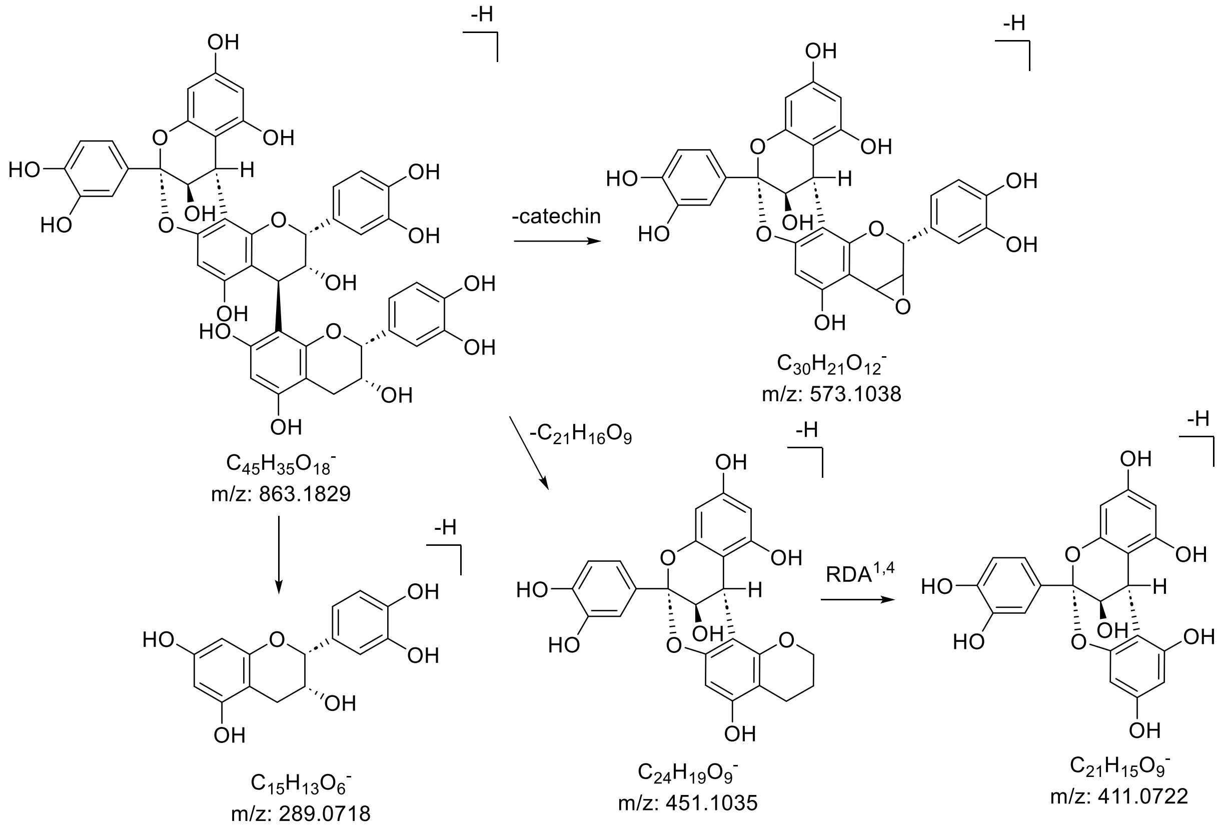

Figure S13. MS/MS spectrum of Cinnamtannin B1 (peak 13, tR = 4.99 min) in ESI negative ion mode.

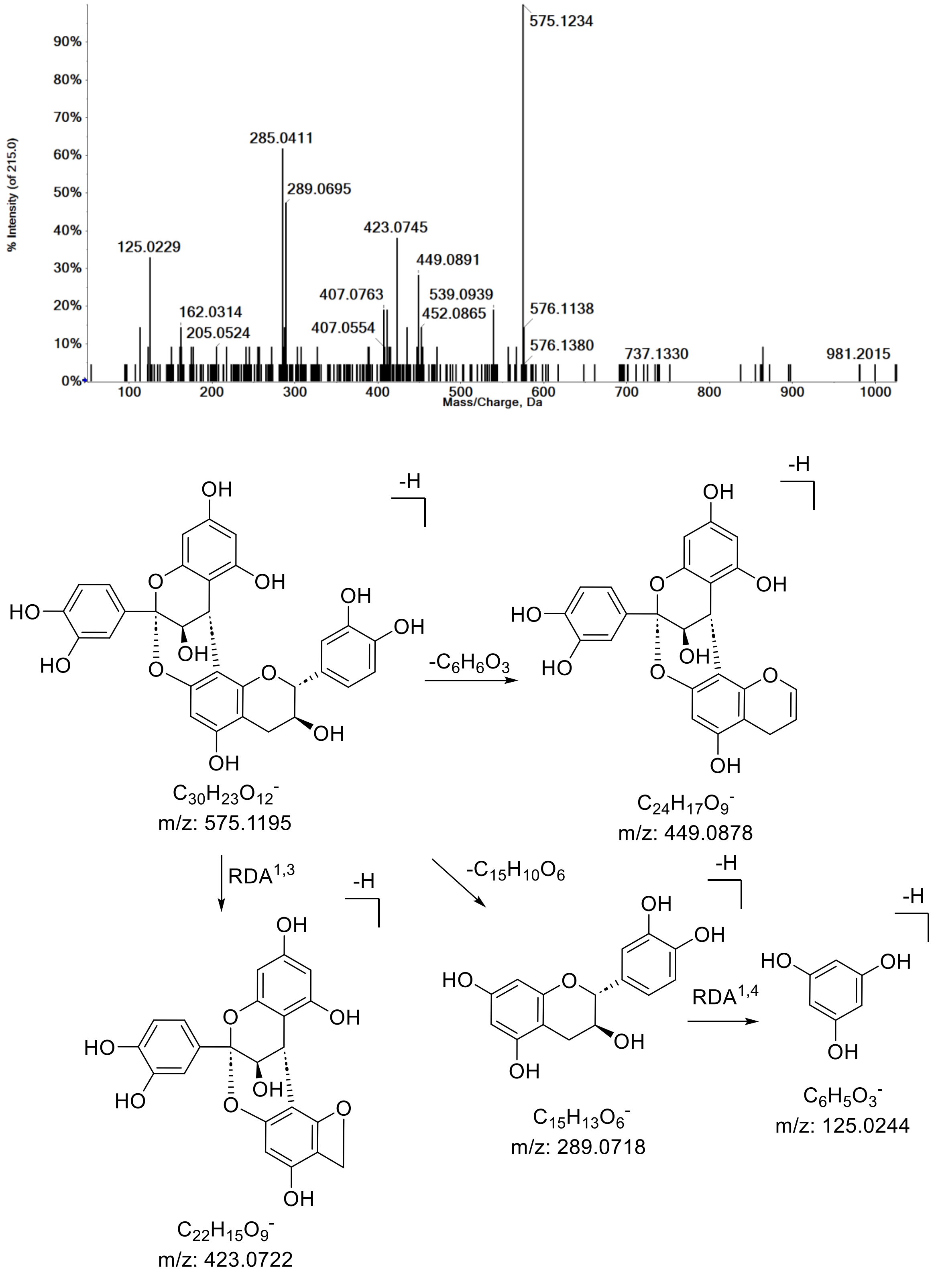

Figure S14. MS/MS spectrum of Procyanidin A1 (peak 14, tR = 5.13 min) in ESI negative ion mode.

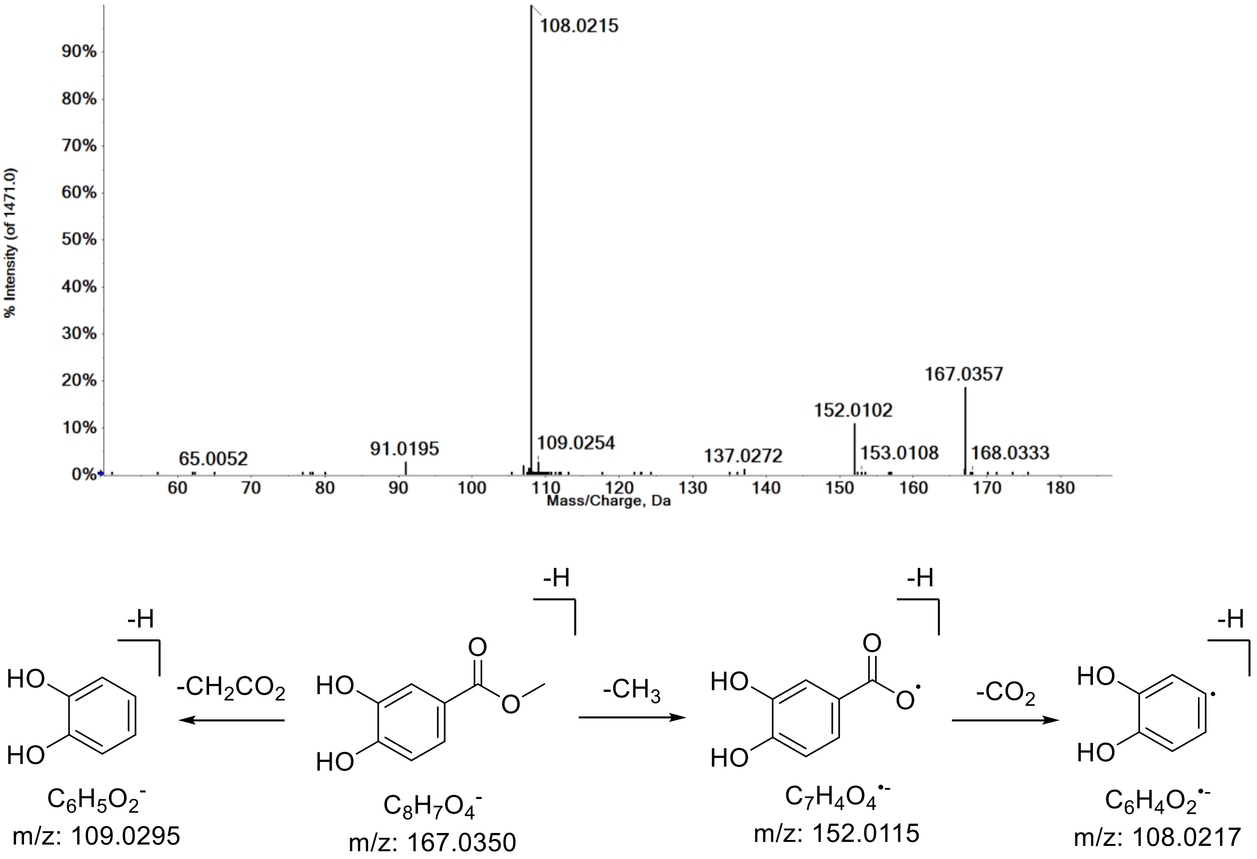

Figure S15. MS/MS spectrum of 3,4-Dihydroxy-benzoic acid methyl ester (peak 15, tR = 5.26 min) in ESI negative ion mode.

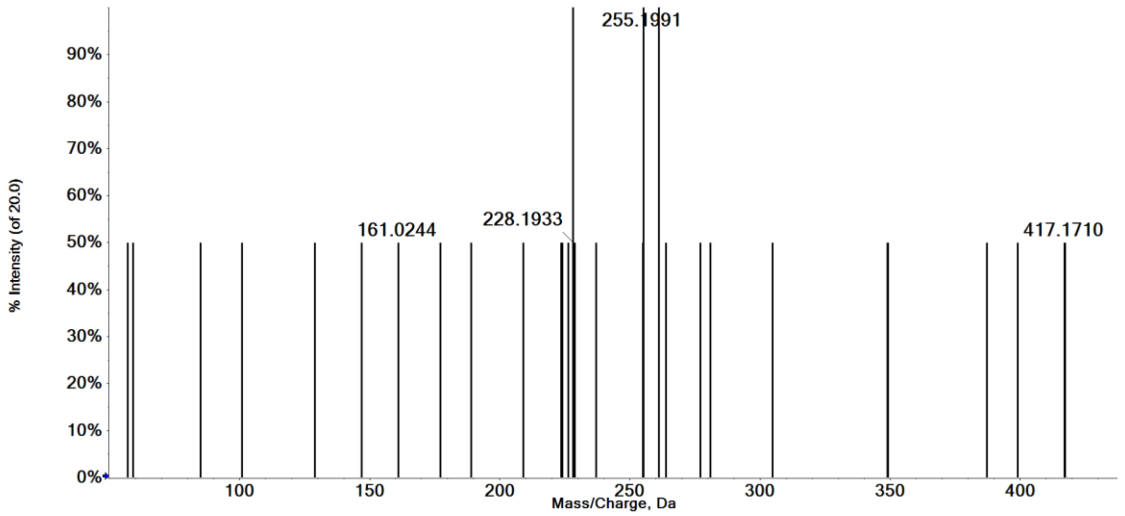

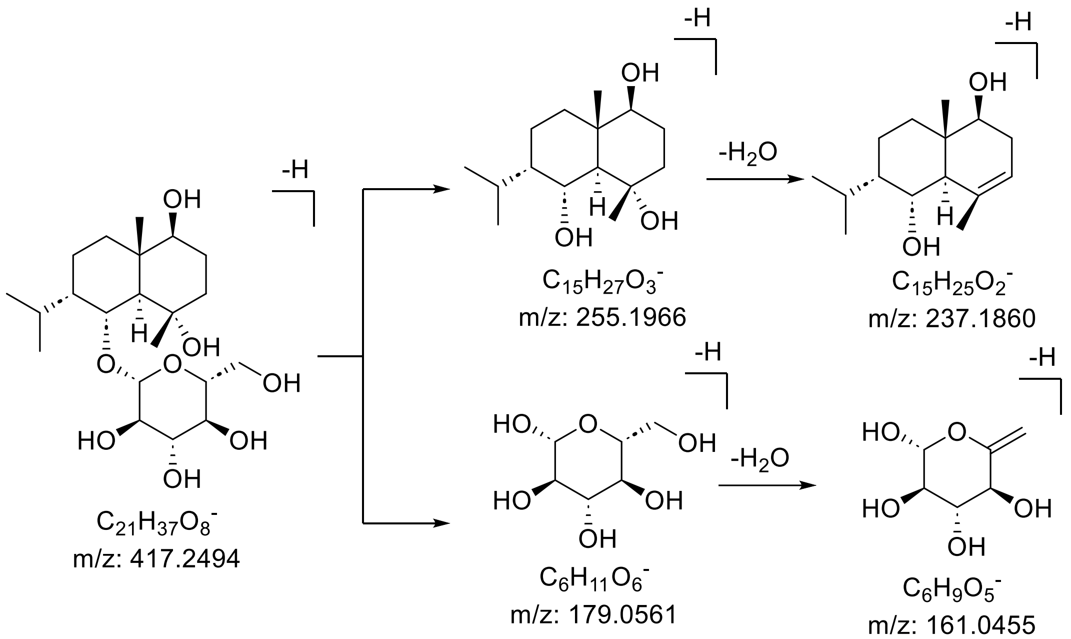

Figure S16. MS/MS spectrum of Pumilaside A (peak 16, tR = 5.47 min) in ESI negative ion mode.

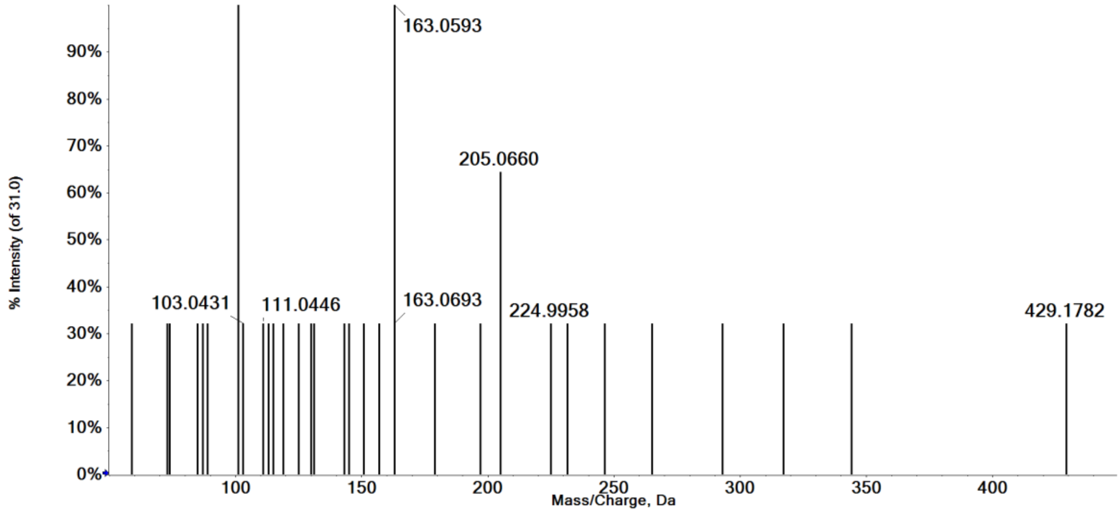

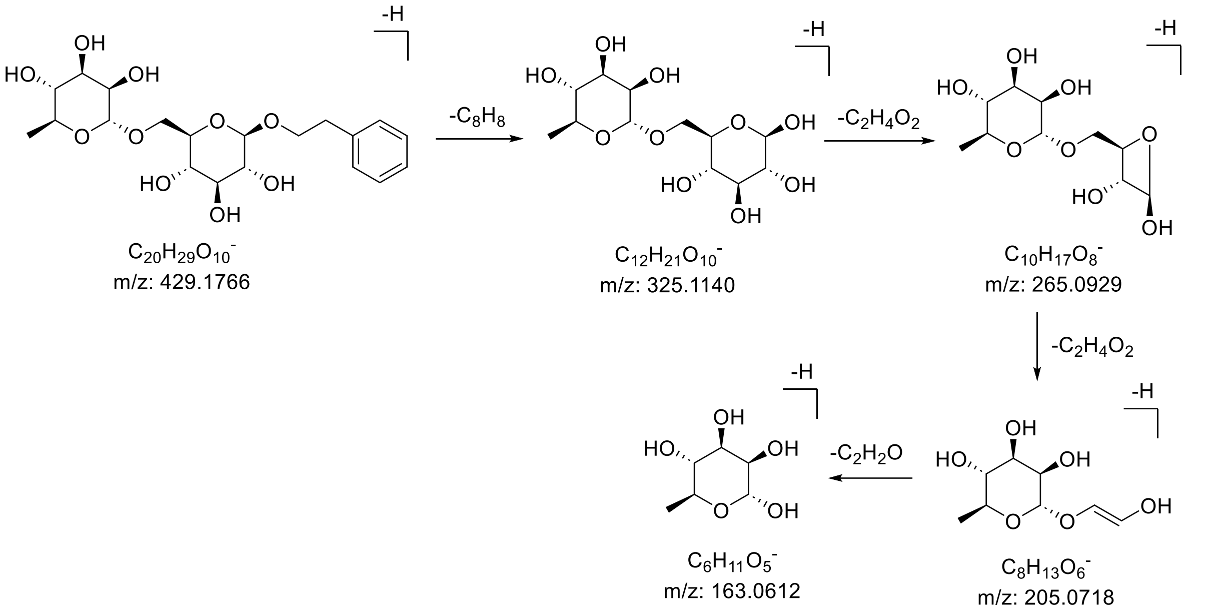

Figure S17. MS/MS spectrum of 2-Phenylethyl D-rutinoside (peak 17, tR = 5.66 min) in ESI negative ion mode.

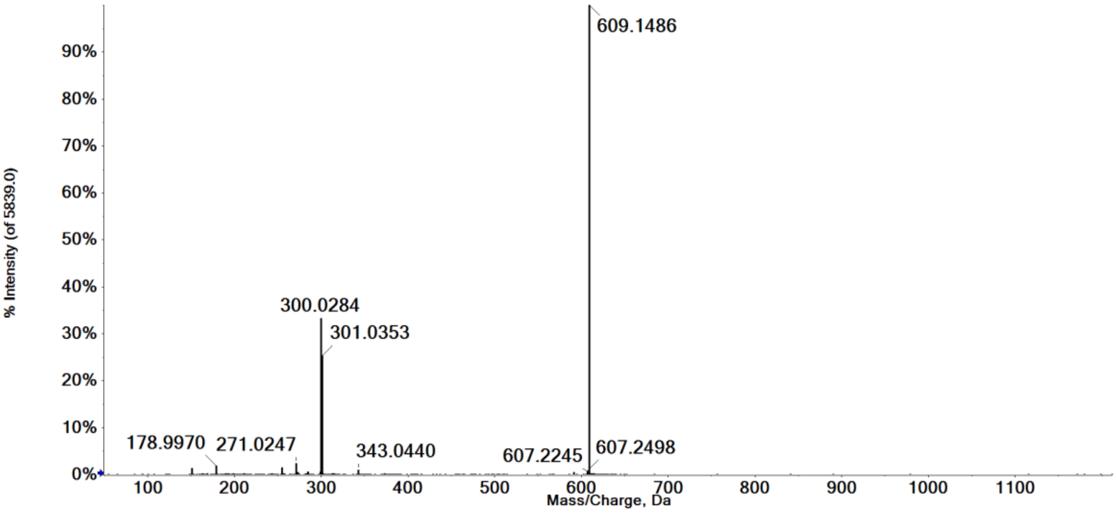

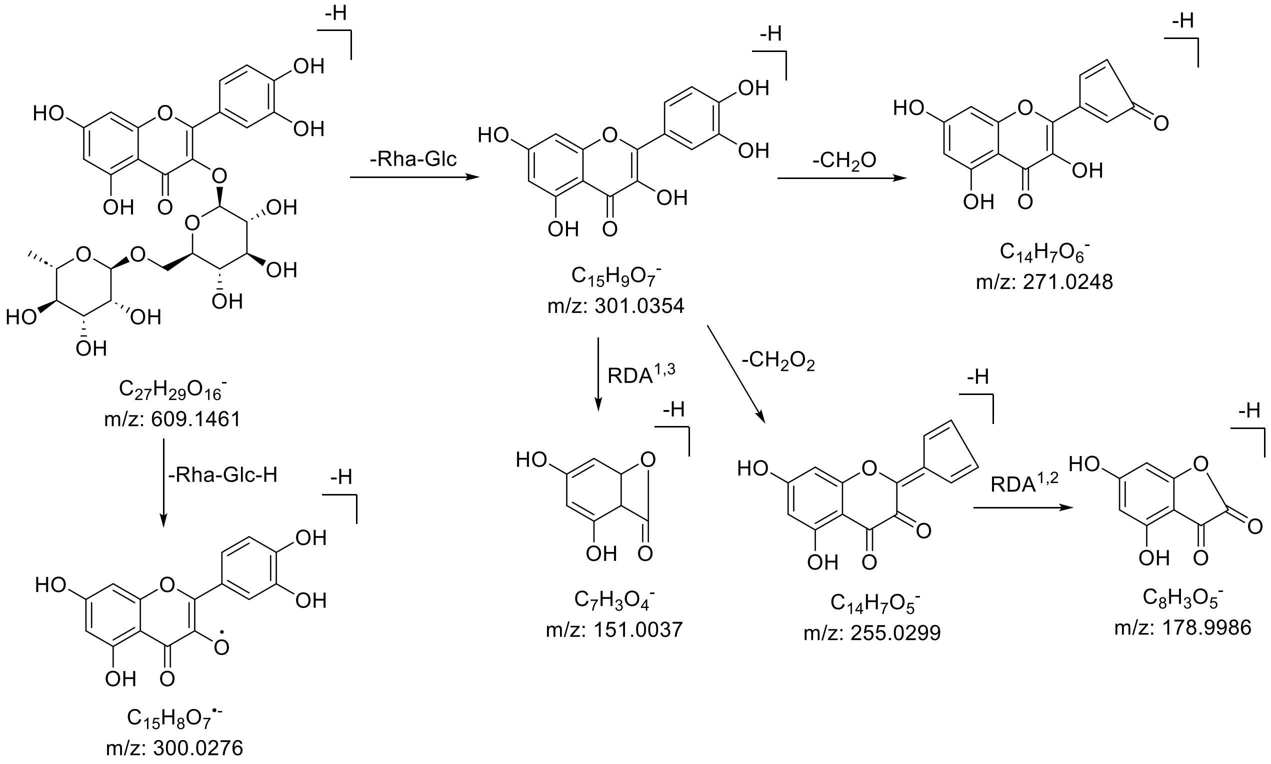

Figure S18. MS/MS spectrum of Rutin (peak 18, tR = 5.71 min) in ESI negative ion mode.

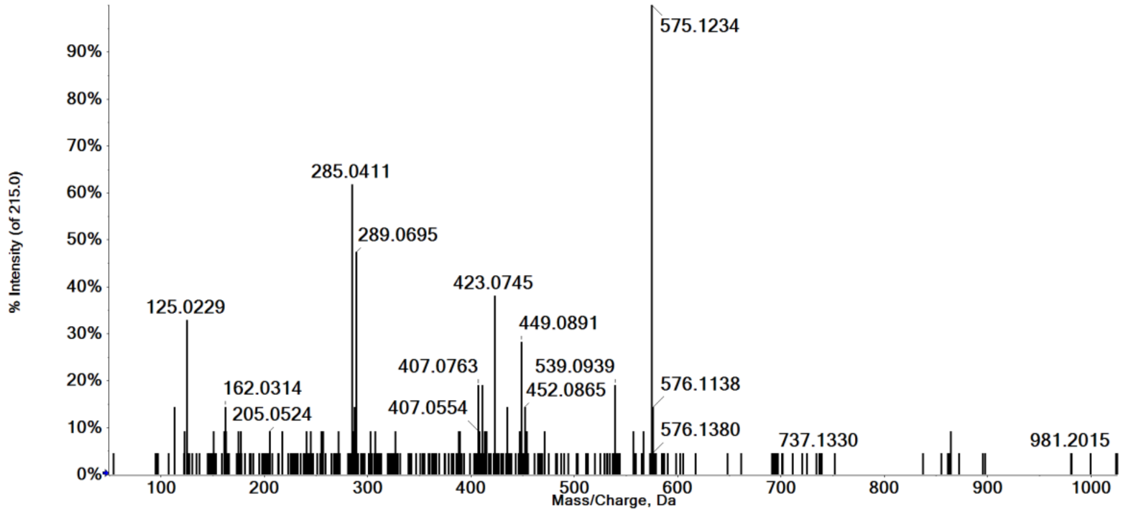

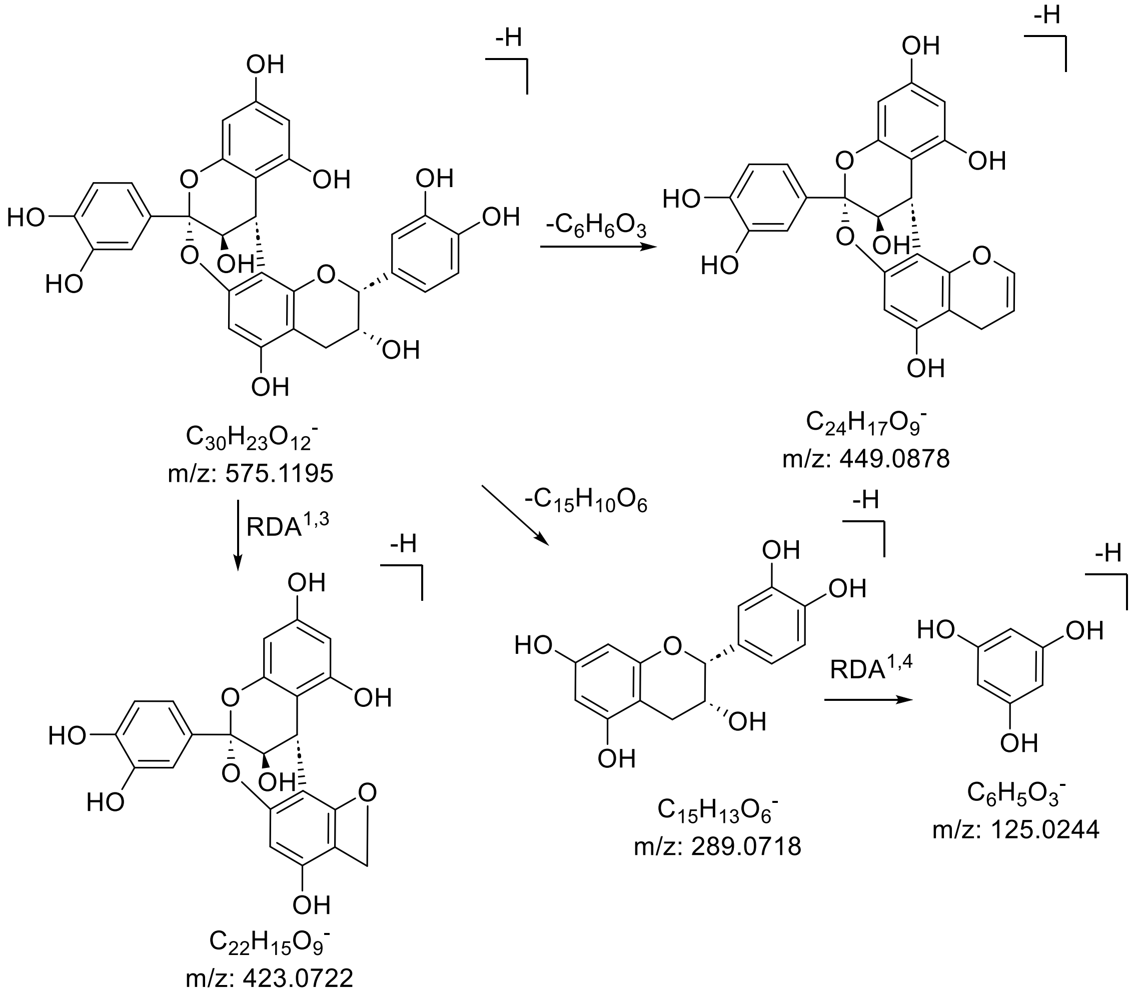

Figure S19. MS/MS spectrum of Procyanidin A2 (peak 19, tR = 6.17 min) in ESI negative ion mode.

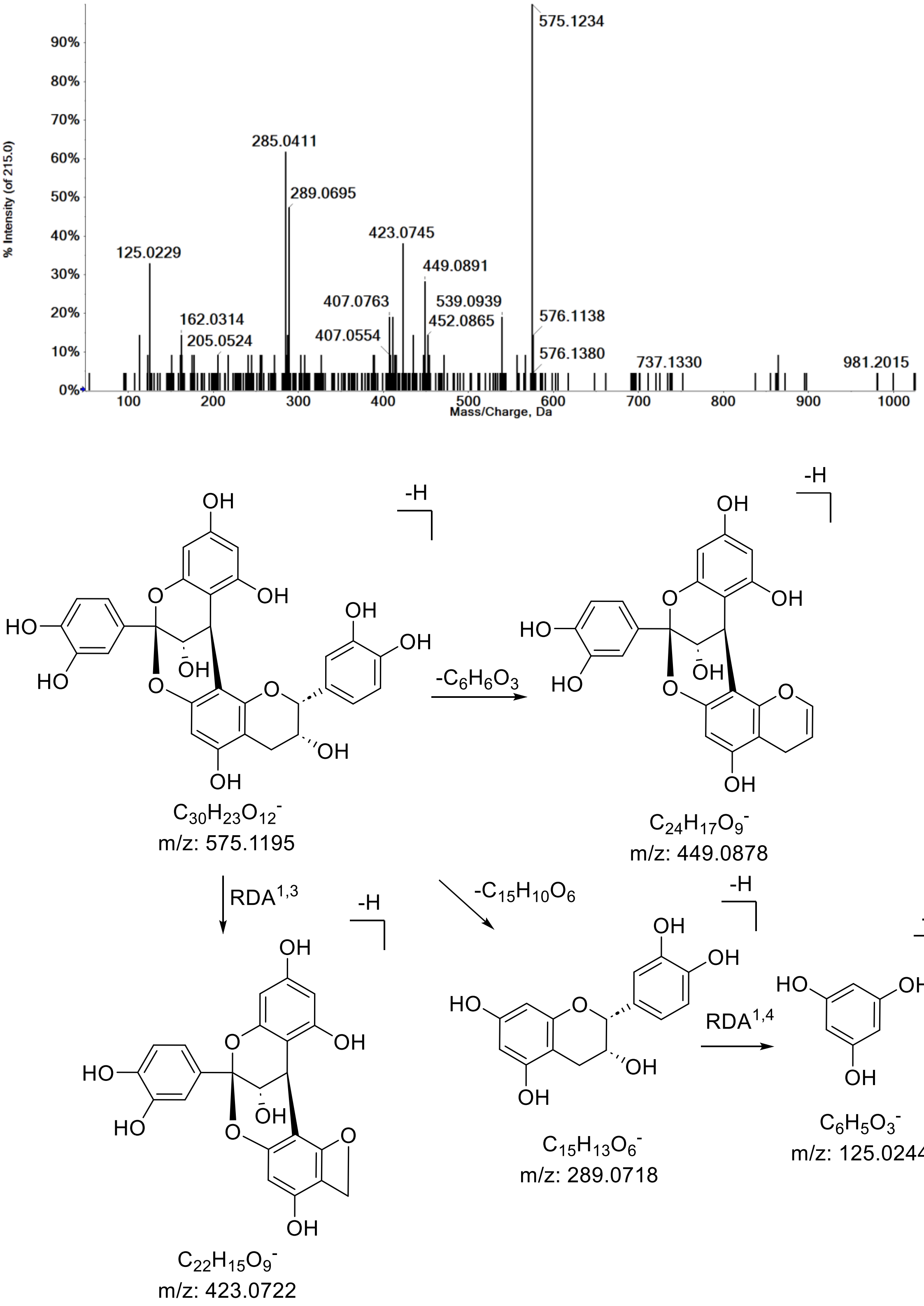

Figure S20. MS/MS spectrum of Procyanidin A5 (peak 20, tR = 6.70 min) in ESI negative ion mode.

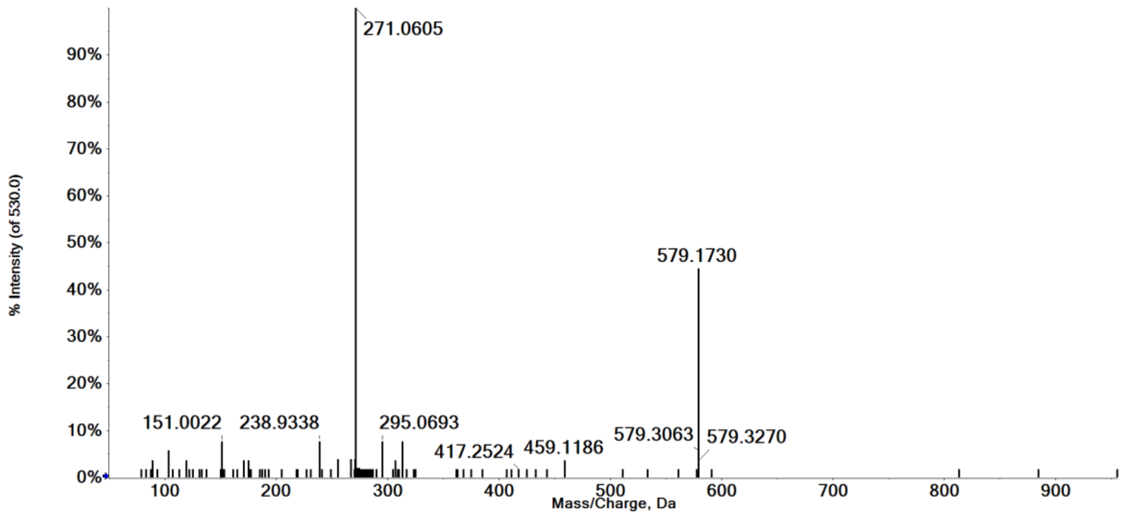

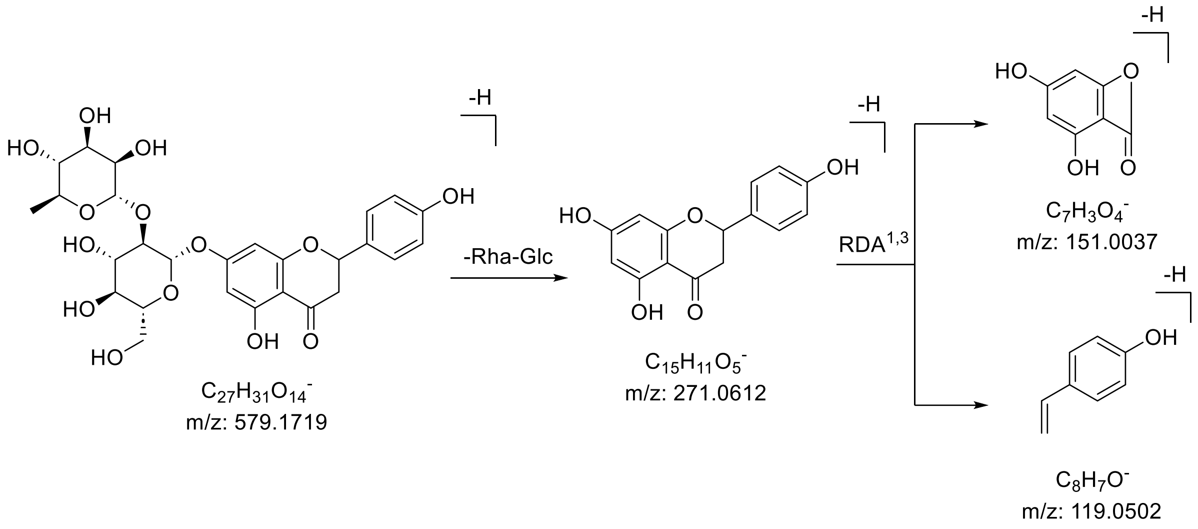

Figure S21. MS/MS spectrum of Naringin (peak 21, tR = 7.24 min) in ESI negative ion mode.

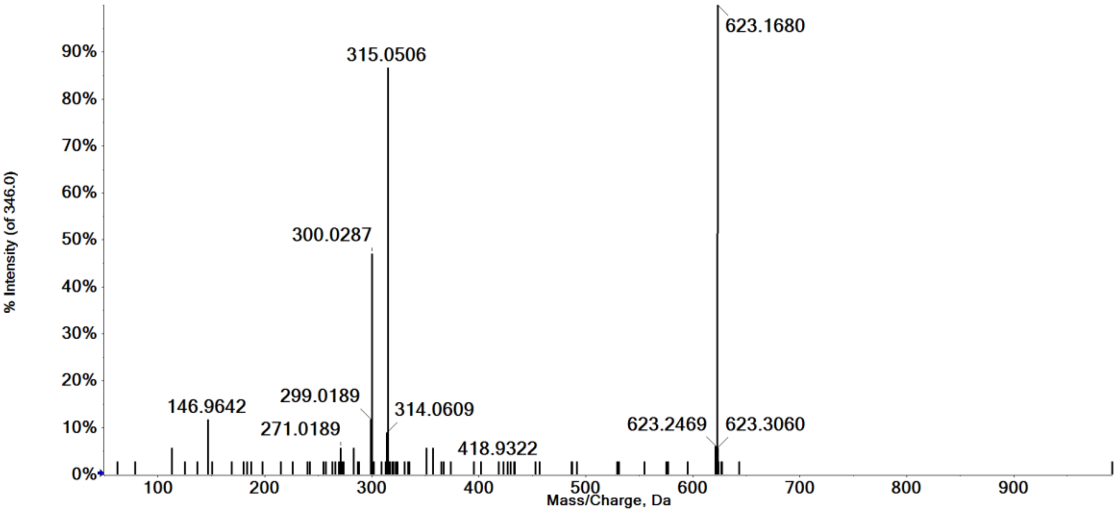

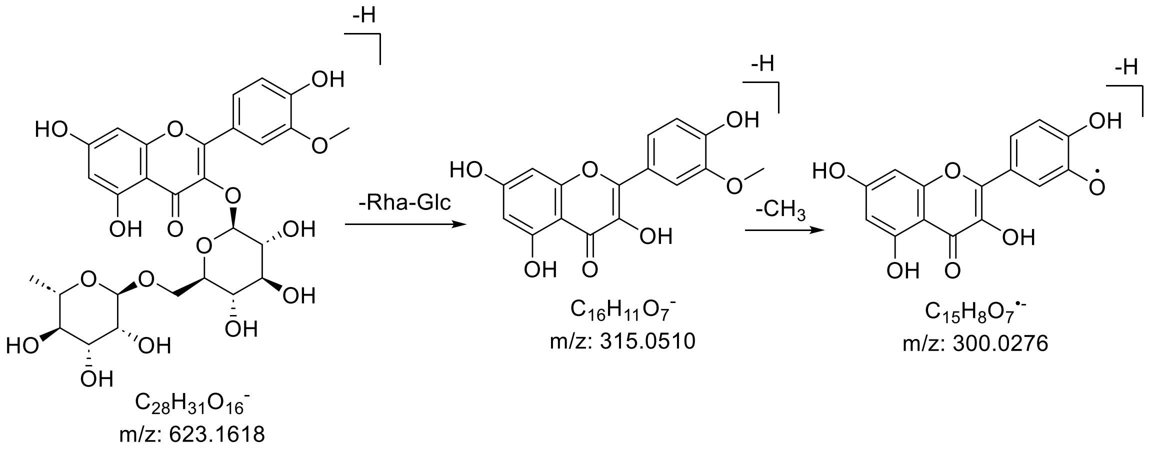

Figure S22. MS/MS spectrum of Narcissin (peak 22, tR = 7.25 min) in ESI negative ion mode.

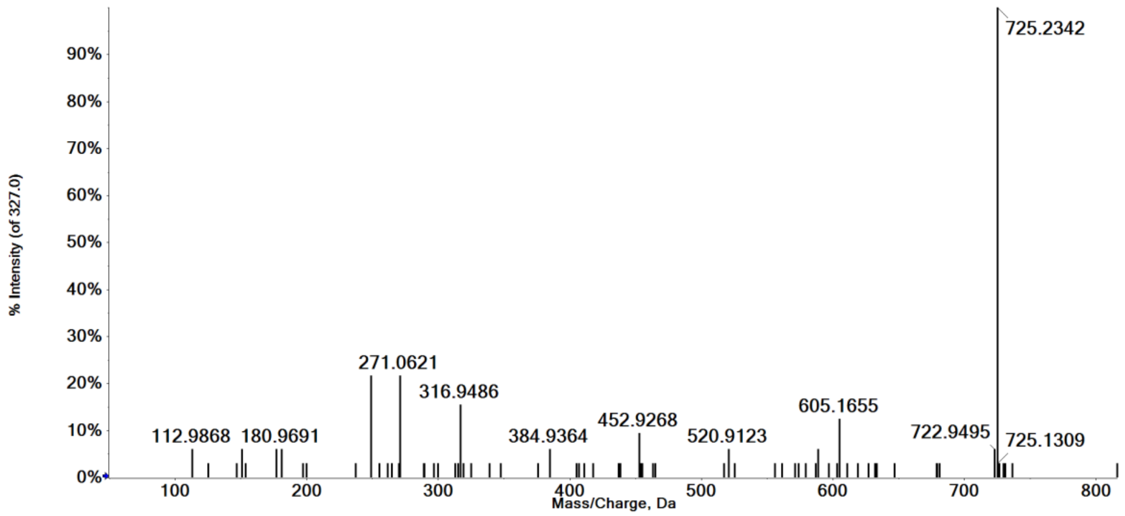

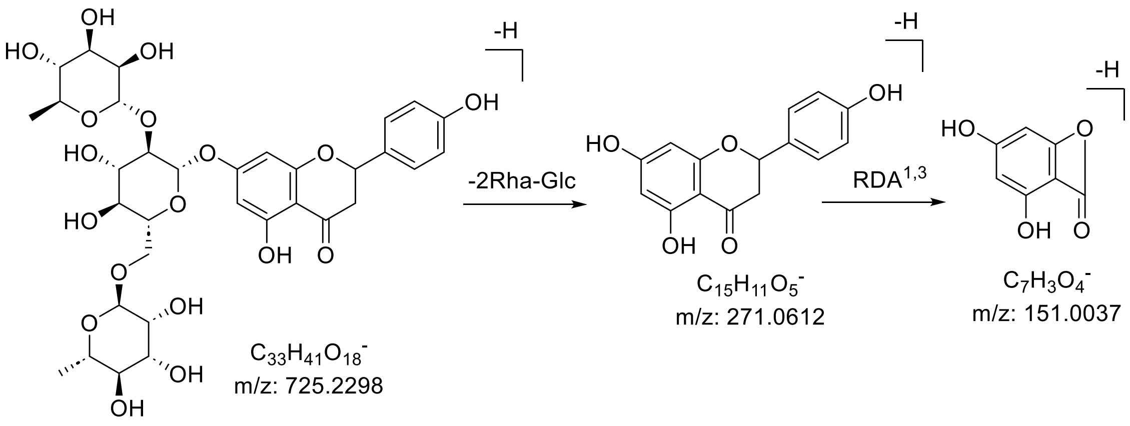

Figure S23. MS/MS spectrum of Naringenin 7-O-(2,6-di-O-α-L-rhamnopyranosyl)-β-D-glucopyranoside (peak 23, tR = 7.60 min) in ESI negative ion mode.

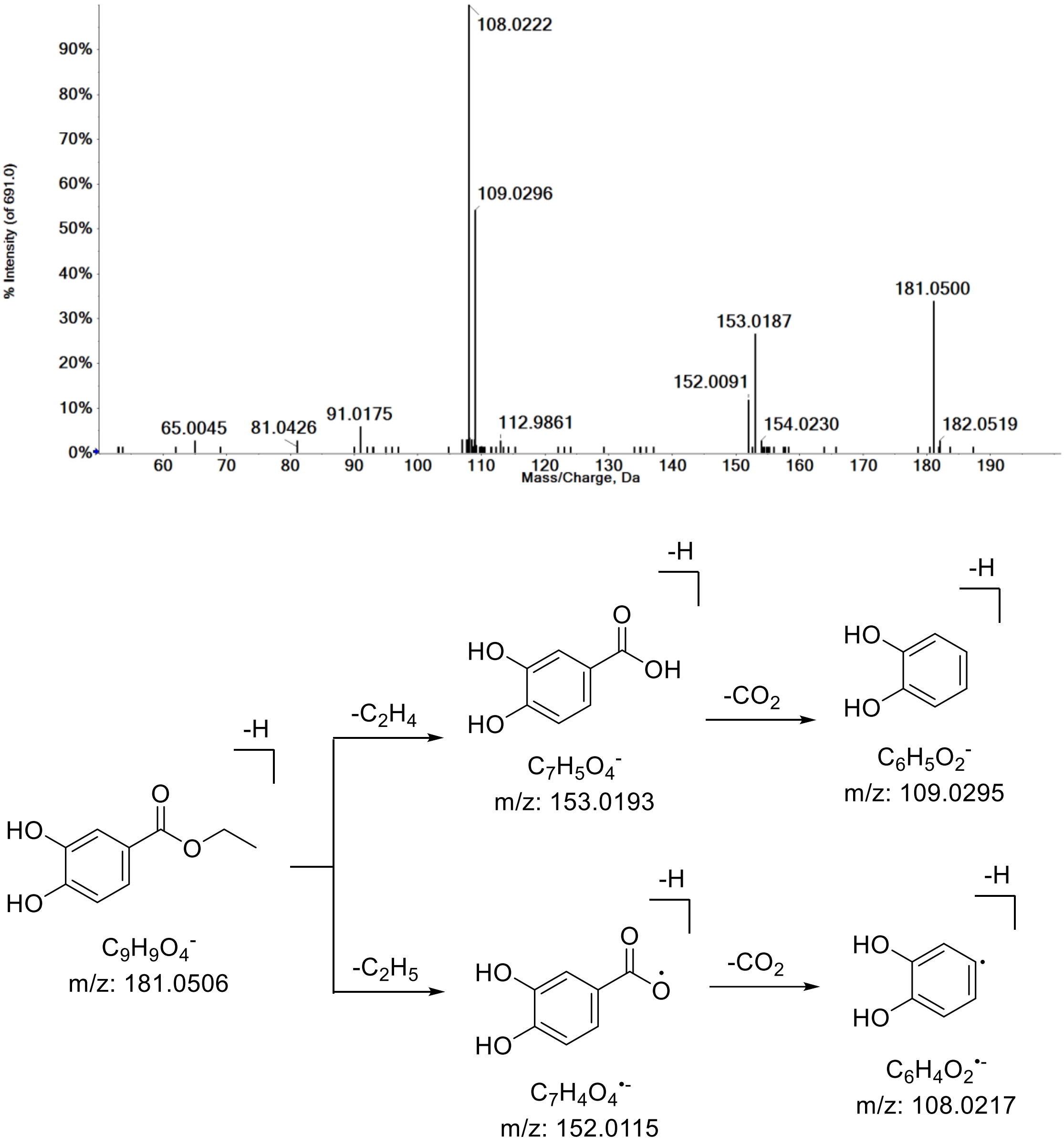

Figure S24. MS/MS spectrum of Ethyl 3,4-dihydroxybenzoate (peak 24, tR = 8.27 min) in ESI negative ion mode.

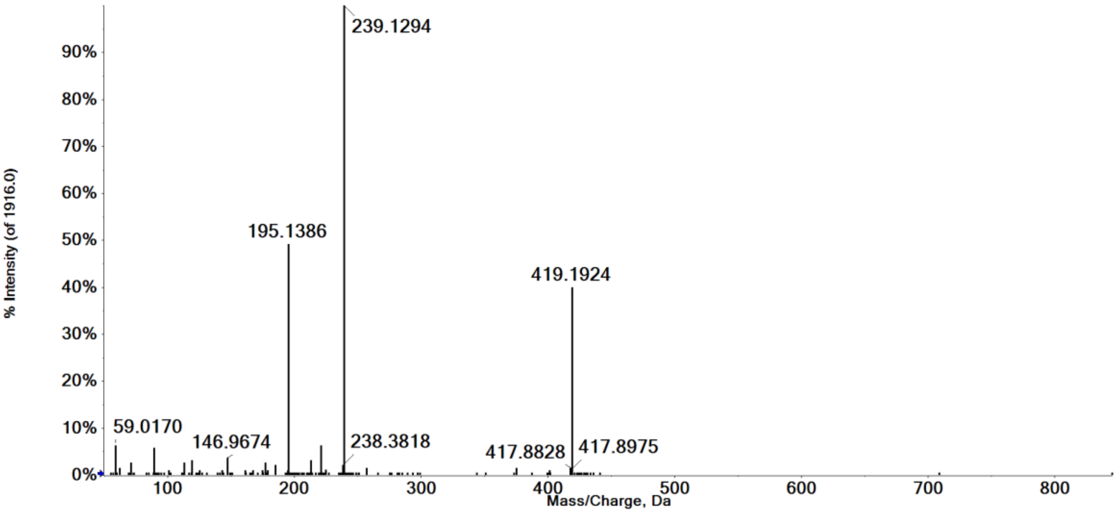

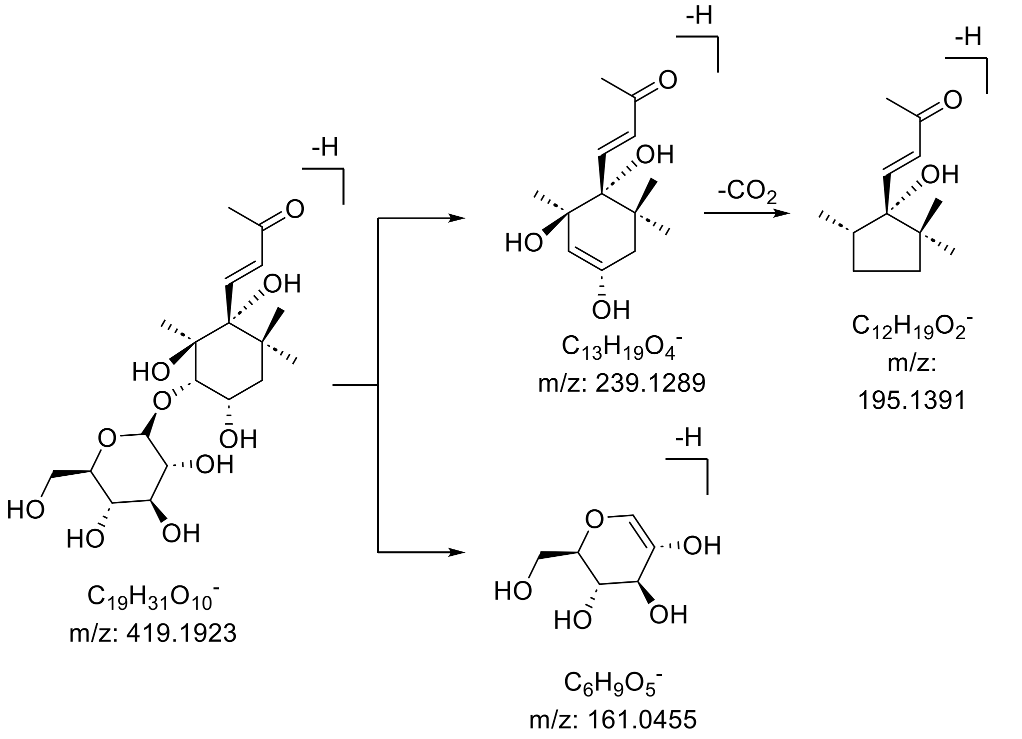

Figure S25. MS/MS spectrum of Youngiajaponicoside C (peak 25, tR = 8.70 min) in ESI negative ion mode.

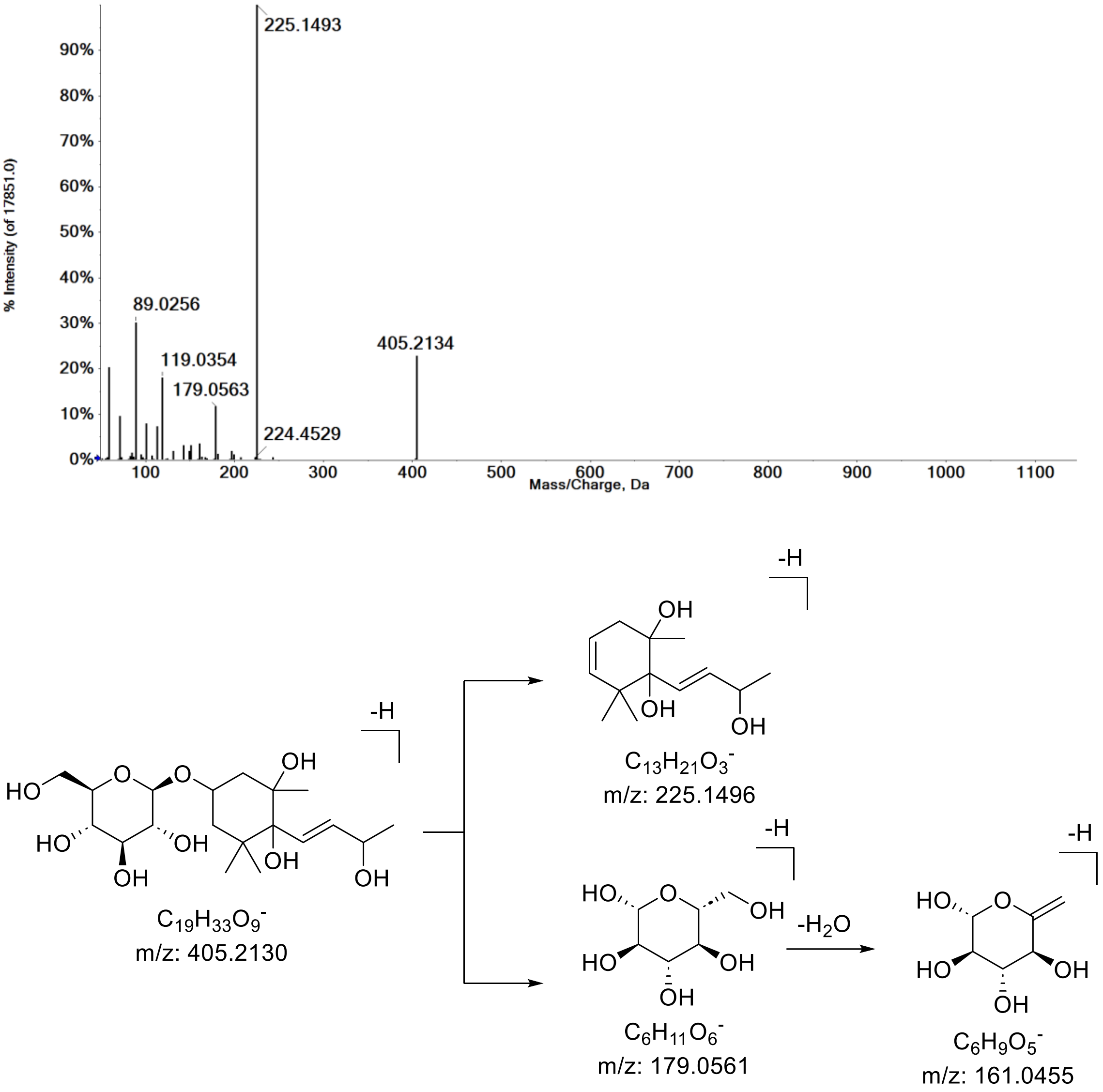

Figure S26. MS/MS spectrum of Kiwiionoside (peak 26, tR = 9.00 min) in ESI negative ion mode.
